# Global burden and temporal trends of kidney cancer among children from 1990 to 2021: an analysis with projections to 2036

**DOI:** 10.3389/fped.2026.1882218

**Published:** 2026-07-09

**Authors:** Kai Liu, Changlin Yang, Feng Chen, Xuhua Qiao, Yujie Geng, Chundong Ji

**Affiliations:** 1 Department of Urology, The Affiliated Hospital of Panzhihua University, Panzhihua, Sichuan Province, China; 2 Department of Respiratory and Critical Care Medicine, Panzhihua Central Hospital, Panzhihua, Sichuan Province, China; 3 Department of Nephrology, The Second People’s Hospital of Guizhou Province, Guiyang, Guizhou Province, China

**Keywords:** children, epidemiology, global burden of disease, health inequality, kidney cancer, Wilms tumor

## Abstract

**Background:**

Childhood kidney cancer (KC), the third most prevalent pediatric solid malignancy, remains incompletely characterized in terms of epidemiological patterns and health inequities. This study aimed to analyze global trends, inequalities, and future burden of childhood KC from 1990 to 2021.

**Methods:**

Data on incidence, mortality, and disability-adjusted life years (DALYs) of KC among children aged 0–14 years were obtained from the Global Burden of Disease study 2021. Data from 1990 to 2021 were comprehensively analyzed by sex, location, age, and socio-demographic index (SDI), incorporating estimated annual percentage change, joinpoint regression, slope index of inequality and concentration index for inequality assessment, and Bayesian age-period-cohort forecasting.

**Results:**

In 2021, there were 9,576 incident cases, 3,063 deaths, and 268,049 DALYs globally due to childhood KC, with corresponding age-standardized incidence rate (ASIR), mortality rate (ASMR), and disability-adjusted life years rate (ASDR) of 0.48, 0.15, and 13.32, respectively. From 1990 to 2021, the global burden of childhood KC declined overall. Joinpoint regression identified a steeper decline in the most recent period, with the ASIR decreasing by 4.87% per year from 2018 to 2021 and the ASMR and ASDR decreasing by 5.12% and 5.23% per year, respectively, from 2019 to 2021. The decline in disease burden has been more pronounced in females than in males. The burden of childhood KC was mainly concentrated among children aged 0–4 years, whose age-specific incidence, mortality, and DALY rates were generally higher than those among children aged 5–9 years and 10–14 years. While no significant association was observed between SDI and ASIR, SDI exhibited a negative correlation with ASMR and ASDR. Inequality metrics showed that childhood KC mortality and DALYs were disproportionately concentrated in lower-SDI countries, and these disparities widened over time. Projections indicate continued declines in incidence and mortality for both sexes over the next 15 years.

**Conclusions:**

Over the past three decades, the burden of childhood KC significantly reduced, yet the rising incidence in lower-income regions warrants attention. Health inequities, characterized by a disproportionate concentration of the disease burden in low-SDI countries, have intensified. Enhanced understanding of pediatric KC epidemiology may aid in its prevention and management.

## Introduction

Cancer remains a major global public health challenge and is one of the leading causes of premature death worldwide, with an estimated 19.96 million new cases and 9.74 million deaths in 2022 (1, 2). Kidney cancer (KC) ranks as the third most common pediatric solid malignancy, accounting for approximately 3.2% to 11.1% of pediatric cancers worldwide (3, 4). The global age-standardized incidence rate of KC in children aged 0–14 years has been reported to be 8.2 per million person-years, with the highest burden observed among children aged 0–4 years (5). In pediatric KC, Wilms tumor (WT) predominates, accounting for more than 80% of cases, whereas renal cell carcinoma is the predominant subtype in adults (6–8). Given the distinct histological profile and younger age at onset, traditional KC risk factors such as smoking, obesity, hypertension, and low fruit and vegetable intake are less relevant in children, whereas genetic susceptibility appears to play a more important role (9, 10). WT has an overall survival rate of 90%, but a quarter of survivors face severe chronic health issues like congestive heart failure, renal failure, and hypertension 25 years post-diagnosis, leading to poor health, dysfunction, and activity limitations (11). Moreover, nearly 30% of pediatric kidney cancers, including relapsed WT, anaplastic WT, malignant rhabdoid tumor, and renal cell carcinoma, remain associated with a poorer prognosis, with survival rates below 70% (12). These characteristics highlight the need for a dedicated assessment of the burden of childhood KC to support the goals of the Countdown to 2030 for Women's, Children's and Adolescents’ Health initiative and advance universal health coverage (13, 14).

Previous studies have improved our understanding of the global burden and epidemiology of childhood KC. Studies of pediatric cancers have rarely provided a detailed evaluation of childhood KC as a separate disease entity (5, 15–17). In addition, findings from adult or all-age KC studies cannot be directly extrapolated to children because childhood KC differs substantially from adult KC in histological composition, epidemiological characteristics, clinical management, and prognosis (9, 18–21). Although population-based studies of childhood renal tumors have described the incidence and mortality of this disease, few have incorporated comprehensive burden metrics within the Global Burden of Disease (GBD) framework (3, 4). Notably, the GBD category of KC encompasses multiple pediatric renal malignancies, including WT, congenital mesoblastic nephroma, malignant rhabdoid tumor, and clear cell sarcoma of the kidney. Therefore, the findings of this study reflect the overall burden of childhood KC rather than WT alone (17). As an effective complement to previous research on the burden and epidemiology of childhood KC, this study investigated the global, regional, and national burden of KC among children aged 0–14 years from 1990 to 2021, focusing on incidence, mortality, and disability-adjusted life years (DALYs). We further analyzed trends by region, age, sex, and socio-demographic index (SDI), examined the association between SDI and disease burden, and projected future burden trends over the next 15 years.

## Materials and methods

### Data source

The GBD 2021 can be accessed through the GHDx online platform (https://vizhub.healthdata.org/gbd-results/), which serves as the raw data source of the burden of KC in children for this cross-sectional study. The GBD 2021 provides estimated results for 371 diseases and injuries and 88 risk factors, covering 204 countries or territories, 25 age groups, and different sexes, with a time span from 1990 to 2021 (22). For each new GBD data update, the complete time series of the population for all years is re-estimated using the most recently available data resources and the latest improved methods, so that the demographic estimates of GBD 2021 replace those of all previous versions (23). In GBD 2021, KC was identified based on the Tenth Revision of the International Classification of Diseases (ICD−10) and ICD−9, with the codes being C64-C65.9, D30.0-D30.1, D41.0-D41.1, 189.0–189.1, 189.5–189.6, 223.0–223.1, and 209.24 (18). In this study, the estimated values and age-standardized rates (ASRs) of incidence, mortality, and DALYs of KC patients aged 0–14 years from 1990 to 2021 were extracted, including their 95% uncertainty intervals (UIs). Our research is based on and strictly adheres to the Guidelines for Accurate and Transparent Health Estimates Reporting (GATHER) (24). Given that the data obtained from the GBD 2021 are publicly available and have been de-identified, this study does not require participants to provide informed consent or ethical approval from an institutional review board.

### Socio-demographic index

The SDI is the geometric mean calculated by the GBD study using three parameters: the lag-distributed income per capita, average years of schooling, and fertility rates for females under 25 (22). It quantifies the level of economic development of a country or region. After normalization, on a scale from 0 to 1, a higher SDI indicates that the country or region has higher income per capita, longer years of education, and lower fertility rates. The SDI is divided into quintiles as high (0.805129–1), high-middle (0.689504–0.805129), middle (0.607679–0.689504), low-middle (0.454743–0.607679), and low (0–0.454743) levels (18).

### Data analysis

All analyses were performed and visualized using R statistical software (version 4.4.0) and Joinpoint Trend Analysis Software. A statistically significant two-sided *P*-value is considered less than 0.05. The unit of the ASR is per 100,000 people. The burden of incidence, mortality, and DALYs of KC in children is measured using both the number and the ASR, and both of them have 95% UI. Each indicator has undergone 500 computational iterations, in which the average value of the estimates is designated as the final estimate, while the 2.5th and 97.5th percentile values are aggregated to form the 95% UI (22). To determine the temporal trends of the burden, we calculated the estimated annual percentage change (EAPC) through the following formula (25):

Y=α+βx+ε;EAPC=100∗(exp(β)−1)

Here, *Y* is the natural logarithm of ASRs, *x* corresponds to calendar years, *ε* represents the error term, and the coefficient *β* is taken from *Y*. The EAPC and its 95% confidence interval (CI) are calculated through the subsequent linear regression model. A positive or negative EAPC indicates an upward or downward trend of ASRs, respectively. We employed the “geom_smooth” function in the “ggplot2” package to construct local regression smoothing models (loess) for evaluating the correlation between ASRs of KC burden and SDI. Additionally, the Spearman's rank-order correlation coefficient test was utilized to calculate the correlation coefficient (r) and *p*-value for the relationship between ASRs and SDI.

We employed joinpoint regression analysis to identify the local temporal trends from 1990 to 2021 via the Joinpoint Trend Analysis Software. The joinpoint regression model conducts segmented regression by identifying multiple “joinpoints” in the time-series data, which represent significant change points in the trend (26). The calculated annual percentage change (APC) and its 95% CI for each sub-period can present the trend changes in greater detail. Concurrently, the average annual percentage change (AAPC) was calculated by means of the weighted average of the span widths of the regression coefficients of the piecewise interval regression model (27). A statistically significant *P*-value was less than 0.05.

The slope index of inequality (SII) and the concentration index are used to assess the absolute and relative inequalities in the burden of KC among nations with varying SDI levels (28). The SII is the slope of the regression line of the disease burden indicator and the relative distribution of SDI. It is calculated by performing linear regression of the ASRs of KC disease burden and the weighted position ranking related to SDI for each country. This particular weighted position ranking is precisely delineated by the midpoint of the cumulative range of the population, which is ranked in accordance with the SDI. It reflects the health differences between the group with the lowest SDI and the group with the highest SDI (29). The Lorenz concentration curve is obtained by fitting the cumulative proportion of incidence, mortality, and DALYs and the cumulative relative distribution of the population sorted by SDI. The concentration index is calculated by numerically integrating the area under the curve. A Lorenz curve below the diagonal line indicates that the burden of health indicators is concentrated in countries with a high SDI, and the concentration index is positive. Conversely, the concentration index is negative. An absolute value of the concentration index between 0.2 and 0.3 indicates a relatively high degree of relative inequality (30).

We employed the Bayesian age-period-cohort analysis (BAPC) model incorporating the integrated nested Laplace approximation (INLA), which has been demonstrated to exhibit superior confidence interval coverage and precision compared to the age-period-cohort model, to forecast the burden of KC globally from 2022 to 2036 (19). The merit of this predictive method resides in its capacity to precisely prognosticate the prospective disease burden, predicated upon a comprehensive consideration of the intricate interrelationships subsisting among age, period, and cohort effects. Furthermore, this approach is efficacious in approximating the marginal posterior distribution, thereby obviating a multiplicity of mixing and convergence conundrums that are typically engendered by the Markov Chain Monte Carlo sampling techniques within the purview of the Bayesian method (29).

## Results

### Incidence trend of kidney cancer in children

**Figure 1:**
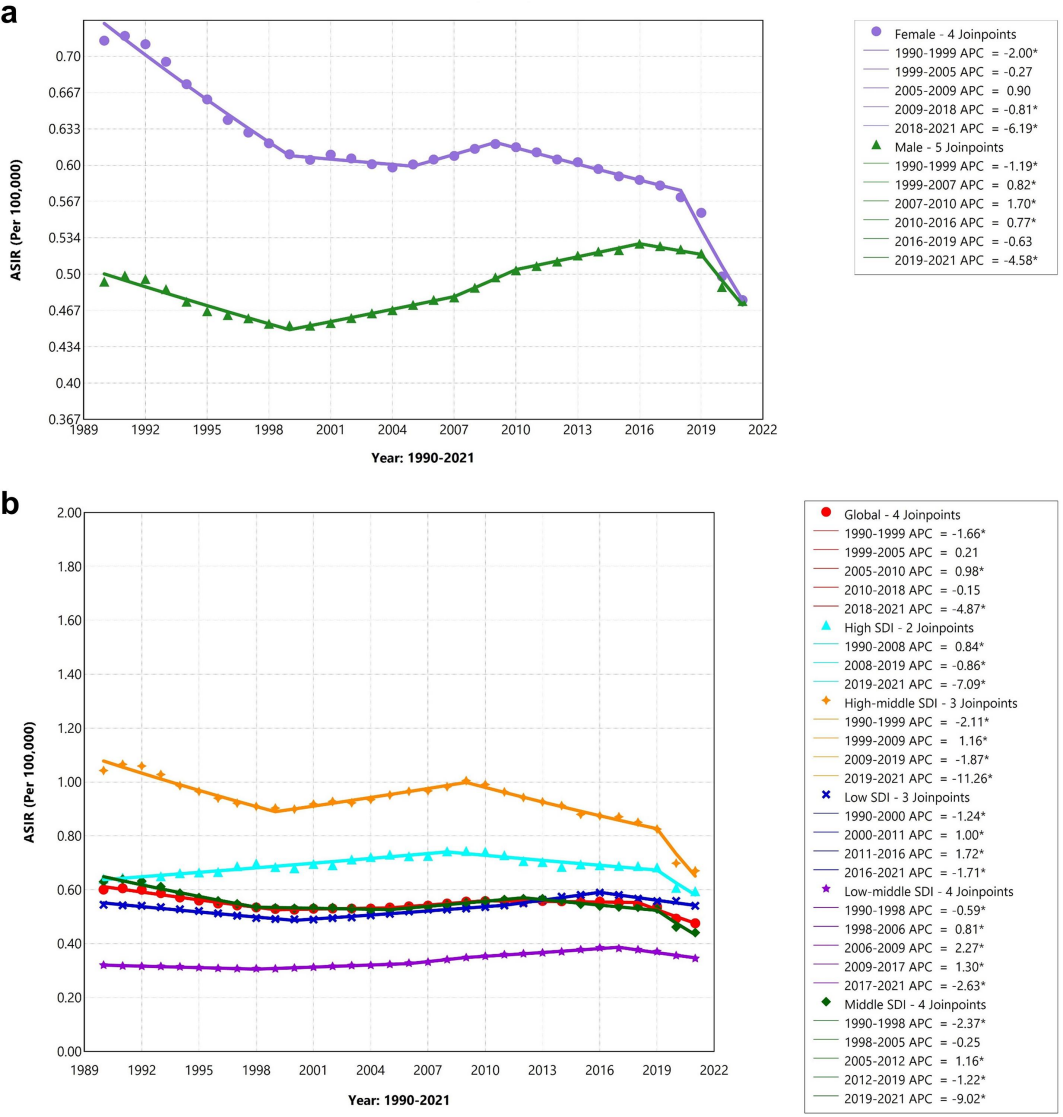
Joinpoint regression analysis of age-standardized incidence rates of childhood kidney cancer by sex **(a)** and across different SDI regions **(b)**, 1990∼2021. APC, annual percentage change; ASIR, age-standardized incidence rate; SDI, socio-demographic index.

From 1990 to 2021, the global incidence cases of KC in children decreased from 10,450 cases (95% UI, 8,482–12,416) to 9,576 cases (95% UI, 7,561–11,649), and the corresponding age-standardized incidence rate (ASIR) decreased from 0.60 (95% UI, 0.49–0.71) to 0.48 (95% UI, 0.38–0.58); EAPC was −0.28 (95% CI, −0.44 –−0.12) (Table 1). The ASIR of KC in males showed an upward trend (EAPC = 0.32; 95% CI, 0.16–0.47), while that in females showed the opposite trend (EAPC = −0.79; 95% CI, −0.96 –−0.62) (Table 1 and Supplementary Figure S1a). Joinpoint regression analysis showed that the AAPC of the ASIR for females was −1.37 (95% CI, −1.58 –−1.15), while that for males was −0.19 (95% CI, −0.44–0.06). The ASIRs of both females and males decreased significantly in the recent three years, with the APC being −6.19 (95% CI, −7.39 –−4.97) for females and −4.58 (95% CI, −6.97 –−2.14) for males (Supplementary Table S1 and Figure 1a). The number of incident cases of KC in children aged 2 to 4 years was the highest from 1990 to 2021, with 3,036 cases in 2021. From 1990 to 2021, the incidence rate of KC in each age group of children under 5 years old was higher than that in children aged 5–9 years and 10–14 years. From 1990 to 2021, the incident cases and the incidence rates of KC in children of all age groups under 5 years old decreased (Table 1, Supplementary Figure S2a and Supplementary Figure S2b).

**Table 1:** The temporal trends of incidence, mortality and disability-adjusted life years of kidney cancer in children from 1990 to 2021 globally and regionally.

Regions	1990		2021		EAPC of ASIR, % (95% CI)	1990		2021		EAPC of ASMR, % (95% CI)	1990		2021		EAPC of ASDR, % (95% CI)
	Incidence cases, N(95% UI)	ASIR, Per 100,000 (95%UI)	Incidence cases, N(95% UI)	ASIR, Per 100,000 (95%UI)		Mortality cases, N(95% UI)	ASMR, Per 100,000 (95%UI)	Mortality cases, N(95% UI)	ASMR, Per 100,000 (95%UI)		Number of DALYs, N(95% UI)	ASDR, Per 100,000 (95%UI)	Number of DALYs, N(95% UI)	ASDR, Per 100,000 (95%UI)	
Global	10,450 (8,482,12,416)	0.6 (0.49,0.71)	9,576 (7,561,11,649)	0.48 (0.38,0.58)	−0.28 (−0.44,−0.12)	4,136 (3,292,5,010)	0.24 (0.19,0.29)	3,063 (2,252,3,898)	0.15 (0.11,0.19)	−1.02 (−1.18,−0.87)	362,175 (287,018,438,939)	20.82 (16.5,25.24)	268,049 (196,876,341,769)	13.32 (9.79,16.99)	−1.01 (−1.17,−0.86)
Sex															
Male	4,408 (3,635,5,557)	0.49 (0.41,0.62)	4,937 (3,749,6,123)	0.48 (0.36,0.59)	0.32 (0.16,0.47)	2,021 (1,618,2,642)	0.23 (0.18,0.3)	1,855 (1,317,2,367)	0.18 (0.13,0.23)	−0.36 (−0.52,−0.2)	176,516 (141,295,230,879)	19.76 (15.81,25.84)	162,201 (115,318,207,450)	15.62 (11.11,19.98)	−0.34 (−0.51,−0.18)
Female	6,042 (4,577,7,780)	0.71 (0.54,0.92)	4,639 (3,809,5,785)	0.48 (0.39,0.59)	−0.79 (−0.96,−0.62)	2,116 (1,532,2,849)	0.25 (0.18,0.34)	1,208 (927,1,570)	0.12 (0.1,0.16)	−1.81 (−1.96,−1.66)	185,659 (134,020,250,351)	21.95 (15.85,29.6)	105,848 (81,335,137,619)	10.87 (8.35,14.13)	−1.8 (−1.95,−1.65)
Age groups															
<28 days	196 (180,216)	1.95 (1.79,2.15)	177 (146,210)	1.81 (1.5,2.15)	0.08 (−0.01,0.18)	79 (73,87)	0.78 (0.72,0.86)	55 (45,65)	0.57 (0.46,0.67)	−0.97 (−1.01,−0.92)	7,179 (6,597,7,885)	71.51 (65.71,78.53)	5,045 (4,138,5,983)	51.77 (42.46,61.4)	−0.95 (−0.99,−0.9)
1–5 months	546 (467,641)	1 (0.86,1.17)	467 (366,572)	0.87 (0.68,1.06)	−0.29 (−0.37,−0.21)	218 (186,253)	0.4 (0.34,0.46)	142 (105,177)	0.27 (0.2,0.33)	−1.21 (−1.3,−1.12)	19,840 (16,928,23,034)	36.34 (31.01,42.19)	13,023 (9,644,16,187)	24.23 (17.94,30.11)	−1.19 (−1.29,−1.1)
6–11 months	941 (727,1,159)	1.49 (1.15,1.84)	726 (506,967)	1.15 (0.8,1.53)	−0.54 (−0.7,−0.38)	376 (296,456)	0.6 (0.47,0.72)	268 (177,365)	0.42 (0.28,0.58)	−0.84 (−1,−0.67)	34,055 (26,802,41,498)	53.96 (42.47,65.75)	24,258 (16,106,33,206)	38.39 (25.49,52.55)	−0.83 (−1,−0.67)
12–23 months	2,076 (1,655,2,549)	1.67 (1.33,2.05)	1,749 (1,295,2,212)	1.36 (1.01,1.72)	−0.17 (−0.36,0.03)	826 (642,1,036)	0.66 (0.52,0.83)	588 (399,778)	0.46 (0.31,0.61)	−0.86 (−1.07,−0.65)	74,154 (57,668,92,895)	59.5 (46.28,74.54)	52,978 (36,028,69,948)	41.26 (28.06,54.47)	−0.85 (−1.05,−0.64)
2–4 years	3,783 (2,709,4,764)	1.03 (0.74,1.3)	3,036 (2,151,4,006)	0.75 (0.53,0.99)	−0.64 (−0.78,−0.51)	1,641 (1,142,2,102)	0.45 (0.31,0.57)	1,179 (803,1,601)	0.29 (0.2,0.4)	−1.13 (−1.24,−1.02)	144,291 (100,204,184,986)	39.26 (27.26,50.33)	103,552 (70,523,140,765)	25.69 (17.5,34.92)	−1.12 (−1.24,−1.01)
5–9 years	2,148 (1,930,2,369)	0.37 (0.33,0.41)	2,323 (2,034,2,636)	0.34 (0.3,0.38)	−0.02 (−0.13,0.08)	741 (655,831)	0.13 (0.11,0.14)	580 (492,663)	0.08 (0.07,0.1)	−1.07 (−1.15,−0.99)	62,451 (55,212,70,088)	10.7 (9.46,12.01)	49,220 (41,820,56,282)	7.16 (6.09,8.19)	−1.05 (−1.13,−0.97)
10–14 years	761 (716,810)	0.14 (0.13,0.15)	1,099 (985,1,210)	0.16 (0.15,0.18)	0.65 (0.56,0.74)	256 (239,274)	0.05 (0.04,0.05)	250 (220,280)	0.04 (0.03,0.04)	−0.59 (−0.66,−0.52)	20,206 (18,902,21,638)	3.77 (3.53,4.04)	19,973 (17,478,22,302)	3 (2.62,3.35)	−0.56 (−0.63,−0.49)
SDI regions															
High SDI	1,192 (1,143,1,239)	0.64 (0.62,0.67)	1,027 (949,1,103)	0.6 (0.55,0.64)	0.08 (−0.13,0.28)	245 (235,255)	0.13 (0.13,0.14)	123 (114,131)	0.07 (0.07,0.08)	−1.69 (−1.79,−1.58)	21,390 (20,444,22,303)	11.51 (11,12)	10,874 (10,074,11,619)	6.3 (5.84,6.73)	−1.63 (−1.74,−1.52)
High-middle SDI	2,853 (2,388,3,387)	1.04 (0.87,1.24)	1,549 (1,310,1,808)	0.67 (0.57,0.78)	−0.74 (−1.01,−0.47)	878 (738,1,052)	0.32 (0.27,0.38)	244 (207,277)	0.11 (0.09,0.12)	−3.11 (−3.29,−2.93)	77,004 (64,696,92,425)	28.14 (23.64,33.78)	21,397 (18,172,24,481)	9.27 (7.87,10.6)	−3.08 (−3.26,−2.89)
Middle SDI	3,637 (2,987,4,319)	0.63 (0.52,0.75)	2,501 (2,037,3,017)	0.44 (0.36,0.53)	−0.53 (−0.74,−0.31)	1,473 (1,243,1,732)	0.26 (0.22,0.3)	662 (537,793)	0.12 (0.09,0.14)	−1.99 (−2.17,−1.82)	128,721 (107,893,152,060)	22.3 (18.69,26.34)	57,526 (46,506,69,379)	10.15 (8.2,12.24)	−2 (−2.18,−1.82)
Low-middle SDI	1,514 (1,068,1,940)	0.32 (0.23,0.41)	2,003 (1,521,2,523)	0.35 (0.26,0.44)	0.72 (0.57,0.87)	772 (541,1,004)	0.16 (0.11,0.21)	794 (587,1,001)	0.14 (0.1,0.17)	−0.12 (−0.25,0.01)	67,487 (47,369,87,874)	14.29 (10.03,18.61)	69,424 (51,356,87,572)	11.97 (8.86,15.1)	−0.12 (−0.25,0.02)
Low SDI	1,247 (739,1,717)	0.54 (0.32,0.75)	2,489 (1,522,3,497)	0.54 (0.33,0.76)	0.34 (0.17,0.51)	766 (459,1,038)	0.33 (0.2,0.45)	1,238 (769,1,751)	0.27 (0.17,0.38)	−0.38 (−0.53,−0.22)	67,293 (40,307,91,132)	29.4 (17.61,39.81)	108,628 (67,581,153,891)	23.6 (14.68,33.44)	−0.38 (−0.53,−0.22)
GBD regions															
Andean Latin America	95 (77,114)	0.64 (0.52,0.77)	84 (63,110)	0.46 (0.35,0.61)	−0.7 (−0.92,−0.49)	50 (41,59)	0.34 (0.27,0.4)	26 (20,34)	0.15 (0.11,0.19)	−2.42 (−2.61,−2.24)	4,328 (3,487,5,126)	29.14 (23.48,34.51)	2,264 (1,728,2,936)	12.51 (9.55,16.23)	−2.44 (−2.63,−2.25)
Australasia	25 (22,28)	0.54 (0.48,0.62)	24 (20,30)	0.42 (0.35,0.52)	−0.1 (−0.72,0.54)	6 (5,6)	0.13 (0.12,0.14)	3 (3,4)	0.05 (0.05,0.06)	−2.18 (−2.64,−1.72)	508 (464,553)	11.07 (10.11,12.05)	270 (229,314)	4.71 (4,5.48)	−2.13 (−2.6,−1.65)
Caribbean	75 (52,99)	0.65 (0.46,0.86)	64 (46,94)	0.56 (0.4,0.82)	−0.17 (−0.3,−0.05)	38 (25,52)	0.33 (0.22,0.45)	27 (18,42)	0.24 (0.16,0.37)	−0.74 (−0.94,−0.55)	3,256 (2,117,4,480)	28.53 (18.55,39.26)	2,346 (1,565,3,661)	20.39 (13.6,31.82)	−0.75 (−0.95,−0.56)
Central Asia	158 (127,195)	0.63 (0.51,0.78)	140 (113,171)	0.51 (0.41,0.62)	0.04 (−0.31,0.39)	67 (54,83)	0.27 (0.22,0.33)	49 (40,60)	0.18 (0.14,0.22)	−0.67 (−0.92,−0.42)	5,850 (4,707,7,269)	23.41 (18.83,29.09)	4,256 (3,425,5,225)	15.38 (12.37,18.88)	−0.67 (−0.93,−0.41)
Central Europe	162 (143,178)	0.55 (0.49,0.6)	76 (67,86)	0.43 (0.38,0.49)	−0.26 (−0.58,0.06)	57 (51,62)	0.19 (0.17,0.21)	16 (14,18)	0.09 (0.08,0.1)	−2.07 (−2.34,−1.8)	4,937 (4,402,5,403)	16.74 (14.93,18.33)	1,375 (1,214,1,550)	7.77 (6.86,8.76)	−2.04 (−2.3,−1.77)
Central Latin America	392 (365,425)	0.61 (0.57,0.66)	270 (217,333)	0.43 (0.34,0.53)	−0.44 (−0.67,−0.2)	191 (177,207)	0.3 (0.28,0.32)	84 (68,104)	0.13 (0.11,0.16)	−1.88 (−2.08,−1.68)	16,510 (15,340,17,928)	25.64 (23.83,27.85)	7,235 (5,852,8,981)	11.4 (9.22,14.15)	−1.89 (−2.09,−1.69)
Central Sub-Saharan Africa	50 (23,84)	0.2 (0.09,0.33)	80 (53,116)	0.14 (0.09,0.2)	−0.75 (−0.97,−0.53)	31 (14,53)	0.12 (0.06,0.21)	40 (27,58)	0.07 (0.05,0.1)	−1.44 (−1.64,−1.25)	2,745 (1,233,4,661)	10.85 (4.87,18.43)	3,491 (2,303,5,028)	5.95 (3.93,8.57)	−1.47 (−1.67,−1.27)
East Asia	3,380 (2,770,4,152)	1.02 (0.84,1.26)	1,653 (1,251,2,063)	0.62 (0.47,0.77)	−0.84 (−1.14,−0.54)	1,309 (1,080,1,603)	0.4 (0.33,0.49)	300 (223,376)	0.11 (0.08,0.14)	−3.49 (−3.7,−3.28)	114,941 (94,387,141,197)	34.85 (28.62,42.81)	26,234 (19,401,32,834)	9.81 (7.26,12.28)	−3.48 (−3.7,−3.26)
Eastern Europe	560 (521,606)	1.09 (1.01,1.18)	177 (162,195)	0.5 (0.46,0.55)	−1.36 (−1.85,−0.86)	147 (138,158)	0.29 (0.27,0.31)	33 (31,36)	0.09 (0.09,0.1)	−2.65 (−3.06,−2.23)	12,858 (12,051,13,838)	24.99 (23.42,26.89)	2,907 (2,676,3,172)	8.2 (7.55,8.95)	−2.59 (−3.02,−2.16)
Eastern Sub-Saharan Africa	694 (434,971)	0.77 (0.48,1.07)	1,117 (591,1,666)	0.63 (0.33,0.93)	−0.24 (−0.46,−0.03)	432 (269,602)	0.48 (0.3,0.66)	544 (289,817)	0.3 (0.16,0.46)	−1.05 (−1.24,−0.85)	38,017 (23,706,52,892)	41.97 (26.17,58.4)	47,786 (25,317,71,704)	26.78 (14.19,40.19)	−1.05 (−1.24,−0.85)
High-income Asia Pacific	101 (90,112)	0.29 (0.25,0.32)	68 (59,79)	0.31 (0.26,0.35)	0.39 (0.04,0.75)	28 (25,32)	0.08 (0.07,0.09)	9 (8,10)	0.04 (0.04,0.05)	−2.05 (−2.22,−1.88)	2,448 (2,199,2,726)	6.95 (6.25,7.74)	823 (742,910)	3.67 (3.31,4.06)	−1.97 (−2.15,−1.78)
High-income North America	520 (502,538)	0.84 (0.81,0.87)	425 (394,459)	0.65 (0.6,0.7)	−0.66 (−0.85,−0.46)	73 (71,74)	0.12 (0.12,0.12)	44 (41,47)	0.07 (0.06,0.07)	−1.66 (−1.78,−1.55)	6,382 (6,224,6,547)	10.35 (10.09,10.61)	3,860 (3,606,4,158)	5.88 (5.49,6.34)	−1.62 (−1.74,−1.51)
North Africa and Middle East	1,166 (721,1,650)	0.83 (0.51,1.17)	1,116 (874,1,385)	0.61 (0.48,0.76)	−0.3 (−0.57,−0.03)	297 (197,405)	0.21 (0.14,0.29)	197 (159,243)	0.11 (0.09,0.13)	−1.62 (−1.83,−1.4)	26,206 (17,273,35,687)	18.65 (12.3,25.4)	17,329 (14,052,21,253)	9.45 (7.66,11.59)	−1.61 (−1.83,−1.39)
Oceania	4 (2,6)	0.13 (0.09,0.21)	8 (5,13)	0.16 (0.1,0.25)	0.54 (0.34,0.75)	1 (1,2)	0.05 (0.04,0.08)	3 (2,5)	0.06 (0.04,0.1)	0.56 (0.32,0.8)	124 (84,191)	4.62 (3.14,7.12)	279 (187,443)	5.49 (3.67,8.72)	0.56 (0.32,0.8)
South Asia	804 (493,1,104)	0.19 (0.11,0.25)	1,008 (705,1,357)	0.2 (0.14,0.27)	0.51 (0.38,0.63)	477 (290,658)	0.11 (0.07,0.15)	438 (300,592)	0.09 (0.06,0.12)	−0.53 (−0.63,−0.42)	41,707 (25,382,57,535)	9.62 (5.86,13.28)	38,319 (26,201,52,034)	7.56 (5.17,10.26)	−0.52 (−0.63,−0.41)
Southeast Asia	676 (395,955)	0.4 (0.23,0.56)	663 (521,821)	0.38 (0.3,0.48)	0.03 (−0.04,0.1)	257 (149,371)	0.15 (0.09,0.22)	209 (164,261)	0.12 (0.1,0.15)	−0.51 (−0.59,−0.43)	22,321 (12,845,32,404)	13.07 (7.52,18.98)	18,124 (14,172,22,674)	10.5 (8.21,13.13)	−0.52 (−0.6,−0.44)
Southern Latin America	142 (124,166)	0.95 (0.83,1.11)	149 (121,181)	1.03 (0.83,1.25)	0.62 (0.3,0.95)	25 (23,28)	0.17 (0.15,0.19)	15 (13,18)	0.1 (0.09,0.12)	−1.09 (−1.3,−0.88)	2,197 (1,977,2,427)	14.72 (13.24,16.26)	1,330 (1,123,1,560)	9.18 (7.75,10.76)	−1.04 (−1.25,−0.82)
Southern Sub-Saharan Africa	69 (47,92)	0.33 (0.23,0.44)	100 (79,121)	0.41 (0.33,0.5)	1.28 (0.8,1.77)	34 (23,45)	0.17 (0.11,0.22)	41 (33,51)	0.17 (0.14,0.21)	0.68 (0.28,1.08)	2,957 (2,019,3,909)	14.29 (9.76,18.89)	3,554 (2,813,4,405)	14.77 (11.69,18.3)	0.7 (0.29,1.11)
Tropical Latin America	317 (282,350)	0.59 (0.53,0.65)	226 (184,268)	0.45 (0.37,0.53)	−0.09 (−0.5,0.34)	153 (136,170)	0.29 (0.25,0.32)	72 (58,86)	0.14 (0.12,0.17)	−1.41 (−1.82,−1.01)	13,291 (11,804,14,699)	24.79 (22.02,27.42)	6,163 (4,963,7,371)	12.28 (9.89,14.68)	−1.43 (−1.84,−1.03)
Western Europe	487 (459,520)	0.69 (0.65,0.73)	446 (397,494)	0.66 (0.58,0.73)	0.09 (−0.14,0.31)	103 (98,107)	0.14 (0.14,0.15)	52 (47,57)	0.08 (0.07,0.08)	−1.84 (−1.97,−1.71)	8,983 (8,587,9,321)	12.65 (12.09,13.13)	4,628 (4,195,5,094)	6.79 (6.16,7.48)	−1.77 (−1.91,−1.64)
Western Sub-Saharan Africa	576 (425,732)	0.65 (0.48,0.83)	1,682 (1,050,2,349)	0.78 (0.49,1.09)	1.11 (0.93,1.28)	360 (260,468)	0.41 (0.3,0.53)	859 (541,1,181)	0.4 (0.25,0.55)	0.4 (0.25,0.56)	31,609 (22,923,41,182)	35.97 (26.08,46.86)	75,476 (47,520,103,562)	35.14 (22.13,48.22)	0.4 (0.25,0.56)

UI, uncertainty interval; ASIR, age-standardized incidence rate; EAPC, estimated annual percentage change; CI, confidence interval; ASMR, age-standardized mortality rate; DALYs, disability-adjusted life years; ASDR, age-standardized disability-adjusted life years rate; GBD, the global burden of disease; SDI, socio-demographic index.

**Figure 2:**
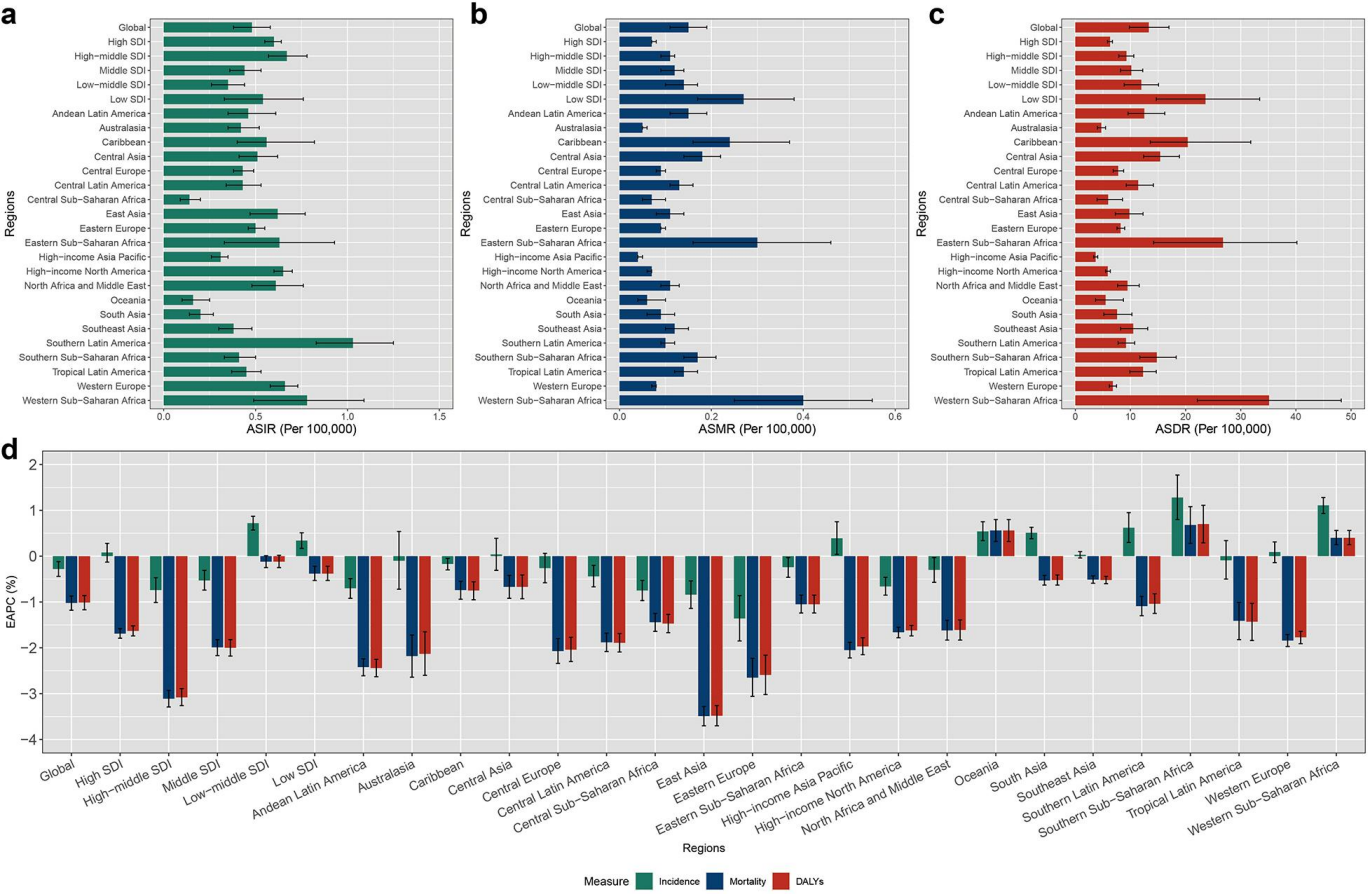
Age-standardized incidence **(a)**, mortality **(b)**, and DALYs**(c)** rates and corresponding EAPCs **(d)** of the global burden of childhood kidney cancer in 2021 across global, 5 SDI regions, and 21 GBD regions. ASIR, age-standardized incidence rate; ASMR, age-standardized mortality rate; ASDR, age-standardized DALYs rate; EAPC, estimated annual percentage change; DALYs, disability-adjusted life years; SDI, socio-demographic index.

**Figure 3:**
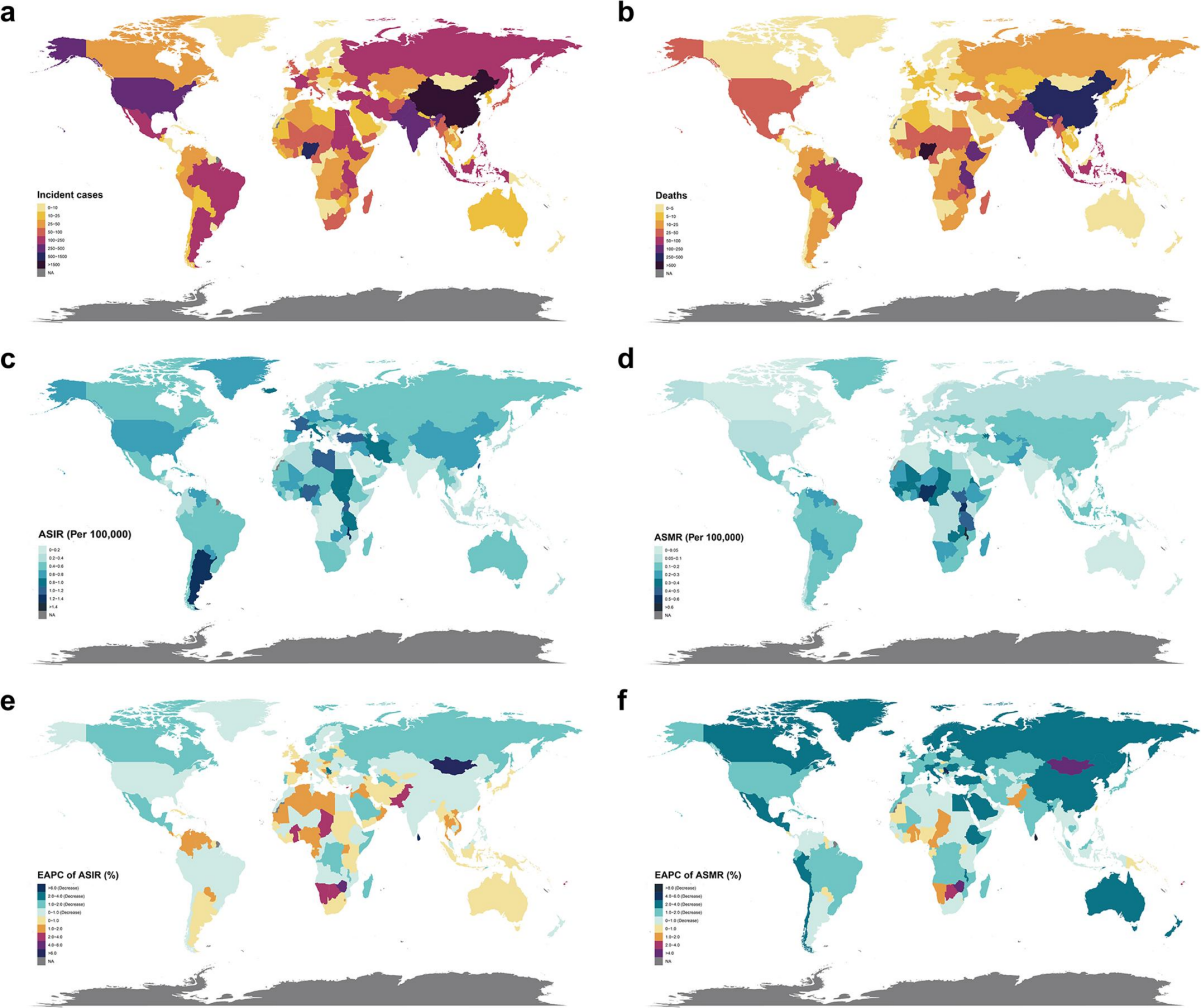
Incidence, mortality, and their trends of childhood kidney cancer in 204 countries and territories worldwide, 2021. **(a)** The incidence case of kidney cancer. **(b)** The mortality case of kidney cancer. **(c)** The age-standardized incidence rate of kidney cancer. **(d)** The age-standardized mortality rate of kidney cancer. **(e)** The EAPC of ASIR. **(f)** The EAPC of ASMR. ASIR, age-standardized incidence rate; ASMR, age-standardized mortality rate; EAPC, estimated annual percentage change.

Among the five SDI regions, the ASIR in the high-middle SDI regions was the highest in both 1990 and 2021 (1.04 and 0.67, respectively). From 1990 to 2021, the ASIR in the low SDI and low-middle SDI regions showed an upward trend, with EAPCs of 0.34 and 0.72, respectively (Table 1 and Supplementary Figure S3a). In the five SDI regions, the middle SDI and low SDI regions had the largest incident cases in 2021. Meanwhile, as the SDI level increased, the proportion in children aged 5–15 years increased significantly, while the proportion in children aged 2–4 years decreased significantly (Table 1, Supplementary Figure S2a and Supplementary Figure S4b). The AAPC in the high-low SDI region was the highest (−1.61, 95% CI: −1.89 –−1.34). In addition, the most significant decreases in the ASIR were observed in all five SDI regions in the recent five years. From the high SDI level to the low SDI level, the APC were −7.09 (2019–2021), −11.26 (2019–2021), −9.02 (2019–2021), −2.63 (2017–2021), and −1.71 (2016–2021), respectively (Supplementary Table S2 and Figure 1b). The pattern of KC incidence varies across the world. Among 21 GBD regions, Western Sub-Saharan Africa had the largest incident cases of KC in 2021 (1682; 95% UI, 1050–2349); Southern Latin America had the highest ASIR in 2021 (1.03; 95% UI, 0.83–1.25). From 1990 to 2021, the Eastern Europe had the fastest decrease in the incidence of KC (EAPC, −1.36; 95% CI, −1.85 –−0.54) and the Southern Sub-Saharan Africa had the fastest increase in the incidence of KC (EAPC, 1.28; 95% CI, 0.8–1.77) (Table 1, Figures 2a,d). Among the 204 countries or territories, China and Nigeria had the largest incident cases in 2021 (1,597 and 1,032 respectively), while Monaco had the highest ASIR of 1.46. The ASIR increased in 108 countries and decreased in 96 countries (Supplementary Table S3, Figures 3a–c). The EAPCs of four countries were over 3 from 1990 to 2021, including Mongolia (7.10), Cape Verde (6.43), Zimbabwe (4.17), Botswana (3.93) (Supplementary Table S3 and Figure 3c).

### Death trend of kidney cancer in children

Over the past 30 years, the global number of KC-associated deaths decreased from 4136 (95% UI, 3292–5010) in 1990 to 3063 (95% UI, 2252–3898) in 2021. Similarly, the age-standardized mortality rate (ASMR) decreased from 0.24 (95% UI, 0.19–0.29) to 0.15 (95% UI, 0.11–0.19); the EAPC was −1.02 (95% CI, −1.18 –−0.87) (Table 1). The ASMR of KC also showed a faster decline trend in females (EAPC = −1.81) than in males (EAPC = −0.36) (Table 1 and Supplementary Figure S1b). The AAPCs of the ASMR for females and males were −2.28 and −0.81, respectively. The ASMR for females continuously decreased from 1990 to 2021, while the ASMR for males increased slightly from 2006 to 2016. However, in the recent two years, the decreases for both females and males were the most obvious (APC = −7.20 and −3.66, respectively) (Supplementary Table S1 and Supplementary Figure S5a). The number of KC-associated deaths was highest among children aged 2 to 4 years in both 1990 and 2021 (1641 and 1179, respectively). From 1990 to 2021, the mortality rate of KC in each age group of children under 5 years old was higher than that in children aged 5–9 years and 10–14 years. From 1990 to 2021, both death cases and mortality rates of KC showed similar temporal patterns of decline in different age groups (Table 1, Supplementary Figure S2c and Supplementary Figure S2d).

From 1990 to 2021, the ASMR in the low SDI region was consistently the highest (0.27 in 2021). Meanwhile, the ASMR in all regions showed a decreasing trend, with EAPCs of −1.69 (high SDI region), −3.11 (high-middle SDI region), −1.99 (middle SDI region), −0.12 (low-middle SDI region), and −0.38 (low SDI region), respectively (Table 1 and Supplementary Figure S3b). The results of the Joinpoint analysis were similar, with the ASMR in all regions showing a downward trend, most evidently from 2019 to 2021 (from 2016 to 2021 in low SDI region). Among them, the downward trend of ASMR was the most significant in high-middle SDI regions (AAPC = −3.73, 95% CI: −4.04 –−3.41) (Supplementary Table S2 and Supplementary Figure S5b). With the increase of the SDI, the proportion of deaths among children aged 5 to 15 increased significantly, while the proportion of deaths among children aged 2 to 4 decreased significantly (Supplementary Figure S4c and Supplementary Figure S4d). In terms of 21 GBD regions, Western Sub-Saharan Africa had the highest death cases (859) and ASMR (0.40) of KC in 2021, whereas High-income Asia Pacific had the lowest ASMR (0.04). From 1990 to 2021, while the ASMR of KC in children increased only in Oceania, Southern Sub-Saharan Africa, and Western Sub-Saharan Africa, the ASMR in East Asia decreased the fastest (EAPC: −3.49; 95% CI: −3.7 –−3.28) (Table 1, Figures 2b,d). Malawi had the highest ASMR of KC in 2021 (0.65; 95% UI, 0.22–1.22) (Supplementary Table S3 and Figure 3d). A total of 42 countries increased corresponding ASMR from 1990 to 2021. Mongolia had the highest EAPC of KC-associated ASMR (5.82), followed by Cape Verde (5.04) and Zimbabwe (4.23) (Supplementary Table S3 and Figure 3f).

### DALYs trend of kidney cancer in children

From 1990 to 2021, the global number of KC-associated DALYs decreased from 362,175 (95% UI, 287,018–438,939) to 268,049 (95% UI, 196,876–341,769). Similarly, the age-standardized DALYs rate (ASDR) decreased from 20.82 (95% UI, 16.5–25.24) to 13.32 (95% UI, 9.79–16.99); the EAPC was −1.01 (95% CI, −1.17 –−0.86) (Table 1). The rapid decline periods associated with ASDR of KC were consistent with ASMR in males and females (Table 1, Supplementary Table S1, Supplementary Figure S1c and Supplementary Figure S6a). The number of KC-associated DALYs was highest among children aged 2 to 4 years in 2021 (103,552). From 1990 to 2021, the age distribution pattern of the DALYs rate was similar to those of incidence and mortality. The downward trends in DALYs number and DALYs rates was observed across all age groups, which is similar to that of deaths (Table 1, Supplementary Figure S2e and Supplementary Figure S2f).

Among the five SDI regions, the distribution and temporal trends of KC-related DALYs were similar to those of deaths, and the decline in ASDR was the most rapid from 2019 to 2021. The ASDR in the low SDI region has always been the highest (23.6 in 2021). Meanwhile, the downward trend of ASDR in the high-middle SDI regions was the most obvious, with EAPCs of −3.08 and an AAPC of −2.58 (Table 1, Supplementary Table S2, Supplementary Figure S3c, and Supplementary Figure S6b). In terms of 21 GBD regions, Western Sub-Saharan Africa had the largest DALYs number of KC (75,476) and the highest ASDR (35.14) in 2021. From 1990 to 2021, Southern Sub-Saharan Africa had the fastest increase (EAPC = 0.70) and East Asia had the fastest decrease (EAPC = −3.48) in the ASDR (Table 1, Figures 2c,d). Malawi had the highest ASDR among 204 countries (57.15) (Supplementary Table S3 and Supplementary Figure S7b). A total of 43 countries increased their ASDRs from 1990 to 2021. Mongolia had the highest EAPC of KC-associated ASDR (5.81), and Sri Lanka had the lowest EAPC (−8.21) (Supplementary Table S3 and Supplementary Figure S7c).

### Relationship between SDI and ASRs of KC burden

**Figure 4:**
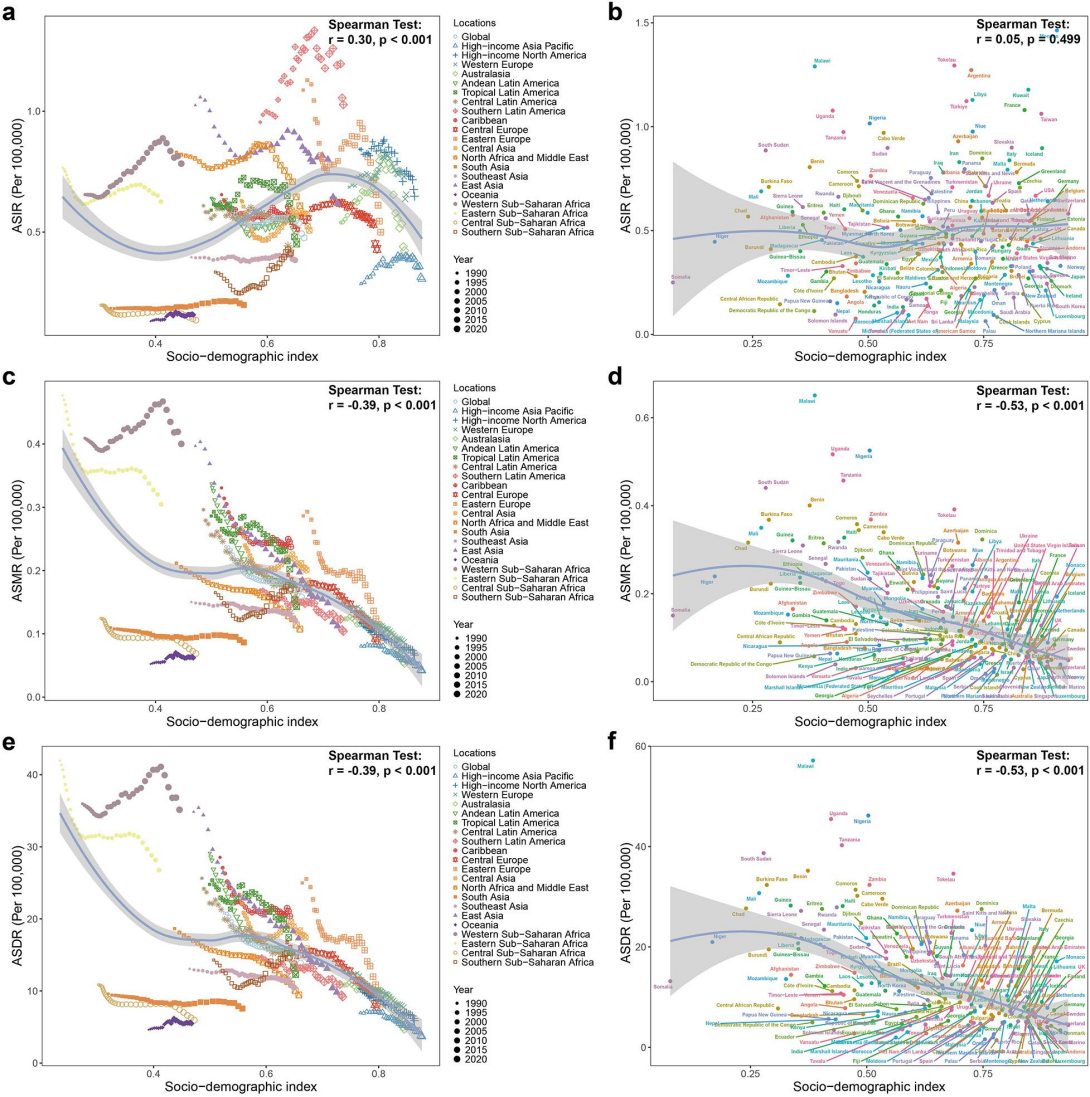
Correlation analysis between socio-demographic index and age-standardized rates of childhood kidney cancer burden. Correlations of socio-demographic index with age-standardized incidence **(a)**, mortality **(c)**, and DALYs **(e)** rates globally and across 21 regions, 1990−2021. Correlations of socio-demographic index with age-standardized incidence **(b)**, mortality **(d)**, and DALYs **(f)** rates in 204 countries and territories in 2021. ASIR, age-standardized incidence rate; ASMR, age-standardized mortality rate; ASDR, age-standardized disability-adjusted life years rate.

As can be seen from Figure 4a, in the world and among 21 GBD regions from 1990 to 2021, although the Spearman test yielded an r value of 0.30 (*p* < 0.001), there were obvious inflection points in the fitted curve, with the SDI values being 0.4 and 0.7 respectively. In 2021, there was no correlation between a country's SDI and the ASIR of kidney cancer in children (*p* = 0.499) (Figure 4b). For the global scope and the 21 GBD regions, there was a significant negative correlation between the SDI from 1990 to 2021 and its ASMR (r = −0.39, *p* < 0.001). In 2021, the SDI of 204 countries also showed a significant negative correlation with their ASMR (r = −0.53, *p* < 0.001) (Figures 4c,d). The relationship between the KC-related ASDR and the SDI is similar to that between the ASMR and the SDI (Figures 4e,f).

### Cross-countries health inequality

**Figure 5:**
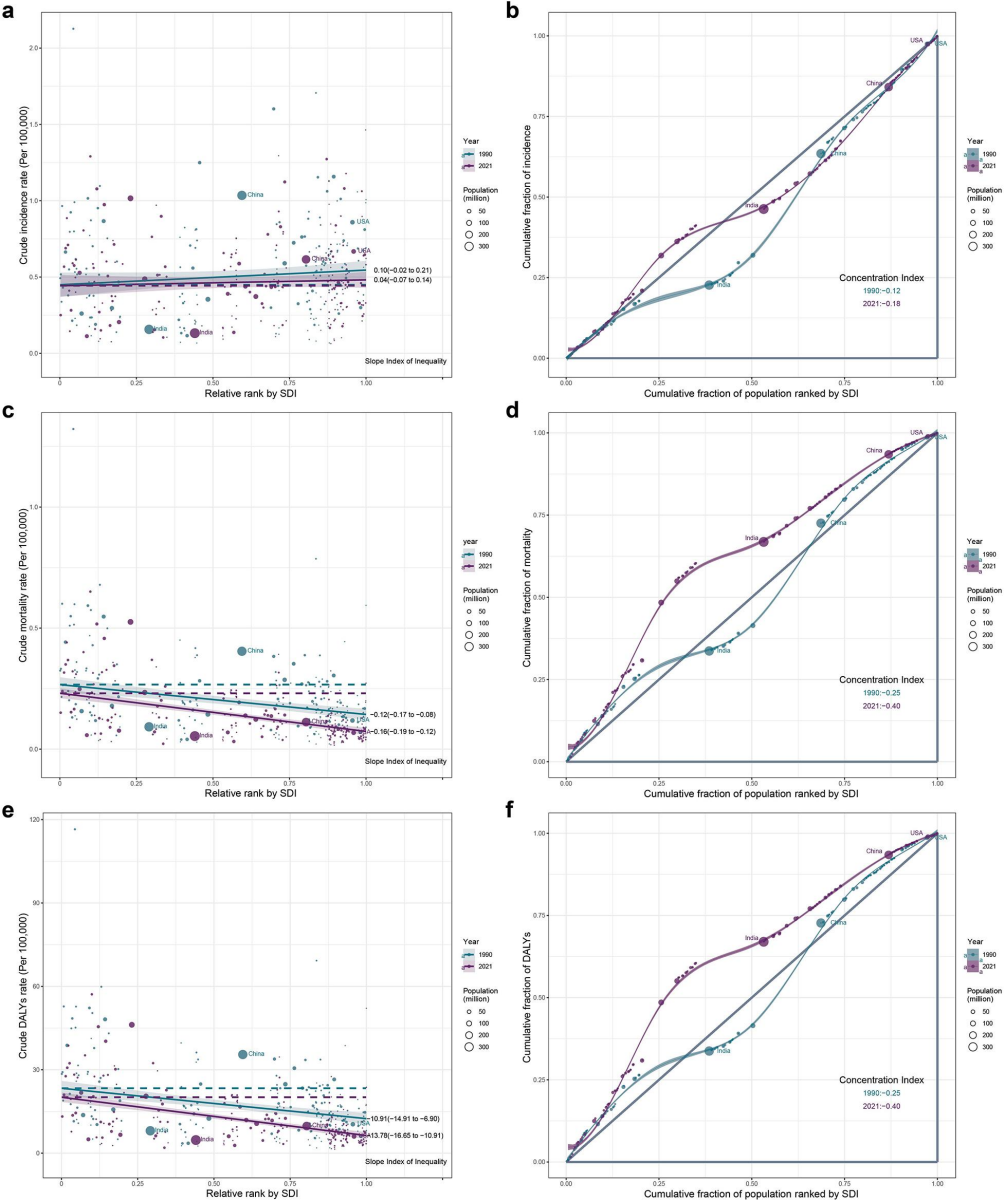
Health inequality analyses of childhood kidney cancer incidence, mortality, and DALYs in 1990 and 2021. Relative **(a)** and absolute **(b)** inequalities in kidney cancer incidence. Relative **(c)** and absolute **(d)** inequalities in kidney cancer mortality. Relative **(e)** and absolute **(f)** inequalities in DALYs of kidney cancer. DALYs, disability-adjusted life years; SDI, socio-demographic index.

Globally, no significant absolute inequality was observed in the incidence of renal cancer in children. However, there was relative SDI-related inequality, where countries with lower SDI disproportionately bore a higher incidence burden (Figures 5a,b). From 1990 to 2021, the concentration index of the incidence of KC in countries with lower SDI showed a deteriorating inequality, increasing from −0.12 to −0.18 (Figure 5b). Significant SDI-related absolute and SDI-related relative inequalities were observed in the deaths due to renal cancer in children, with countries having lower SDI disproportionately bearing a higher burden. From 1990 to 2021, the SII of KC deaths in countries with lower SDI showed an expanding inequality, rising from −0.12 (95% CI: −0.17 –−0.08) to −0.16 (95% CI: −0.19 –−0.12). The concentration index increased from −0.25 to −0.40 (Figures 5c,d). The health inequalities in terms of DALYs and deaths related to KC are similar. The SII increased from −10.91 (95% CI, −14.91 –−6.90) in 1990 to −13.78 (95% CI, −16.65 –−10.91), and the concentration index increased from −0.25 in 1990 to −0.40 in 2021 (Figures 5e,f).

### Global disease burden prediction to 2036

**Figure 6:**
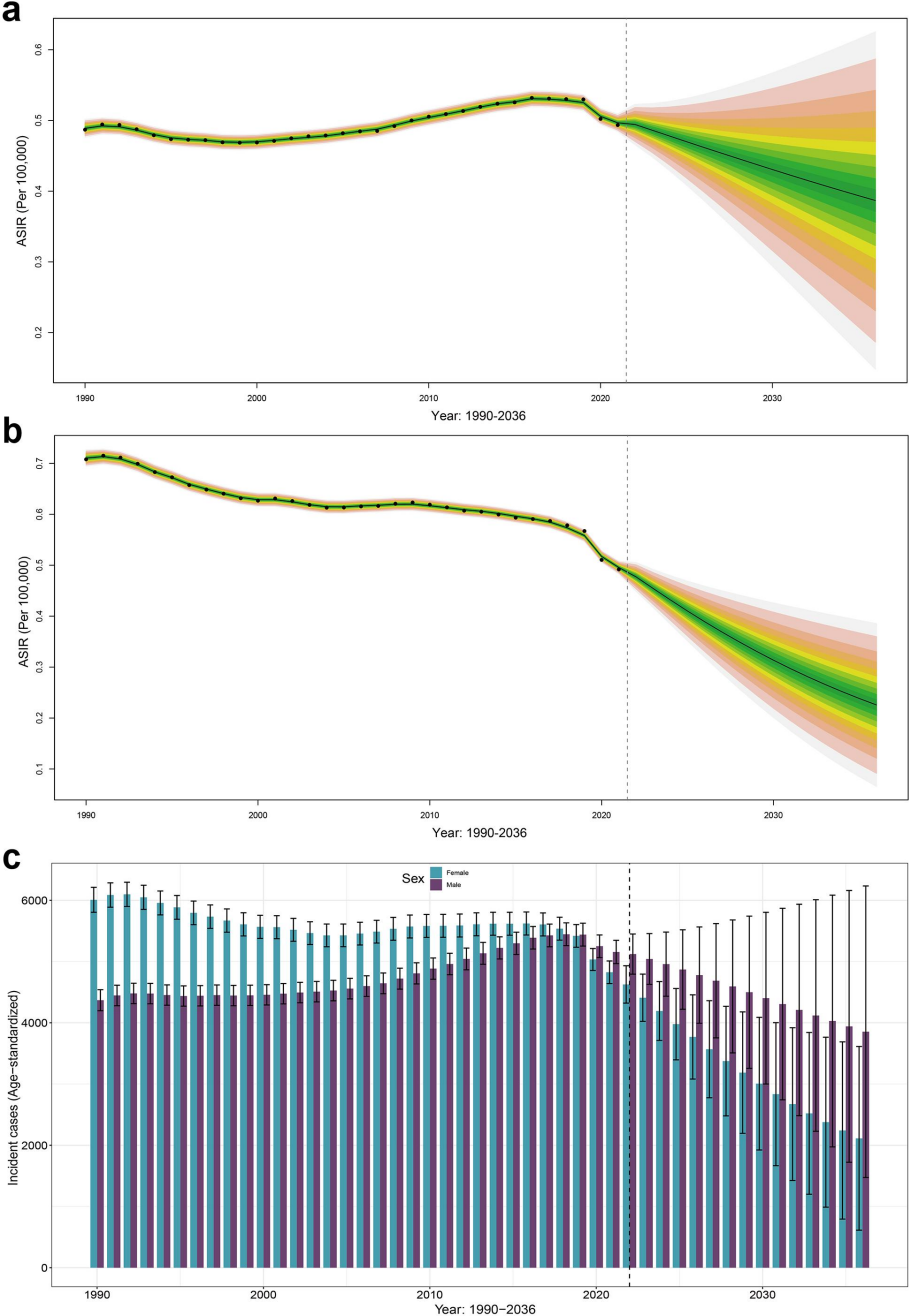
Projected age-standardized incidence rates of childhood kidney cancer in males **(a)** and females **(b)** globally and changes in the number of cases **(c)** for both from 2022 to 2036. ASIR, age-standardized incidence rate.

The prediction results show that the incident cases and death cases of KC in children worldwide will continue to decline in the next 15 years. By 2036, the incident cases will be 3,854 [95% credible interval (CrI), 1,473–6,234] and the death cases will be 1,539 (95% CrI, 517–2,562) among males, while the incident cases will reach 2,113 (95% CrI, 613–3,613) and the death cases will be 667 (95% CrI, 200–1,134) among females (Supplementary Table S4, Figure 6c and Supplementary Figure S8c). Meanwhile, the ASIR and the ASMR will show a continuous downward trend. By 2036, the ASIR and ASMR of KC in male children will be 0.39 (95% CrI, 0.15–0.63) and 0.15 (95% CrI, 0.05–0.26) respectively, while for female children, the ASIR and ASMR will be 0.23 (95% CrI, 0.07–0.39) and 0.07 (95% CrI, 0.02–0.12) respectively (Supplementary Table S4, Figures 6a,b, Supplementary Figure S8a and Supplementary Figure S8b).

## Discussion

To our knowledge, this is the first study utilizing the GBD dataset to delineate the trends in incidence, mortality, and DALYs of childhood KC globally from 1990 to 2021. While our findings quantify the combined disease burden of multiple pediatric renal tumors, we center our analysis on WT, as this tumor constitutes more than 90% of childhood KCs in patients younger than 20 years of age (8). The global burden of KC has been declining, with a notable acceleration since 2019. The decline in burden is more pronounced in females, whose ASIR approached that of males in 2021, while their ASMR and ASDR were lower. Age-specific incidence, mortality, and DALY rates were higher in children aged 0–4 years than in those aged 5–9 and 10–14 years, whereas overall mortality and DALY burdens across all ages declined over time. Substantial disparities in burden exist across regions and countries, with Western Sub-Saharan Africa recording the highest ASMR and ASDR, and Malawi standing out among nations. Notably, Mongolia warrants attention due to its most significant increases in incidence, mortality, and DALYs. While ASIR increased in low and low-middle SDI regions, ASMR and ASDR declined significantly across all SDI regions, most notably in middle-high SDI regions. SDI showed a negative correlation with ASMR and ASDR, and the burden of childhood KC was highly concentrated in lower-SDI countries. Encouragingly, the incidence and mortality of childhood KC are projected to continue declining over the next 15 years. The comprehensive analysis and projection of pediatric KC epidemiological trends and their association with SDI may assist policymakers and public health practitioners in formulating targeted future prevention and management strategies.

Globally, childhood KC accounted for 9,576 incident cases, 3,063 deaths, and 268,049 DALYs in 2021, with significant declines in ASIR, ASMR, and ASDR, consistent with previous studies (15). Due to the rarity of pediatric KC, previous epidemiological data have been limited, lacking trends analysis, which hampers our understanding of the disease patterns in many countries, particularly in underdeveloped countries (3–5, 16, 31, 32). To achieve comprehensive global health coverage, we present trends of pediatric KC across 204 countries or territories, offering valuable insights to promote health equity. Our findings indicate a declining global burden of childhood KC, with increasing incidence primarily observed among males and the 10–14 age group, predominantly concentrated in low and low-middle SDI regions, notably Southern Sub-Saharan Africa and Western Sub-Saharan Africa. The disparity in ASIR trends between male and female children globally is perplexing, given that WT, which constitutes the vast majority of pediatric KC cases, lacks identified external risk factors, exhibits significant genetic predisposition, and is typically more prevalent in females (10). Given the ongoing global decline in fertility rates and the predominant occurrence of WT in the 2–5 age group, the ASIR calculated for the 0–14 age range may be influenced by differing age distributions between male and female children (33). Additionally, the rising incidence of other non-WT renal cancers, such as renal clear cell sarcoma, which is more prevalent in males, and renal cell carcinoma, which predominantly affects the 9–15 age group, may also contribute to the observed trends (34). Our study's identification of an increasing ASIR for KC in the 10–14 age group underscores this trend, highlighting a demographic often overlooked by healthcare systems that warrants heightened attention (14). Simultaneously, the highest incidence, mortality, and DALY rates of KC were observed among children aged 0–4 years, emphasizing the critical importance of precise preventive strategies for neonatal KC.

This study also reveals a consistent global decline in mortality and DALY rates associated with childhood KC, attributable to significant advancements in multidisciplinary treatment approaches combining surgery, chemotherapy, and radiotherapy for WT. Over recent decades, these improvements have elevated the overall 5-year survival rate to over 90% (35, 36). However, continuous improvements in the treatment and management of pediatric KC remain imperative. Non-WT malignant renal tumors often exhibit poorer prognoses, with 5-year survival rates estimated at 87% for renal cell carcinoma, 68% for clear cell sarcoma of the kidney, and 23% for malignant rhabdoid tumor of the kidney (35, 37). From a global perspective, WT survival rates in most low-income countries remain significantly lower than those in high-income countries (90%), falling below 50% (38–40). Furthermore, WT treatment comes at a cost, as exposure to radiotherapy and anthracyclines is often unavoidable, with nearly 25% of survivors reporting severe chronic health conditions (11, 36). Further clinical trials incorporating additional risk factors such as tumor volume, response to chemotherapy, and loss of heterozygosity on chromosomes 1p and 16q are essential to refine therapeutic strategies and benefit more patient subgroups. Academic collaboration between the Children's Oncology Group (COG) and the International Society of Paediatric Oncology (SIOP), alongside international cooperation, is indispensable (36). The recent launch of initiatives such as the Global Initiative for Childhood Cancer (GICC) and The Collaborative Wilms Tumor Africa Project may have contributed to the decline in the burden of KC over the past five years, particularly since 2019 (38, 41).

The global burden of childhood KC is unevenly distributed across regions and nations, with Southern Latin America exhibiting the highest ASIR and Southern Sub-Saharan Africa along with Western Sub-Saharan Africa showing the highest ASMR. At the national level, Monaco has the highest ASIR, while Malawi records the highest ASMR. Countries with rising ASIR and ASMR are predominantly concentrated in Africa. Mongolia warrants particular attention due to its most significant increase in burden. Data indicate a strong correlation between WT incidence and ethnicity, with the highest rates observed among Black populations and the lowest among East Asian populations (39, 42). Notably, Black children born in Sub-Saharan Africa exhibit particularly high WT incidence rates. The significant genetic predisposition to WT is further supported by the higher frequency of congenital anomalies associated with WT in Black populations (42). Additionally, the scarcity of medical resources in Sub-Saharan Africa likely leads to an underestimation of incidence rates. Improvements in healthcare infrastructure and the implementation of international collaborative initiatives may enhance our understanding of the region's KC incidence burden (39). Controllable risk factors for childhood KC remain poorly defined. Some studies suggest that deficiencies in folic acid or vitamin supplements, maternal pesticide exposure, high birth weight, and preterm birth may increase the risk of WT, though robust evidence is lacking (43–45). Similarly, risk factors for other types of pediatric KC are also unclear, necessitating further research.

Additionally, we found no significant association between the SDI and the ASIR of childhood KC in 2021, indicating that its incidence is independent of economic status, consistent with other studies (10). However, the results revealed a negative correlation between SDI and the mortality and DALYs rates of childhood KC, with a disproportionate concentration of deaths and DALYs in low-SDI countries. This health inequity is strikingly evident. A review of WT survival data across 75 countries globally found that survival rates in low-income and many middle-income countries are significantly lower than in high-income nations. The survival rates for WT in Sub-Saharan Africa are concerning, with Sudan reporting a rate of only 11%, while Malawi has the highest rate at 46% (38, 46). Factors such as delayed diagnosis, malnutrition, drug toxicity, inadequate healthcare resources, and lack of education contribute to the high mortality and DALYs rates in low-SDI countries (39). Moreover, our findings indicate that health disparities have become more pronounced in 2021 compared to 1990, underscoring the urgent need for increased medical assistance in low-SDI regions, particularly within Sub-Saharan Africa. In low-income countries with limited medical resources, up to 50%–60% of cancer cases result in treatment abandonment, severely impacting patient survival (39, 47). This issue is not solely related to economic conditions but also stems from a lack of awareness about cancer (38). Therefore, enhancing public health education is a crucial measure to ensure patient survival and should not be overlooked. Our predictive results indicate that the incidence and mortality of childhood KC in both males and females worldwide will continue to decline over the next 15 years, which is highly encouraging. Compared to developed nations, the elimination of the disease burden in less developed regions remains a critical focus for achieving comprehensive health coverage goals in the future.

Although this study fills a gap in the analysis of global trends in childhood KC burden using the GBD 2021 dataset, several limitations should be noted. First, the analysis relies on the GBD database, whose estimates are model-derived and subject to the quality and availability of national cancer registry data. Epidemiological data from regions with limited medical resources are often sparse and of poor quality, leading to wide uncertainty intervals and affecting the precision of estimates. Moreover, variations in cancer registry completeness and coding practices across countries may introduce systematic bias, particularly in resource-limited settings (18). Second, the GBD 2021 study does not provide statistics on KC subtypes, leading us to focus primarily on WT in our analysis. Third, the GBD database lacks clinical data on unilateral versus bilateral disease, tumor staging, and other clinical features, limiting our ability to explore the clinical characteristics of childhood KC in greater depth. Finally, the restricted public accessibility of the GBD 2023 dataset prevented us from using the latest data release. Future research should leverage GBD 2023 to perform sensitivity analyses and confirm the robustness of our findings.

## Conclusion

Over the past three decades, global healthcare efforts have significantly reduced the burden of childhood KC, yet the rising incidence in low and low-middle SDI regions remains a critical concern. The widening health inequities associated with SDI highlight the urgent need for enhanced access to KC treatment and management resources in low-income countries to address the disproportionate concentration of the disease burden in lower SDI countries. This comprehensive epidemiological study on childhood KC provides valuable evidence to inform policy makers and public health practitioners in developing prevention and management strategies.

## Data Availability

Publicly available datasets were analyzed in this study. This data can be found here: https://vizhub.healthdata.org/gbd-results/.
