# Supplementary material for: Global burden and temporal trends of kidney cancer among children from 1990 to 2021: an analysis with projections to 2036
Source: Front Pediatr. 2026 Jul 9;14:1882218. doi: 10.3389/fped.2026.1882218 (PMC13391957; doi:10.3389/fped.2026.1882218)
Supplement: Supplementary file 1 [file Datasheet1.pdf]

**Checklist of information that should be included in new reports of global health estimates**

| Item # | Checklist item | Reported on page # |
|---|---|---|
| **Objectives and funding** | | |
| 1 | Define the indicator(s), populations (including age, sex, and geographic entities), and time period(s) for which estimates were made. | 4 |
| 2 | List the funding sources for the work. | 14 |
| **Data Inputs** | | |
| *For all data inputs from multiple sources that are synthesized as part of the study:* | | |
| 3 | Describe how the data were identified and how the data were accessed. | 4 |
| 4 | Specify the inclusion and exclusion criteria. Identify all ad_hoc exclusions. | 4 |
| 5 | Provide information on all included data sources and their main characteristics. For each data source used, report reference information or contact name/institution, population represented, data collection method, year(s) of data collection, sex and age range, diagnostic criteria or measurement method, and sample size, as relevant. | Not applicable |
| 6 | Identify and describe any categories of input data that have potentially important biases (e.g., based on characteristics listed in item 5). | Not applicable |
| *For data inputs that contribute to the analysis but were not synthesized as part of the study:* | | |
| 7 | Describe and give sources for any other data inputs. | 4 |
| *For all data inputs:* | | |
| 8 | Provide all data inputs in a file format from which data can be efficiently extracted (e.g., a spreadsheet rather than a PDF), including all relevant meta_data listed in item 5. For any data inputs that cannot be shared because of ethical or legal reasons, such as third_party ownership, provide a contact name or the name of the institution that retains the right to the data. | 4 |
| **Data analysis** | | |
| 9 | Provide a conceptual overview of the data analysis method. A diagram may be helpful. | 5-6 |
| 10 | Provide a detailed description of all steps of the analysis, including mathematical formulae. This description should cover, as relevant, data cleaning, data pre_processing, data adjustments and weighting of data sources, and mathematical or statistical model(s). | 5-6 |
| 11 | Describe how candidate models were evaluated and how the final model(s) were selected. | 5-6 |

| | | |
|---|---|---|
| **12** | Provide the results of an evaluation of model performance, if done, as well as the results of any relevant sensitivity analysis. | Not applicable |
| **13** | Describe methods for calculating uncertainty of the estimates. State which sources of uncertainty were, and were not, accounted for in the uncertainty analysis. | 5-6 |
| **14** | State how analytic or statistical source code used to generate estimates can be accessed. | 5-6 |
| **Results and Discussion** | | |
| **15** | Provide published estimates in a file format from which data can be efficiently extracted. | 6-10 |
| **16** | Report a quantitative measure of the uncertainty of the estimates (e.g. uncertainty intervals). | 6-10 |
| **17** | Interpret results in light of existing evidence. If updating a previous set of estimates, describe the reasons for changes in estimates. | Not applicable |
| **18** | Discuss limitations of the estimates. Include a discussion of any modelling assumptions or data limitations that affect interpretation of the estimates. | 13 |

*This checklist should be used in conjunction with the GATHER statement and Explanation and Elaboration document, found on gather_statement.org*

# *Supplementary Material*

## Supplementary Figures

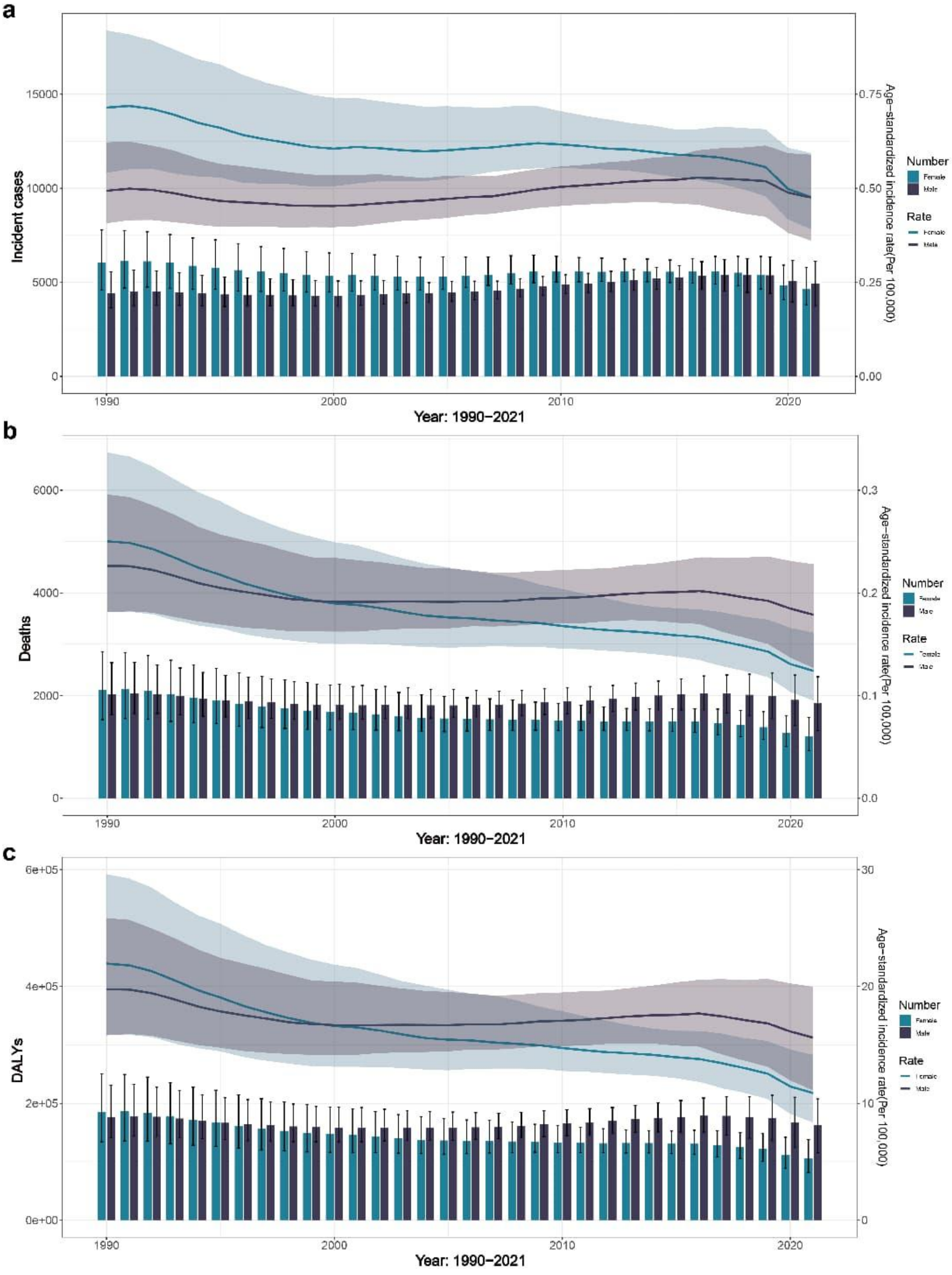


**Fig S1. Changes in the number and age-standardized rates of the global burden of childhood kidney cancer in males and females, 1990-2021.** (a) The number of incidence and age-standardized incidence rate; (b) The number of mortality and age-standardized mortality rate; (b) The number of DALYs and age-standardized DALYs rate. DALYs, disability-adjusted life years.

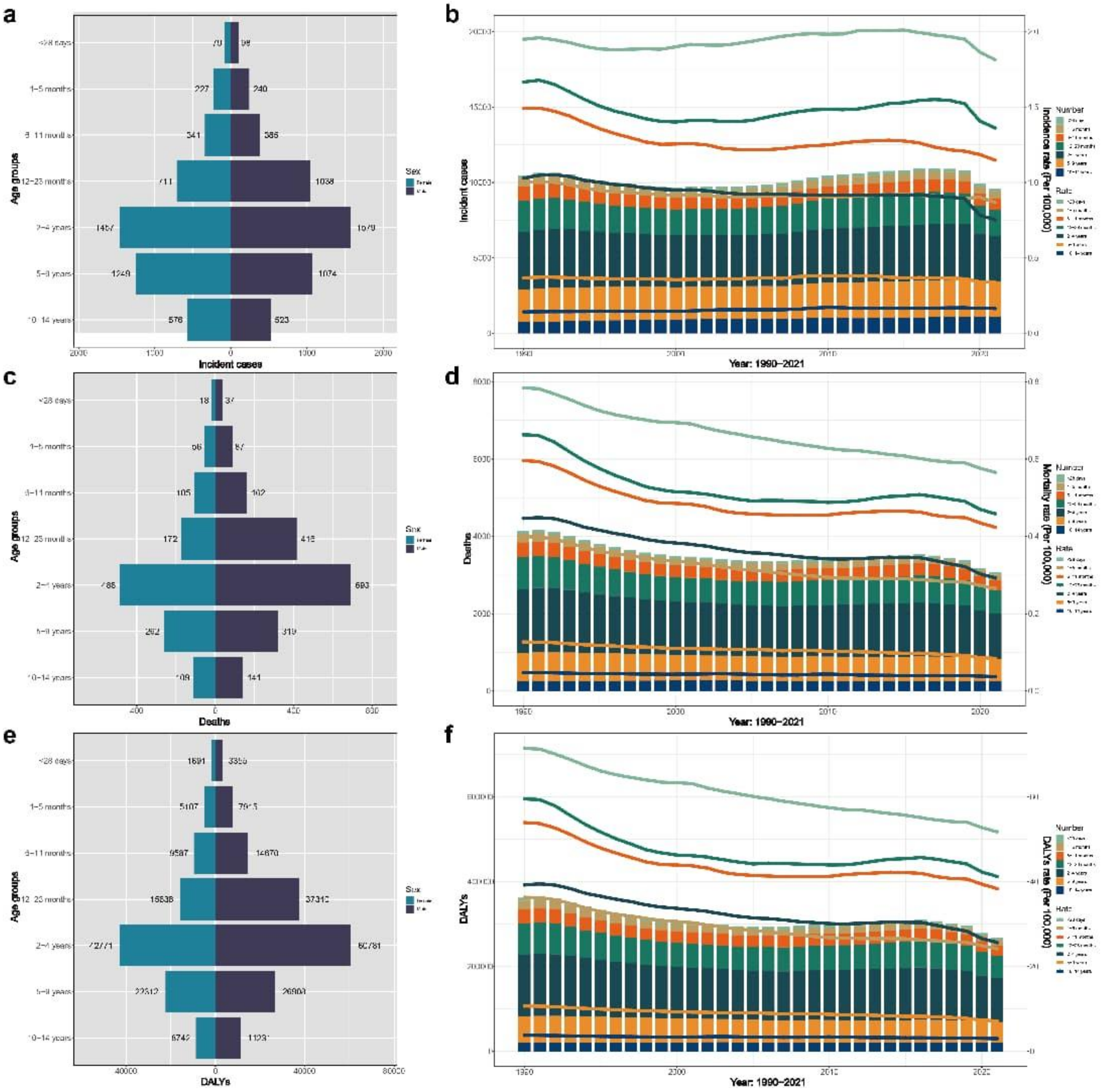


**Fig 3. Trends in the global burden of childhood kidney cancer across different age groups from 1990 to 2021 and sex distribution in 2021.** (a) Sex distribution in the incident cases in 2021. (b) Trends in incidence from 1990 to 2021. (c) Sex distribution in the death cases in 2021. (d) Trends in mortality from 1990 to 2021. (e) Sex distribution in the number of DALYs in 2021. (f) Trends in DALYs from 1990 to 2021. DALYS, disability-adjusted life years.

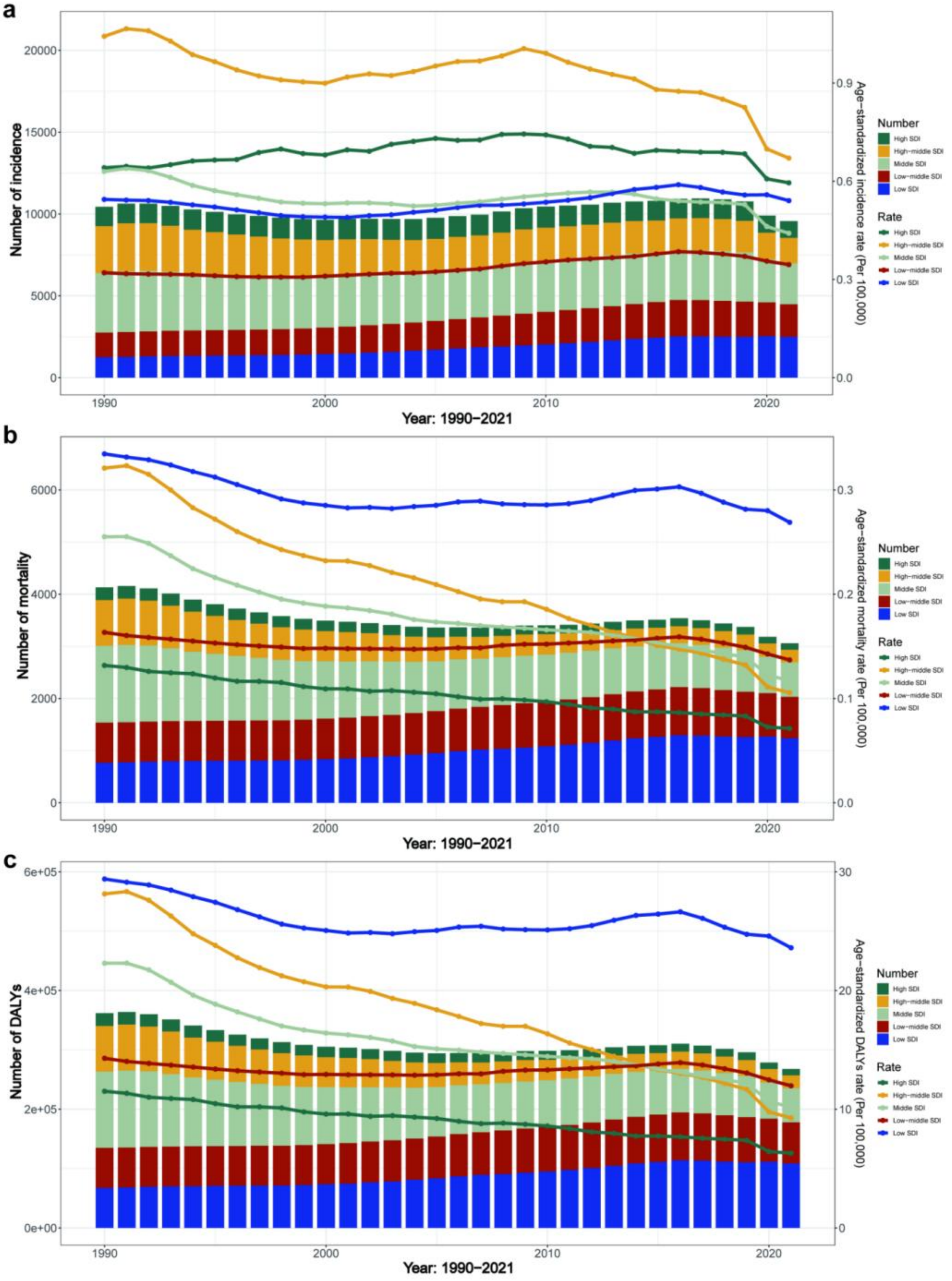


**Fig S3. Trends in the number and age-standardized rates of childhood kidney cancer burden across 5 SDI regions, 1990-2021.** (a) number of incident cases and age-standardized incidence rates; (b) number of deaths and age-standardized mortality rates; (c) number of DALYs and age-standardized DALYs rates. DALYs, disability-adjusted life years; SDI, socio-demographic index.

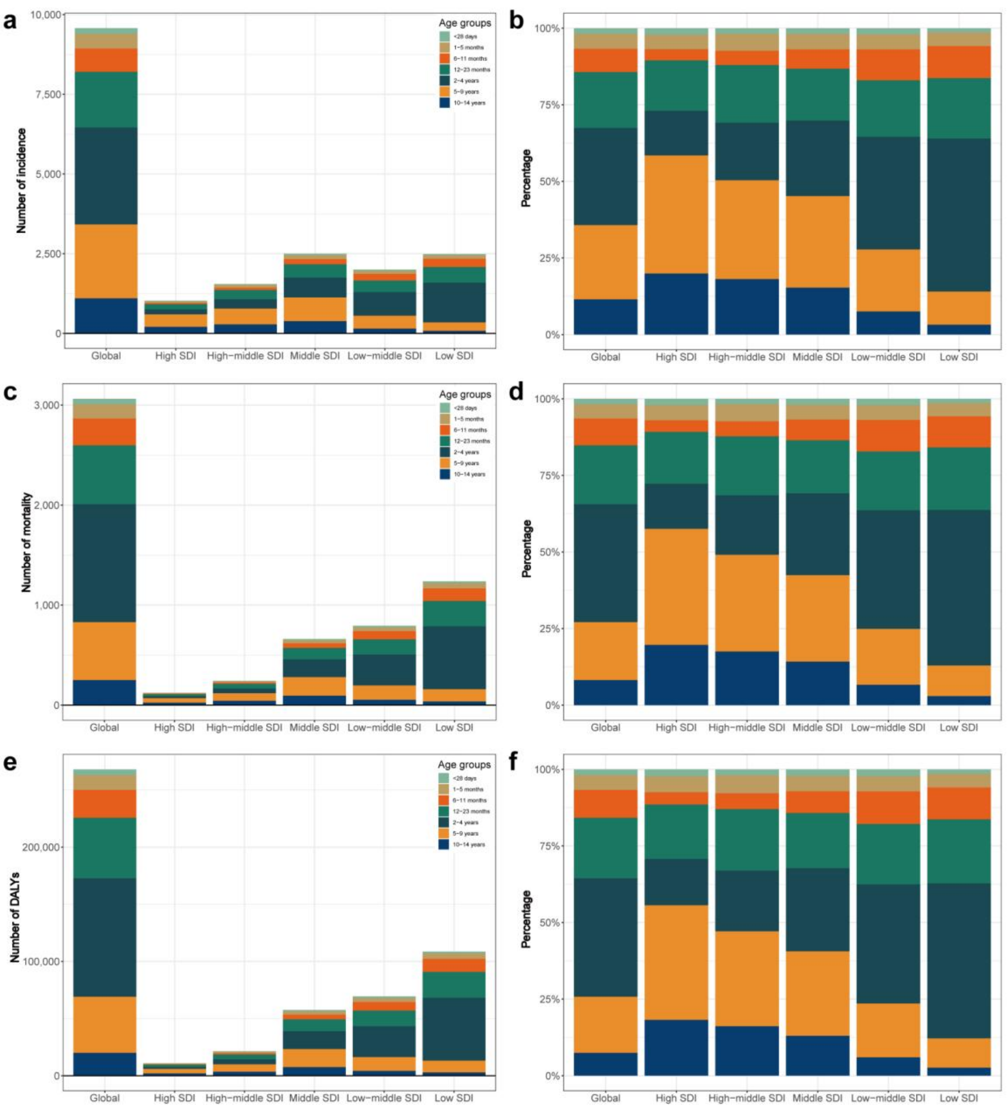


**Fig S4. Number and proportion of childhood kidney cancer burden across 5 SDI regions in 2021.** Number (a) and proportion (b) of incident cases of kidney cancer. Number (c) and proportion (d) of deaths due to kidney cancer. Number (e) and proportion (f) of DALYs due to kidney cancer. DALYs, disability-adjusted life years; SDI, socio-demographic index.

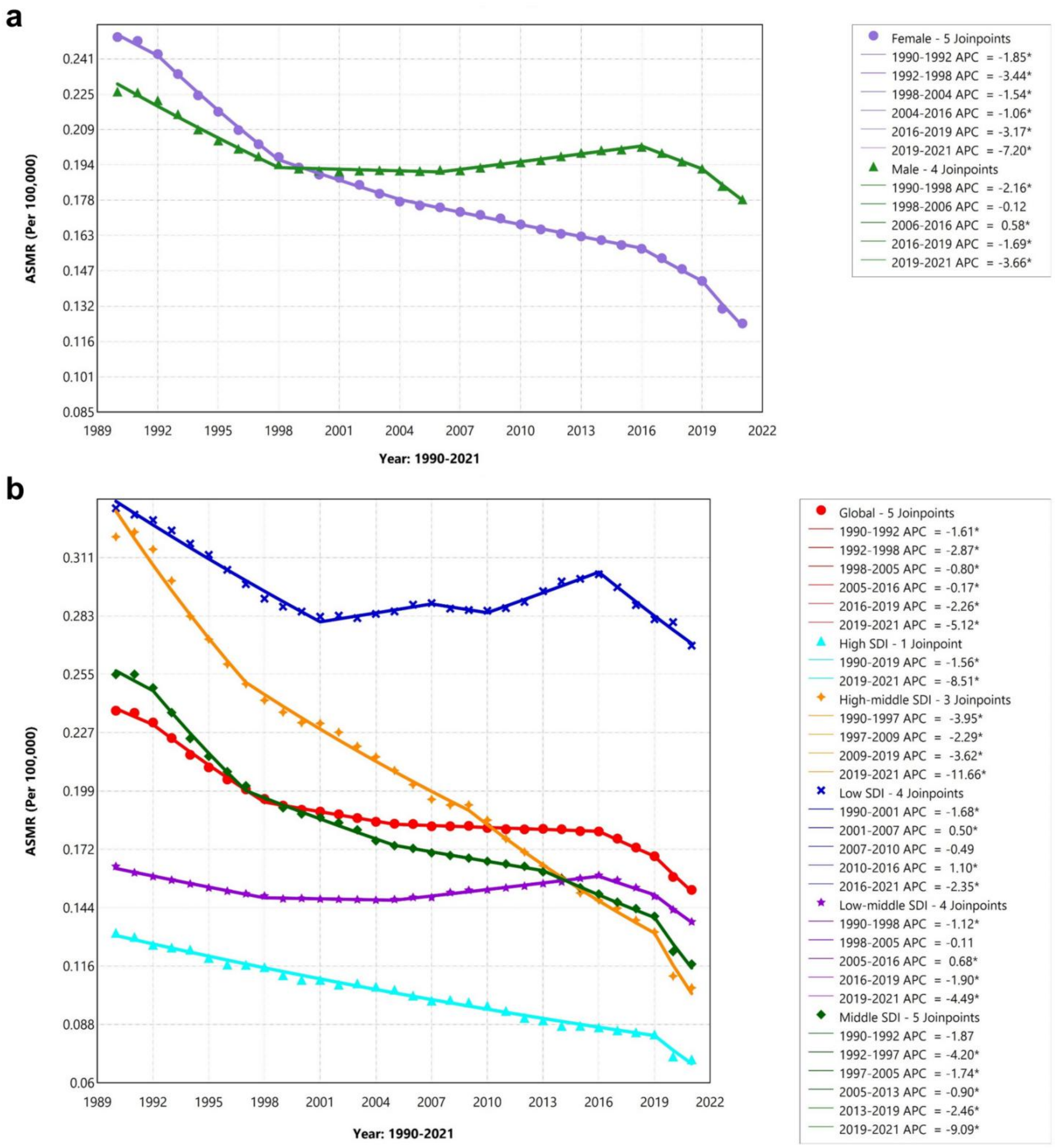


**Fig S5. Joinpoint regression analysis of age-standardized mortality rates of childhood kidney cancer by sex(a) and across different SDI regions(b), 1990~2021.** APC, annual percentage change; ASMR, age-standardized mortality rate; SDI, socio-demographic index.

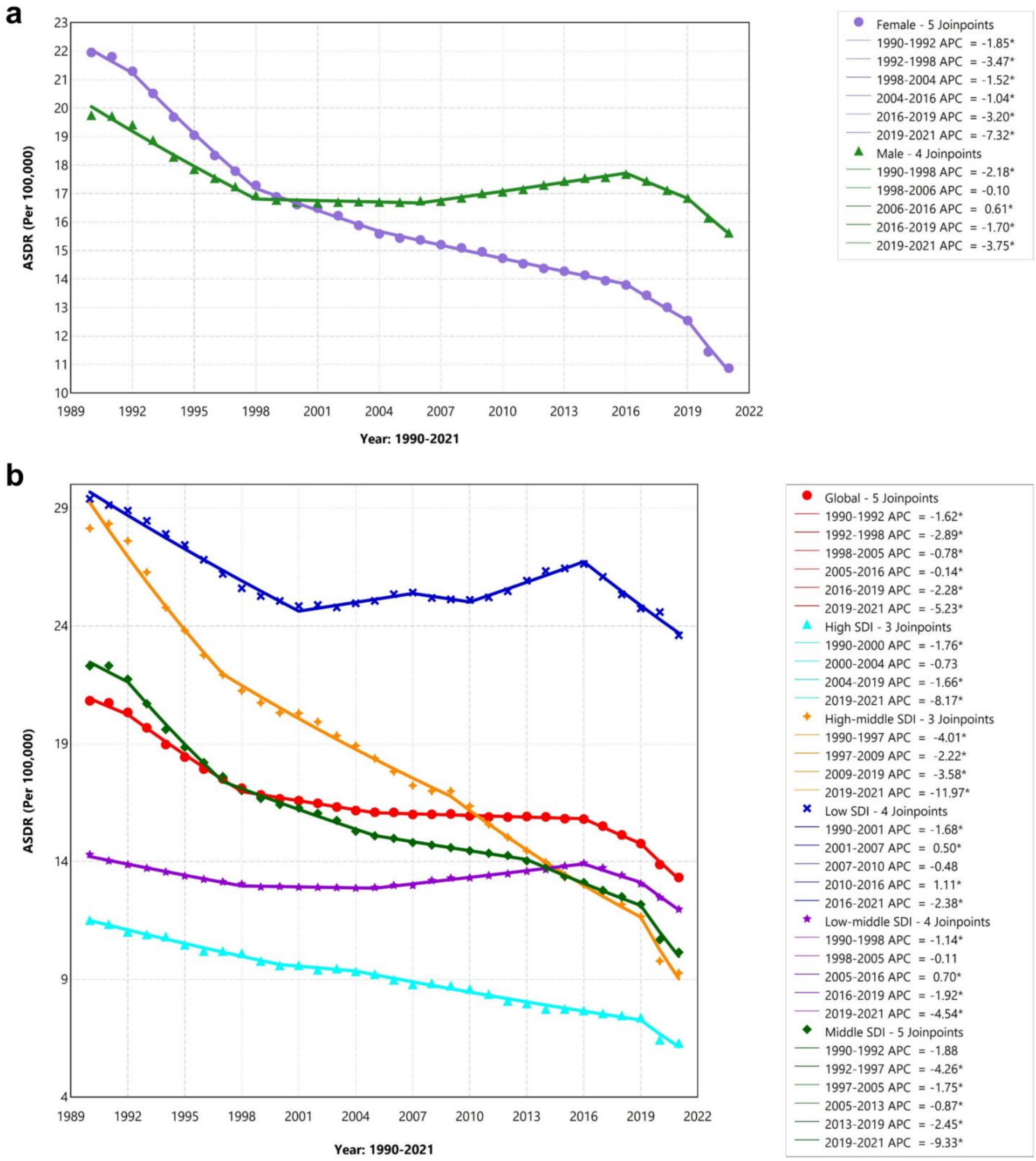


**Fig S6. Joinpoint regression analysis of age-standardized DALYs rates of childhood kidney cancer by sex(a) and across different SDI regions(b), 1990~2021.** APC, annual percentage change; ASDR, age-standardized DALYs rate; DALYs, disability-adjusted life years; SDI, socio-demographic index.

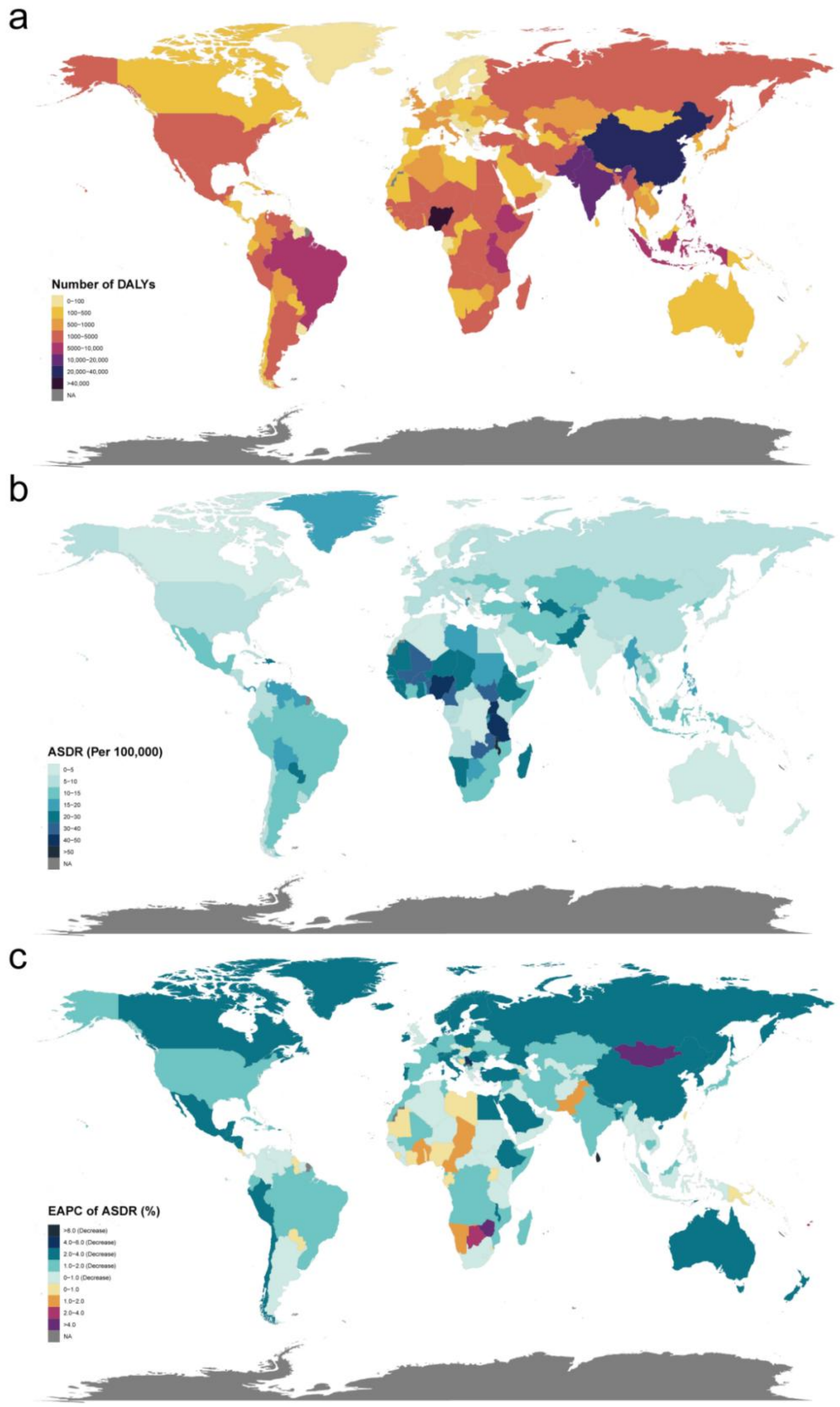


**Fig S7. DALYs and their trends of childhood kidney cancer in 204 countries and territories worldwide, 2021.** (a) The number of DALYs for kidney cancer. (b) The age-standardized DALYs of kidney cancer. (c) The EAPC of ASDR. ASDR, age-standardized DALYs rate; EAPC, estimated annual percentage change; DALYs, disability-adjusted life years.

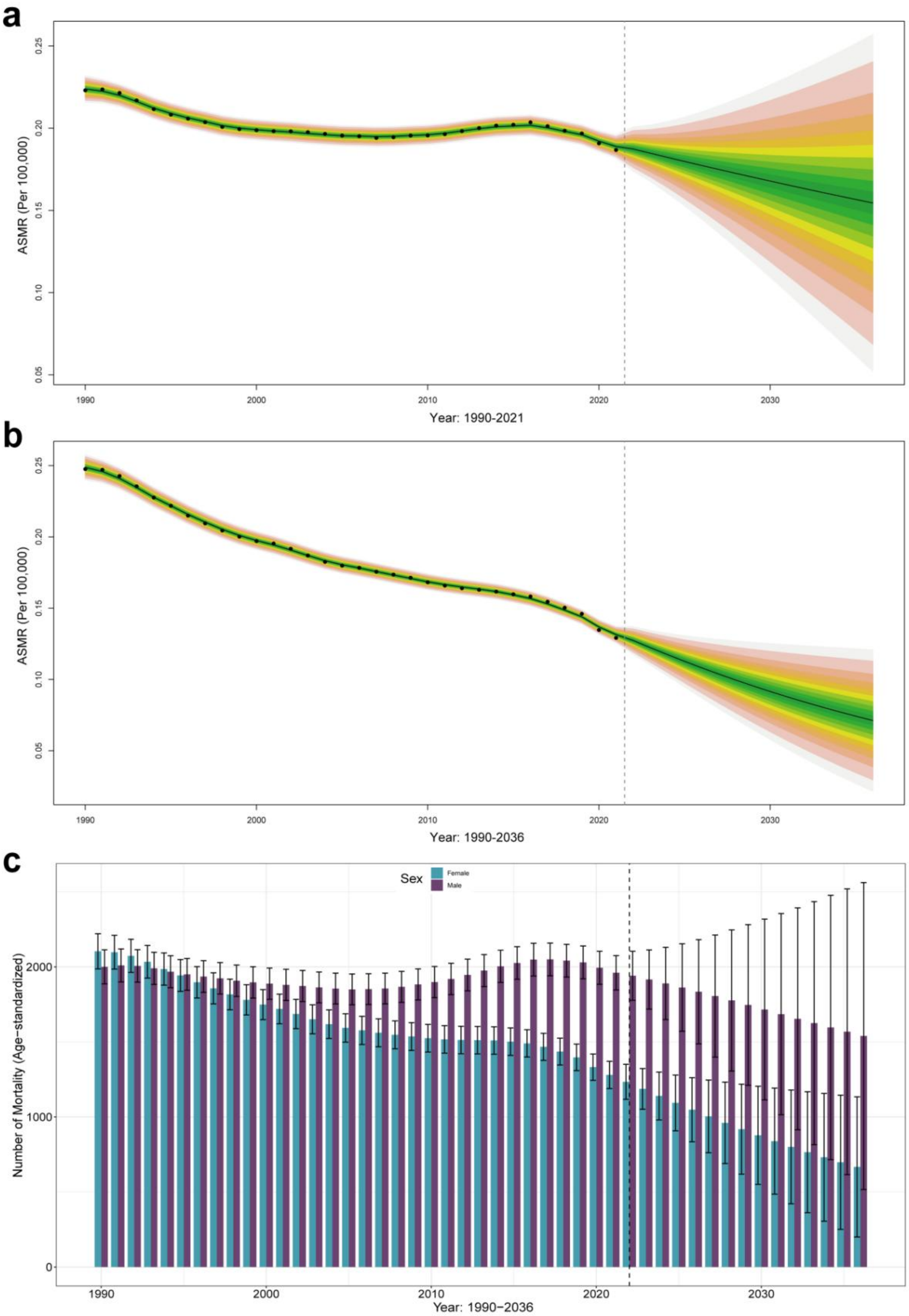


**Fig S8. Projected age-standardized mortality rates of childhood kidney cancer in males (a) and females (b) globally and changes in the number of cases (c) for both from 2022 to 2036.** ASMR, age-standardized mortality rate

# Supplementary Material

## Supplementary Tables

**Table S1: The Joinpoint regression analyses for age-standardized rates of kidney cancer burden in children from 1990 to 2021 in males and females**

| Sex | ASIR (Per 100,000) | | | | | ASMR (Per 100,000) | | | | | ASDR (Per 100,000) | | | | |
|---|---|---|---|---|---|---|---|---|---|---|---|---|---|---|---|
| | Period | APC, % (95% CI) | P-values | AAPC, % (95% CI) | P-values | Period | APC, % (95% CI) | P-values | AAPC, % (95% CI) | P-values | Period | APC, % (95% CI) | P-values | AAPC, % (95% CI) | P-values |
| Female | 1990-1999 | -2.00 (-2.29,-1.71) | <0.001 | -1.37 (-1.58,-1.15) | <0.001 | 1990-1992 | -1.85 (-3.26,-0.41) | 0.015 | -2.28 (-2.43,-2.13) | <0.001 | 1990-1992 | -1.85 (-3.30,-0.38) | 0.017 | -2.28 (-2.44,-2.13) | <0.001 |
| | 1999-2005 | -0.27 (-0.82,0.29) | 0.326 | | | 1992-1998 | -3.44 (-3.73,-3.15) | <0.001 | | | 1992-1998 | -3.47 (-3.77,-3.17) | <0.001 | | |
| | 2005-2009 | 0.90 (-0.10,1.91) | 0.076 | | | 1998-2004 | -1.54 (-1.80,-1.28) | <0.001 | | | 1998-2004 | -1.52 (-1.78,-1.25) | <0.001 | | |
| | 2009-2018 | -0.81 (-0.99,-0.63) | <0.001 | | | 2004-2016 | -1.06 (-1.12,-1.00) | <0.001 | | | 2004-2016 | -1.04 (-1.10,-0.98) | <0.001 | | |
| | 2018-2021 | -6.19 (-7.39,-4.97) | <0.001 | | | 2016-2019 | -3.17 (-3.97,-2.36) | <0.001 | | | 2016-2019 | -3.20 (-4.02,-2.37) | <0.001 | | |

| | | | | | | | | | | | | | | | |
|---|---|---|---|---|---|---|---|---|---|---|---|---|---|---|---|
| | | | | | | 2019-2021 | -7.20 (-8.31,-6.09) | <0.001 | | | 2019-2021 | -7.32 (-8.45,-6.19) | <0.001 | | |
| Male | 1990-1999 | -1.19 (-1.38,-1.01) | <0.001 | -0.19 (-0.44,0.06) | 0.144 | 1990-1998 | -2.16 (-2.34,-1.99) | <0.001 | -0.81 (-0.99,-0.63) | <0.001 | 1990-1998 | -2.18 (-2.36,-2.01) | <0.001 | -0.81 (-0.99,-0.63) | <0.001 |
| | 1999-2007 | 0.82 (0.63,1.02) | <0.001 | | | 1998-2006 | -0.12 (-0.29,0.04) | 0.126 | | | 1998-2006 | -0.10 (-0.27,0.06) | 0.208 | | |
| | 2007-2010 | 1.70 (0.54,2.87) | 0.007 | | | 2006-2016 | 0.58 (0.49,0.67) | <0.001 | | | 2006-2016 | 0.61 (0.52,0.70) | <0.001 | | |
| | 2010-2016 | 0.77 (0.49,1.05) | <0.001 | | | 2016-2019 | -1.69 (-3.02,-0.33) | 0.018 | | | 2016-2019 | -1.70 (-3.05,-0.32) | 0.019 | | |
| | 2016-2019 | -0.63 (-2.37,1.15) | 0.462 | | | 2019-2021 | -3.66 (-5.50,-1.78) | 0.001 | | | 2019-2021 | -3.75 (-5.61,-1.86) | 0.001 | | |
| | 2019-2021 | -4.58 (-6.97,-2.14) | 0.001 | | | | | | | | | | | | |

ASIR, age-standardized incidence rate; APC, annual percent change; CI, confidence interval; AAPC, average annual percent change; ASMR, age-standardized mortality rate; ASDR, age-standardized disability-adjusted life years rate.

**Table S2: The Joinpoint regression analyses for age-standardized rates of kidney cancer burden in children from 1990 to 2021 globally and in SDI regions**

| Locations | ASIR (Per 100,000) | | | | | ASMR (Per 100,000) | | | | | ASDR (Per 100,000) | | | | |
|---|---|---|---|---|---|---|---|---|---|---|---|---|---|---|---|
| | Period | APC, % (95% CI) | P-values | AAPC, % (95% CI) | P-values | Period | APC, % (95% CI) | P-values | AAPC, % (95% CI) | P-values | Period | APC, % (95% CI) | P-values | AAPC, % (95% CI) | P-values |
| Global | 1990-1999 | -1.66 (-1.87,-1.45) | <0.001 | -0.81 (-0.98,-0.64) | <0.001 | 1990-1992 | -1.61 (-2.68,-0.54) | 0.006 | -1.46 (-1.60,-1.32) | <0.001 | 1990-1992 | -1.62 (-2.68,-0.54) | 0.006 | -1.46 (-1.60,-1.32) | <0.001 |
| | 1999-2005 | 0.21 (-0.17,0.59) | 0.256 | | | 1992-1998 | -2.87 (-3.08,-2.65) | <0.001 | | | 1992-1998 | -2.89 (-3.11,-2.68) | <0.001 | | |
| | 2005-2010 | 0.98 (0.55,1.42) | <0.001 | | | 1998-2005 | -0.80 (-0.92,-0.67) | <0.001 | | | 1998-2005 | -0.78 (-0.90,-0.65) | <0.001 | | |
| | 2010-2018 | -0.15 (-0.35,0.06) | 0.147 | | | 2005-2016 | -0.17 (-0.22,-0.12) | <0.001 | | | 2005-2016 | -0.14 (-0.20,-0.09) | <0.001 | | |
| | 2018-2021 | -4.87 (-6.13,-3.60) | <0.001 | | | 2016-2019 | -2.26 (-3.20,-1.30) | <0.001 | | | 2016-2019 | -2.28 (-3.22,-1.33) | <0.001 | | |
| | | | | | | 2019-2021 | -5.12 (-6.42,-3.80) | <0.001 | | | 2019-2021 | -5.23 (-6.52,-3.92) | <0.001 | | |
| High SDI | 1990-2008 | 0.84 (0.72,0.95) | <0.001 | -0.30 (-0.74,0.14) | 0.183 | 1990-2019 | -1.56 (-1.62,-1.50) | <0.001 | -2.03 (-2.46,-1.59) | <0.001 | 1990-2000 | -1.76 (-2.00,-1.52) | <0.001 | -2.01 (-2.39,-1.63) | <0.001 |

| | | | | | | | | | | | | | | | |
|---|---|---|---|---|---|---|---|---|---|---|---|---|---|---|---|
| | 2008-2019 | -0.86 (-1.19,-0.53) | <0.001 | | | 2019-2021 | -8.51 (-14.78,-1.77) | 0.016 | | | 2000-2004 | -0.73 (-2.06,0.62) | 0.272 | | |
| | 2019-2021 | -7.09 (-13.25,-0.49) | 0.037 | | | | | | | | 2004-2019 | -1.66 (-1.81,-1.51) | <0.001 | | |
| | | | | | | | | | | | 2019-2021 | -8.17 (-13.10,-2.96) | 0.004 | | |
| High-middle SDI | 1990-1999 | -2.11 (-2.48,-1.74) | <0.001 | -1.61 (-1.89,-1.34) | <0.001 | 1990-1997 | -3.95 (-4.61,-3.29) | <0.001 | -3.73 (-4.04,-3.41) | <0.001 | 1990-1997 | -4.01 (-4.64,-3.37) | <0.001 | -3.72 (-4.03,-3.41) | <0.001 |
| | 1999-2009 | 1.16 (0.89,1.43) | <0.001 | | | 1997-2009 | -2.29 (-2.53,-2.06) | <0.001 | | | 1997-2009 | -2.22 (-2.45,-1.99) | <0.001 | | |
| | 2009-2019 | -1.87 (-2.10,-1.65) | <0.001 | | | 2009-2019 | -3.62 (-3.89,-3.34) | <0.001 | | | 2009-2019 | -3.58 (-3.85,-3.32) | <0.001 | | |
| | 2019-2021 | -11.26 (-14.67,-7.71) | <0.001 | | | 2019-2021 | -11.66 (-15.50,-7.64) | <0.001 | | | 2019-2021 | -11.97 (-15.69,-8.09) | <0.001 | | |
| Middle SDI | 1990-1998 | -2.37 (-2.71,-2.03) | <0.001 | -1.29 (-1.58,-1.01) | <0.001 | 1990-1992 | -1.87 (-3.97,0.28) | 0.083 | -2.56 (-2.79,-2.34) | <0.001 | 1990-1992 | -1.88 (-3.99,0.28) | 0.083 | -2.58 (-2.81,-2.36) | <0.001 |

| | | | | | | | | | | | | | | | |
|---|---|---|---|---|---|---|---|---|---|---|---|---|---|---|---|
| | 1998-2005 | -0.25 (-0.59,0.10) | 0.15 | | | 1992-1997 | -4.20 (-4.74,-3.66) | <0.001 | | | 1992-1997 | -4.26 (-4.80,-3.72) | <0.001 | | |
| | 2005-2012 | 1.16 (0.84,1.47) | <0.001 | | | 1997-2005 | -1.74 (-1.91,-1.57) | <0.001 | | | 1997-2005 | -1.75 (-1.92,-1.58) | <0.001 | | |
| | 2012-2019 | -1.22 (-1.65,-0.78) | <0.001 | | | 2005-2013 | -0.90 (-1.06,-0.74) | <0.001 | | | 2005-2013 | -0.87 (-1.03,-0.71) | <0.001 | | |
| | 2019-2021 | -9.02 (-12.62,-5.29) | <0.001 | | | 2013-2019 | -2.46 (-2.83,-2.09) | <0.001 | | | 2013-2019 | -2.45 (-2.82,-2.09) | <0.001 | | |
| | | | | | | 2019-2021 | -9.09 (-11.34,-6.77) | <0.001 | | | 2019-2021 | -9.33 (-11.58,-7.03) | <0.001 | | |
| Low-middle SDI | 1990-1998 | -0.59 (-0.77,-0.41) | <0.001 | 0.26 (0.14,0.39) | <0.001 | 1990-1998 | -1.12 (-1.26,-0.98) | <0.001 | -0.56 (-0.67,-0.44) | <0.001 | 1990-1998 | -1.14 (-1.27,-1.01) | <0.001 | -0.56 (-0.67,-0.44) | <0.001 |
| | 1998-2006 | 0.81 (0.65,0.98) | <0.001 | | | 1998-2005 | -0.11 (-0.27,0.05) | 0.154 | | | 1998-2005 | -0.11 (-0.26,0.05) | 0.165 | | |
| | 2006-2009 | 2.27 (1.24,3.31) | <0.001 | | | 2005-2016 | 0.68 (0.63,0.74) | <0.001 | | | 2005-2016 | 0.70 (0.65,0.76) | <0.001 | | |

| | | | | | | | | | | | | | | | |
|---|---|---|---|---|---|---|---|---|---|---|---|---|---|---|---|
| | 2009-2017 | 1.30 (1.17,1.43) | <0.001 | | | 2016-2019 | -1.90 (-2.74,-1.05) | <0.001 | | | 2016-2019 | -1.92 (-2.75,-1.09) | <0.001 | | |
| | 2017-2021 | -2.63 (-3.04,-2.22) | <0.001 | | | 2019-2021 | -4.49 (-5.61,-3.37) | <0.001 | | | 2019-2021 | -4.54 (-5.63,-3.45) | <0.001 | | |
| Low SDI | 1990-2000 | -1.24 (-1.41,-1.06) | <0.001 | -0.05 (-0.17,0.07) | 0.376 | 1990-2001 | -1.68 (-1.83,-1.54) | <0.001 | -0.72 (-0.88,-0.56) | <0.001 | 1990-2001 | -1.68 (-1.83,-1.54) | <0.001 | -0.72 (-0.88,-0.57) | <0.001 |
| | 2000-2011 | 1.00 (0.87,1.12) | <0.001 | | | 2001-2007 | 0.50 (0.16,0.84) | 0.006 | | | 2001-2007 | 0.50 (0.17,0.84) | 0.006 | | |
| | 2011-2016 | 1.72 (1.26,2.17) | <0.001 | | | 2007-2010 | -0.49 (-1.69,0.74) | 0.413 | | | 2007-2010 | -0.48 (-1.67,0.73) | 0.416 | | |
| | 2016-2021 | -1.71 (-2.16,-1.25) | <0.001 | | | 2010-2016 | 1.10 (0.81,1.40) | <0.001 | | | 2010-2016 | 1.11 (0.81,1.40) | <0.001 | | |
| | | | | | | 2016-2021 | -2.35 (-2.80,-1.90) | <0.001 | | | 2016-2021 | -2.38 (-2.82,-1.93) | <0.001 | | |

ASIR, age-standardized incidence rate; APC, annual percent change; CI, confidence interval; AAPC, average annual percent change; ASMR, age-standardized mortality rate; ASDR, age-standardized disability-adjusted life years rate.

frontiers

**Table S3: The temporal trends of incidence, mortality and DALYs of kidney cancer in children from 1990 to 2021 in 204 countries or territories**

| | 1990 | | 2021 | | | 1990 | | 2021 | | | 1990 | | 2021 | | |
|---|---|---|---|---|---|---|---|---|---|---|---|---|---|---|---|
| Locations | Incidence cases, N(95% UI) | ASIR, Per 100,000 (95%UI) | Incidence cases, N(95% UI) | ASIR, Per 100,000 (95%UI) | EAPC of ASIR, % (95% CI) | Mortality cases, N(95% UI) | ASMR, Per 100,000 (95%UI) | Mortality cases, N(95% UI) | ASMR, Per 100,000 (95%UI) | EAPC of ASMR, % (95% CI) | Number of DALYs, N(95% UI) | ASDR, Per 100,000 (95%UI) | Number of DALYs, N(95% UI) | ASDR, Per 100,000 (95%UI) | EAPC of ASDR, % (95% CI) |
| Afghanistan | 24 (5,51) | 0.55 (0.13,1.19) | 82 (46,162) | 0.58 (0.32,1.14) | 0.62 (0.27,0.96) | 9 (2,17) | 0.2 (0.06,0.39) | 23 (14,38) | 0.17 (0.1,0.27) | -0.34 (-0.57,-0.11) | 760 (204,1461) | 17.63 (4.74,33.91) | 2053 (1257,3338) | 14.46 (8.85,23.5) | -0.35 (-0.58,-0.12) |
| Albania | 6 (4,8) | 0.53 (0.39,0.71) | 3 (2,5) | 0.78 (0.52,1.17) | 1.64 (1.16,2.13) | 2 (2,3) | 0.21 (0.16,0.28) | 1 (1,1) | 0.18 (0.12,0.27) | -0.28 (-0.64,0.07) | 206 (153,274) | 18.43 (13.71,24.55) | 72 (48,104) | 16.31 (10.92,23.53) | -0.25 (-0.58,0.08) |
| Algeria | 32 (23,45) | 0.3 (0.21,0.42) | 43 (29,63) | 0.33 (0.22,0.47) | 1.14 (0.8,1.48) | 8 (6,11) | 0.08 (0.06,0.1) | 7 (5,9) | 0.05 (0.04,0.07) | -0.62 (-0.9,-0.34) | 749 (563,986) | 6.98 (5.25,9.2) | 606 (444,830) | 4.55 (3.33,6.24) | -0.56 (-0.85,-0.27) |
| American Samoa | 0 (0,0) | 0.15 (0.1,0.21) | 0 (0,0) | 0.2 (0.12,0.31) | 1.21 (1.02,1.39) | 0 (0,0) | 0.05 (0.03,0.06) | 0 (0,0) | 0.06 (0.04,0.09) | 1 (0.8,1.21) | 1 (1,1) | 4.22 (2.99,5.73) | 1 (0,1) | 5.11 (3.12,7.77) | 0.98 (0.77,1.19) |
| Andorra | 0 (0,0) | 0.72 (0.4,1.15) | 0 (0,0) | 0.4 (0.28,0.57) | -1.43 (-1.85,-1.01) | 0 (0,0) | 0.16 (0.09,0.25) | 0 (0,0) | 0.05 (0.04,0.07) | -3.18 (-3.46,-2.9) | 1 (1,2) | 13.86 (7.7,21.79) | 0 (0,1) | 4.52 (3.29,5.98) | -3.16 (-3.46,-2.87) |
| Angola | 11 (4,23) | 0.23 (0.08,0.49) | 28 (18,41) | 0.18 (0.12,0.27) | -0.45 (-0.67,-0.23) | 7 (2,15) | 0.15 (0.05,0.31) | 14 (9,21) | 0.09 (0.06,0.13) | -1.32 (-1.57,-1.07) | 625 (211,1295) | 13.25 (4.46,27.46) | 1192 (753,1783) | 7.82 (4.94,11.69) | -1.34 (-1.59,-1.09) |

| | | | | | | | | | | | | | | | | |
|---|---|---|---|---|---|---|---|---|---|---|---|---|---|---|---|---|
| Antigua and Barbuda | 0 (0,0) | 0.37 (0.31,0.45) | 0 (0,0) | 0.49 (0.4,0.58) | 1.53 (1.22,1.85) | 0 (0,0) | 0.15 (0.12,0.18) | 0 (0,0) | 0.14 (0.12,0.16) | 0.41 (0.11,0.71) | 2 (2,3) | 12.71 (10.45,15.1) | 2 (2,2) | 11.93 (10.13,13.91) | 0.44 (0.15,0.74) |
| Argentina | 117 (100,139) | 1.15 (0.99,1.38) | 130 (102,161) | 1.27 (1.01,1.58) | 0.64 (0.29,0.99) | 17 (15,19) | 0.16 (0.14,0.19) | 12 (10,14) | 0.12 (0.1,0.14) | -0.66 (-0.94,-0.39) | 1462 (1282,1652) | 14.42 (12.65,16.3) | 1045 (862,1248) | 10.27 (8.47,12.25) | -0.62 (-0.9,-0.34) |
| Armenia | 5 (4,7) | 0.51 (0.34,0.71) | 3 (2,4) | 0.43 (0.29,0.61) | 1.22 (0.56,1.87) | 2 (1,3) | 0.19 (0.13,0.26) | 1 (0,1) | 0.12 (0.08,0.16) | 0.11 (-0.5,0.72) | 170 (116,235) | 16.31 (11.1,22.57) | 60 (41,85) | 10.07 (6.87,14.34) | 0.11 (-0.5,0.73) |
| Australia | 21 (19,25) | 0.56 (0.49,0.65) | 21 (17,26) | 0.45 (0.36,0.56) | 0.01 (-0.64,0.66) | 5 (5,6) | 0.13 (0.12,0.15) | 3 (2,3) | 0.05 (0.05,0.06) | -2.2 (-2.67,-1.74) | 440 (400,485) | 11.62 (10.55,12.82) | 230 (193,271) | 4.84 (4.06,5.7) | -2.14 (-2.62,-1.66) |
| Austria | 10 (8,11) | 0.71 (0.6,0.83) | 7 (6,9) | 0.55 (0.44,0.67) | -0.28 (-0.63,0.08) | 2 (2,3) | 0.18 (0.16,0.19) | 1 (1,1) | 0.07 (0.06,0.09) | -2.23 (-2.51,-1.95) | 206 (190,225) | 15.31 (14.06,16.67) | 86 (74,97) | 6.6 (5.71,7.5) | -2.19 (-2.46,-1.91) |
| Azerbaijan | 22 (13,34) | 0.89 (0.54,1.41) | 22 (13,33) | 0.93 (0.56,1.39) | 0.76 (0.47,1.06) | 9 (6,14) | 0.39 (0.24,0.59) | 7 (5,11) | 0.32 (0.19,0.47) | -0.13 (-0.32,0.06) | 817 (509,1259) | 33.67 (20.99,51.89) | 642 (391,944) | 27.21 (16.57,40.01) | -0.12 (-0.33,0.09) |
| Bahamas | 0 (0,0) | 0.46 (0.37,0.57) | 0 (0,0) | 0.43 (0.33,0.57) | -0.14 (-0.55,0.27) | 0 (0,0) | 0.2 (0.17,0.24) | 0 (0,0) | 0.14 (0.11,0.19) | -1.13 (-1.44,-0.82) | 14 (11,17) | 17.35 (14.2,20.93) | 10 (8,13) | 12.25 (9.35,16.14) | -1.15 (-1.45,-0.85) |
| Bahrain | 1 (1,2) | 0.69 (0.51,0.95) | 2 (1,3) | 0.71 (0.49,0.99) | 0.49 (0.25,0.72) | 0 (0,0) | 0.15 (0.12,0.19) | 0 (0,0) | 0.08 (0.06,0.1) | -1.92 (-2.11,-1.72) | 22 (17,27) | 13.23 (10.3,16.77) | 20 (15,26) | 6.88 (5.1,8.79) | -1.86 (-2.05,-1.66) |
| Bangladesh | 127 (50,211) | 0.26 (0.1,0.43) | 94 (67,127) | 0.2 (0.15,0.28) | -0.5 (-0.69,-0.31) | 77 (30,131) | 0.16 (0.06,0.27) | 35 (25,48) | 0.08 (0.05,0.1) | -2.09 (-2.25,-1.94) | 6705 (2625,11412) | 13.71 (5.37,23.33) | 3027 (2151,4160) | 6.61 (4.7,9.09) | -2.12 (-2.27,-1.96) |

| | | | | | | | | | | | | | | | |
|---|---|---|---|---|---|---|---|---|---|---|---|---|---|---|---|
| Barbados | 0 (0,0) | 0.65 (0.51,0.79) | 0 (0,0) | 0.56 (0.41,0.77) | 0.48 (0.07,0.89) | 0 (0,0) | 0.26 (0.21,0.3) | 0 (0,0) | 0.16 (0.11,0.21) | -0.64 (-1.03,-0.24) | 14 (11,16) | 21.67 (17.63,25.87) | 6 (5,8) | 13.33 (9.74,17.96) | -0.67 (-1.06,-0.28) |
| Belarus | 14 (11,18) | 0.58 (0.44,0.76) | 8 (5,10) | 0.48 (0.33,0.65) | 0.89 (0.33,1.45) | 4 (3,5) | 0.15 (0.12,0.19) | 1 (1,2) | 0.08 (0.06,0.1) | -0.86 (-1.45,-0.26) | 324 (255,410) | 13.48 (10.59,17.05) | 107 (80,143) | 6.79 (5.05,9.07) | -0.84 (-1.44,-0.24) |
| Belgium | 11 (9,13) | 0.59 (0.49,0.7) | 11 (8,13) | 0.55 (0.43,0.69) | -0.42 (-0.71,-0.12) | 3 (2,3) | 0.14 (0.12,0.16) | 1 (1,2) | 0.08 (0.06,0.09) | -2.22 (-2.44,-2) | 224 (195,256) | 12.41 (10.79,14.15) | 131 (107,155) | 6.85 (5.58,8.12) | -2.17 (-2.4,-1.94) |
| Belize | 1 (1,1) | 0.81 (0.69,0.93) | 0 (0,1) | 0.39 (0.31,0.47) | -1.62 (-1.84,-1.39) | 0 (0,0) | 0.38 (0.33,0.43) | 0 (0,0) | 0.14 (0.12,0.17) | -2.54 (-2.76,-2.33) | 27 (23,30) | 32.74 (28.65,36.89) | 15 (12,18) | 11.88 (9.91,14.42) | -2.58 (-2.8,-2.37) |
| Benin | 13 (8,17) | 0.52 (0.34,0.71) | 49 (32,71) | 0.81 (0.52,1.17) | 1.81 (1.62,1.99) | 8 (5,10) | 0.32 (0.21,0.43) | 24 (16,36) | 0.4 (0.25,0.59) | 1.1 (0.92,1.28) | 678 (453,913) | 28.01 (18.72,37.71) | 2140 (1367,3135) | 35.19 (22.48,51.56) | 1.1 (0.92,1.29) |
| Bermuda | 0 (0,0) | 0.72 (0.53,0.91) | 0 (0,0) | 0.78 (0.6,0.97) | 0.97 (0.56,1.38) | 0 (0,0) | 0.24 (0.19,0.31) | 0 (0,0) | 0.14 (0.11,0.17) | -1.1 (-1.46,-0.75) | 3 (2,3) | 21.09 (16.05,26.76) | 1 (1,1) | 12.16 (9.35,14.99) | -1.08 (-1.43,-0.72) |
| Bhutan | 1 (0,1) | 0.23 (0.11,0.4) | 1 (0,1) | 0.28 (0.16,0.46) | 0.54 (0.25,0.83) | 0 (0,1) | 0.14 (0.07,0.25) | 0 (0,0) | 0.11 (0.06,0.18) | -0.91 (-1.22,-0.61) | 32 (15,57) | 12.1 (5.71,21.88) | 18 (10,30) | 9.59 (5.39,15.79) | -0.92 (-1.22,-0.61) |
| Bolivia | 19 (12,26) | 0.69 (0.43,0.96) | 19 (13,27) | 0.55 (0.37,0.78) | -0.71 (-0.79,-0.63) | 11 (7,15) | 0.42 (0.27,0.58) | 8 (5,11) | 0.23 (0.15,0.32) | -1.96 (-2.06,-1.87) | 973 (615,1335) | 36.22 (22.9,49.72) | 683 (464,970) | 19.6 (13.3,27.81) | -1.97 (-2.07,-1.87) |
| Bosnia and Herzegovina | 3 (2,4) | 0.26 (0.2,0.33) | 2 (1,2) | 0.34 (0.26,0.45) | 2 (1.51,2.48) | 1 (1,1) | 0.09 (0.07,0.12) | 0 (0,0) | 0.08 (0.06,0.1) | 0.33 (-0.03,0.68) | 87 (67,109) | 7.95 (6.1,9.93) | 33 (26,42) | 6.81 (5.37,8.61) | 0.34 (-0.01,0.7) |

| | | | | | | | | | | | | | | | |
|---|---|---|---|---|---|---|---|---|---|---|---|---|---|---|---|
| Botswana | 1 (1,2) | 0.22 (0.14,0.32) | 4 (2,6) | 0.51 (0.31,0.82) | 3.93 (3.33,4.53) | 1 (0,1) | 0.12 (0.08,0.17) | 2 (1,2) | 0.22 (0.13,0.35) | 2.96 (2.46,3.46) | 61 (42,88) | 10.32 (7.14,14.91) | 131 (79,212) | 18.79 (11.37,30.35) | 2.99 (2.49,3.5) |
| Brazil | 306 (271,340) | 0.59 (0.52,0.66) | 211 (171,249) | 0.44 (0.35,0.52) | -0.16 (-0.6,0.28) | 149 (132,165) | 0.29 (0.25,0.32) | 67 (54,80) | 0.14 (0.11,0.17) | -1.5 (-1.92,-1.07) | 12881 (11416,14269) | 24.8 (21.98,27.47) | 5744 (4608,6870) | 11.92 (9.56,14.26) | -1.52 (-1.94,-1.09) |
| Brunei | 0 (0,0) | 0.27 (0.2,0.36) | 0 (0,0) | 0.31 (0.22,0.41) | 1.17 (0.9,1.44) | 0 (0,0) | 0.12 (0.09,0.16) | 0 (0,0) | 0.09 (0.07,0.12) | -0.23 (-0.46,0) | 9 (7,12) | 10.35 (7.6,13.43) | 7 (6,10) | 7.76 (5.82,10.39) | -0.19 (-0.41,0.04) |
| Bulgaria | 6 (5,7) | 0.32 (0.26,0.39) | 3 (3,4) | 0.35 (0.27,0.45) | 0.64 (0.15,1.13) | 2 (1,2) | 0.09 (0.08,0.11) | 1 (1,1) | 0.07 (0.06,0.09) | -0.34 (-0.9,0.23) | 141 (120,165) | 8.11 (6.91,9.5) | 63 (49,80) | 6.48 (5.05,8.23) | -0.34 (-0.9,0.23) |
| Burkina Faso | 20 (13,29) | 0.43 (0.27,0.61) | 74 (49,106) | 0.71 (0.47,1.02) | 2.2 (2.01,2.4) | 12 (8,18) | 0.26 (0.17,0.38) | 38 (26,55) | 0.37 (0.25,0.53) | 1.62 (1.43,1.81) | 1097 (710,1564) | 23.24 (15.04,33.14) | 3355 (2257,4802) | 32.35 (21.76,46.3) | 1.62 (1.43,1.81) |
| Burundi | 21 (14,30) | 0.8 (0.54,1.16) | 24 (7,52) | 0.41 (0.12,0.88) | -0.95 (-1.45,-0.44) | 13 (9,19) | 0.51 (0.36,0.72) | 13 (4,27) | 0.22 (0.06,0.46) | -1.52 (-2.01,-1.03) | 1184 (820,1660) | 45.17 (31.29,63.33) | 1142 (321,2376) | 19.51 (5.48,40.58) | -1.52 (-2.01,-1.03) |
| Cambodia | 18 (7,28) | 0.38 (0.16,0.61) | 19 (13,26) | 0.37 (0.26,0.52) | -0.02 (-0.28,0.23) | 8 (4,14) | 0.18 (0.08,0.29) | 7 (5,9) | 0.13 (0.1,0.17) | -1.07 (-1.25,-0.9) | 722 (310,1184) | 15.49 (6.65,25.4) | 582 (426,775) | 11.37 (8.32,15.15) | -1.09 (-1.27,-0.91) |
| Cameroon | 25 (16,37) | 0.51 (0.33,0.77) | 96 (61,148) | 0.71 (0.45,1.1) | 1.9 (1.59,2.22) | 15 (10,22) | 0.31 (0.2,0.45) | 46 (29,71) | 0.34 (0.22,0.53) | 1.23 (0.93,1.54) | 1320 (847,1922) | 27.05 (17.35,39.36) | 4067 (2553,6226) | 30.2 (18.96,46.23) | 1.23 (0.92,1.53) |
| Canada | 40 (34,47) | 0.69 (0.59,0.83) | 28 (23,35) | 0.46 (0.37,0.57) | -1.14 (-1.44,-0.84) | 6 (6,7) | 0.11 (0.1,0.13) | 3 (3,4) | 0.05 (0.04,0.06) | -2.27 (-2.45,-2.09) | 562 (496,636) | 9.77 (8.63,11.06) | 284 (238,336) | 4.6 (3.85,5.45) | -2.26 (-2.44,-2.08) |

| | | | | | | | | | | | | | | | |
|---|---|---|---|---|---|---|---|---|---|---|---|---|---|---|---|
| Cape Verde | 0 (0,0) | 0.12 (0.06,0.19) | 1 (1,2) | 0.97 (0.58,1.53) | 6.43 (5.16,7.71) | 0 (0,0) | 0.06 (0.03,0.09) | 0 (0,1) | 0.34 (0.2,0.55) | 5.04 (3.77,6.33) | 8 (5,13) | 5.08 (2.88,8.24) | 42 (25,70) | 29.6 (17.42,48.55) | 5.04 (3.77,6.32) |
| Central African Republic | 2 (1,4) | 0.18 (0.08,0.32) | 3 (2,5) | 0.14 (0.09,0.24) | -0.44 (-0.63,-0.26) | 2 (1,3) | 0.12 (0.05,0.21) | 2 (1,3) | 0.09 (0.06,0.14) | -0.72 (-0.9,-0.55) | 132 (56,222) | 10.79 (4.6,18.14) | 177 (111,281) | 7.75 (4.84,12.32) | -0.74 (-0.92,-0.57) |
| Chad | 10 (6,14) | 0.34 (0.22,0.48) | 51 (33,73) | 0.57 (0.37,0.81) | 2.2 (2.03,2.36) | 6 (4,9) | 0.22 (0.14,0.31) | 29 (19,42) | 0.32 (0.21,0.46) | 1.72 (1.56,1.88) | 554 (371,803) | 18.93 (12.66,27.45) | 2496 (1656,3649) | 27.69 (18.37,40.47) | 1.71 (1.55,1.87) |
| Chile | 21 (18,25) | 0.54 (0.46,0.64) | 16 (12,19) | 0.43 (0.34,0.53) | -0.33 (-0.54,-0.12) | 7 (6,8) | 0.18 (0.16,0.21) | 3 (2,3) | 0.07 (0.06,0.08) | -2.65 (-2.93,-2.38) | 623 (547,700) | 15.69 (13.78,17.64) | 223 (185,262) | 6.09 (5.07,7.17) | -2.62 (-2.9,-2.34) |
| China | 3295 (2682,4067) | 1.03 (0.84,1.28) | 1597 (1205,1992) | 0.62 (0.46,0.77) | -0.86 (-1.17,-0.55) | 1287 (1058,1581) | 0.4 (0.33,0.5) | 290 (216,362) | 0.11 (0.08,0.14) | -3.55 (-3.77,-3.33) | 113006 (92426,139290) | 35.49 (29.03,43.75) | 25365 (18782,31776) | 9.77 (7.23,12.24) | -3.54 (-3.76,-3.31) |
| Colombia | 52 (43,61) | 0.44 (0.37,0.52) | 43 (32,55) | 0.4 (0.3,0.52) | 1.1 (0.6,1.59) | 24 (21,28) | 0.21 (0.18,0.24) | 11 (9,14) | 0.11 (0.08,0.13) | -0.81 (-1.27,-0.35) | 2084 (1773,2412) | 17.87 (15.21,20.68) | 960 (739,1222) | 9.05 (6.96,11.51) | -0.82 (-1.28,-0.35) |
| Comoros | 2 (1,2) | 0.77 (0.48,1.11) | 2 (1,3) | 0.74 (0.35,1.33) | -0.04 (-0.36,0.28) | 1 (1,1) | 0.47 (0.3,0.69) | 1 (0,2) | 0.36 (0.16,0.64) | -0.83 (-1.09,-0.56) | 88 (57,129) | 41.27 (26.91,60.44) | 75 (34,134) | 31.4 (14.29,55.91) | -0.83 (-1.1,-0.57) |
| Cook Islands | 0 (0,0) | 0.08 (0.05,0.12) | 0 (0,0) | 0.08 (0.04,0.14) | -3 (-4.04,-1.94) | 0 (0,0) | 0.02 (0.01,0.03) | 0 (0,0) | 0.02 (0.01,0.03) | -3.97 (-5,-2.93) | 0 (0,0) | 1.88 (1.26,2.66) | 0 (0,0) | 1.43 (0.74,2.42) | -3.95 (-4.98,-2.92) |
| Costa Rica | 4 (3,5) | 0.35 (0.3,0.4) | 4 (4,5) | 0.43 (0.35,0.53) | 1.53 (1.31,1.75) | 1 (1,2) | 0.12 (0.11,0.14) | 1 (1,1) | 0.1 (0.09,0.12) | 0.16 (-0.06,0.38) | 122 (109,136) | 10.82 (9.65,12.07) | 91 (76,107) | 8.96 (7.51,10.47) | 0.11 (-0.11,0.33) |

| | | | | | | | | | | | | | | | |
|---|---|---|---|---|---|---|---|---|---|---|---|---|---|---|---|
| Croatia | 7 (5,8) | 0.67 (0.52,0.85) | 4 (3,5) | 0.6 (0.43,0.8) | 0.48 (0.08,0.88) | 1 (1,2) | 0.13 (0.11,0.16) | 0 (0,1) | 0.07 (0.06,0.09) | -1.15 (-1.52,-0.78) | 112 (91,135) | 11.33 (9.25,13.71) | 38 (29,50) | 6.41 (4.93,8.4) | -1.08 (-1.45,-0.71) |
| Cuba | 15 (12,18) | 0.6 (0.49,0.72) | 8 (7,10) | 0.47 (0.37,0.59) | 0.08 (-0.23,0.4) | 5 (4,6) | 0.21 (0.18,0.25) | 2 (2,2) | 0.11 (0.09,0.14) | -1.15 (-1.5,-0.81) | 454 (380,530) | 18.13 (15.17,21.16) | 172 (139,208) | 9.66 (7.84,11.71) | -1.14 (-1.48,-0.79) |
| Cyprus | 0 (0,0) | 0.18 (0.12,0.24) | 0 (0,1) | 0.19 (0.13,0.28) | 2.21 (1.5,2.91) | 0 (0,0) | 0.05 (0.04,0.07) | 0 (0,0) | 0.03 (0.02,0.04) | -0.76 (-1.24,-0.28) | 9 (7,13) | 4.75 (3.41,6.43) | 5 (4,8) | 2.5 (1.71,3.49) | -0.75 (-1.22,-0.27) |
| Czech Republic | 15 (13,17) | 0.67 (0.58,0.78) | 12 (9,15) | 0.71 (0.55,0.89) | 0.64 (0.2,1.09) | 4 (4,5) | 0.2 (0.17,0.22) | 2 (2,3) | 0.12 (0.1,0.15) | -0.98 (-1.31,-0.64) | 374 (332,423) | 16.97 (15.06,19.19) | 182 (144,225) | 10.58 (8.41,13.09) | -0.95 (-1.29,-0.61) |
| Democratic Republic of the Congo | 34 (15,59) | 0.19 (0.09,0.33) | 43 (23,70) | 0.11 (0.06,0.18) | -1.18 (-1.48,-0.88) | 21 (10,36) | 0.12 (0.05,0.2) | 22 (12,35) | 0.06 (0.03,0.09) | -1.77 (-2.03,-1.52) | 1821 (845,3153) | 10.29 (4.77,17.81) | 1893 (1036,3044) | 4.98 (2.73,8.01) | -1.8 (-2.06,-1.55) |
| Denmark | 3 (3,4) | 0.38 (0.33,0.45) | 2 (2,3) | 0.26 (0.2,0.31) | -1.06 (-1.31,-0.81) | 1 (1,1) | 0.13 (0.12,0.15) | 0 (0,1) | 0.05 (0.04,0.06) | -3.27 (-3.42,-3.12) | 103 (92,116) | 11.67 (10.4,13.09) | 41 (33,48) | 4.29 (3.49,5) | -3.26 (-3.42,-3.1) |
| Djibouti | 1 (1,2) | 0.76 (0.43,1.25) | 2 (1,4) | 0.6 (0.24,1.09) | -0.27 (-0.64,0.1) | 1 (0,1) | 0.45 (0.26,0.73) | 1 (0,2) | 0.28 (0.11,0.52) | -1 (-1.4,-0.59) | 69 (39,112) | 39.66 (22.52,64.56) | 102 (40,188) | 24.75 (9.76,45.51) | -1 (-1.41,-0.59) |
| Dominica | 0 (0,0) | 0.4 (0.3,0.5) | 0 (0,0) | 0.85 (0.62,1.12) | 2.69 (2.51,2.87) | 0 (0,0) | 0.19 (0.14,0.24) | 0 (0,0) | 0.32 (0.24,0.43) | 2 (1.85,2.15) | 4 (3,5) | 16.08 (12.03,20.59) | 4 (3,5) | 27.55 (20.27,36.32) | 1.99 (1.85,2.14) |
| Dominican Republic | 20 (14,27) | 0.74 (0.51,1.01) | 18 (12,30) | 0.63 (0.41,1.01) | -0.71 (-0.96,-0.46) | 10 (7,14) | 0.39 (0.27,0.51) | 7 (5,12) | 0.24 (0.15,0.4) | -1.63 (-1.92,-1.34) | 909 (627,1202) | 33.72 (23.25,44.58) | 616 (388,1028) | 20.95 (13.21,34.99) | -1.65 (-1.94,-1.36) |

| | | | | | | | | | | | | | | | |
|---|---|---|---|---|---|---|---|---|---|---|---|---|---|---|---|
| Ecuador | 13 (11,15) | 0.34 (0.29,0.4) | 15 (11,20) | 0.3 (0.22,0.4) | -0.16 (-0.53,0.2) | 7 (6,8) | 0.17 (0.15,0.2) | 5 (4,6) | 0.1 (0.07,0.12) | -1.59 (-1.93,-1.25) | 571 (496,658) | 14.77 (12.82,17.03) | 411 (318,520) | 8.1 (6.27,10.25) | -1.63 (-1.97,-1.29) |
| Egypt | 124 (91,169) | 0.56 (0.41,0.76) | 144 (101,204) | 0.39 (0.27,0.55) | -0.57 (-0.83,-0.31) | 36 (28,46) | 0.16 (0.13,0.21) | 25 (19,34) | 0.07 (0.05,0.09) | -2.17 (-2.4,-1.94) | 3139 (2443,3985) | 14.15 (11.02,17.97) | 2236 (1658,3027) | 6.07 (4.5,8.21) | -2.14 (-2.38,-1.91) |
| El Salvador | 8 (7,11) | 0.39 (0.3,0.49) | 5 (4,7) | 0.3 (0.22,0.41) | -0.69 (-0.84,-0.54) | 4 (3,5) | 0.2 (0.16,0.24) | 2 (1,2) | 0.09 (0.07,0.12) | -2.41 (-2.67,-2.16) | 370 (294,448) | 17.16 (13.65,20.74) | 138 (101,187) | 7.6 (5.56,10.3) | -2.47 (-2.72,-2.21) |
| Equatorial Guinea | 0 (0,1) | 0.18 (0.08,0.31) | 1 (1,2) | 0.21 (0.1,0.39) | 0.61 (0.42,0.81) | 0 (0,0) | 0.12 (0.05,0.19) | 0 (0,1) | 0.09 (0.04,0.15) | -1.18 (-1.36,-0.99) | 20 (9,33) | 10.1 (4.69,16.91) | 43 (22,76) | 7.34 (3.72,13.01) | -1.21 (-1.39,-1.02) |
| Eritrea | 9 (6,13) | 0.55 (0.35,0.84) | 15 (6,27) | 0.59 (0.24,1.07) | 0.22 (0.02,0.42) | 6 (4,8) | 0.36 (0.23,0.53) | 8 (3,15) | 0.31 (0.12,0.59) | -0.38 (-0.57,-0.18) | 502 (324,744) | 31.56 (20.37,46.73) | 696 (268,1301) | 27.58 (10.63,51.52) | -0.38 (-0.57,-0.18) |
| Estonia | 3 (3,4) | 1 (0.81,1.22) | 1 (1,1) | 0.52 (0.38,0.67) | -0.95 (-1.37,-0.52) | 1 (1,1) | 0.23 (0.19,0.27) | 0 (0,0) | 0.07 (0.05,0.08) | -3.08 (-3.44,-2.73) | 71 (60,83) | 20.24 (17.13,23.78) | 13 (10,16) | 5.94 (4.73,7.37) | -3.04 (-3.4,-2.68) |
| Ethiopia | 162 (35,374) | 0.66 (0.14,1.53) | 234 (151,378) | 0.53 (0.34,0.85) | -0.88 (-1.32,-0.43) | 108 (24,244) | 0.44 (0.1,1) | 110 (71,176) | 0.25 (0.16,0.4) | -2.04 (-2.44,-1.63) | 9449 (2108,21431) | 38.78 (8.65,87.96) | 9642 (6202,15377) | 21.74 (13.98,34.67) | -2.04 (-2.44,-1.63) |
| Fiji | 0 (0,0) | 0.09 (0.06,0.14) | 0 (0,1) | 0.18 (0.12,0.27) | 2.5 (2.28,2.72) | 0 (0,0) | 0.03 (0.02,0.05) | 0 (0,0) | 0.06 (0.04,0.08) | 2.29 (2.06,2.51) | 8 (5,12) | 2.86 (1.77,4.35) | 14 (10,20) | 5.29 (3.54,7.51) | 2.31 (2.09,2.53) |
| Finland | 5 (4,6) | 0.52 (0.43,0.65) | 5 (3,6) | 0.53 (0.4,0.7) | -0.22 (-0.87,0.43) | 1 (1,1) | 0.12 (0.1,0.14) | 1 (0,1) | 0.07 (0.06,0.09) | -2.04 (-2.56,-1.53) | 101 (88,115) | 10.42 (9.13,11.91) | 53 (42,65) | 6.22 (4.97,7.62) | -2.02 (-2.54,-1.49) |

| | | | | | | | | | | | | | | | |
|---|---|---|---|---|---|---|---|---|---|---|---|---|---|---|---|
| France | 87 (73,103) | 0.74 (0.62,0.88) | 125 (101,154) | 1.08 (0.87,1.33) | 1.47 (1.32,1.61) | 16 (14,18) | 0.13 (0.12,0.15) | 10 (9,12) | 0.09 (0.08,0.1) | -1.38 (-1.58,-1.18) | 1383 (1248,1551) | 11.81 (10.66,13.24) | 933 (795,1088) | 8.04 (6.85,9.37) | -1.24 (-1.44,-1.03) |
| Gabon | 1 (0,1) | 0.18 (0.11,0.31) | 1 (1,2) | 0.23 (0.15,0.34) | 1.25 (1.02,1.49) | 0 (0,1) | 0.1 (0.06,0.18) | 1 (0,1) | 0.1 (0.06,0.14) | 0.22 (0,0.45) | 37 (22,63) | 9.1 (5.49,15.44) | 53 (34,80) | 8.29 (5.37,12.45) | 0.21 (-0.02,0.44) |
| Gambia | 2 (1,2) | 0.36 (0.24,0.52) | 3 (1,6) | 0.3 (0.14,0.59) | -0.74 (-1.06,-0.42) | 1 (1,1) | 0.2 (0.14,0.29) | 1 (1,3) | 0.14 (0.07,0.27) | -1.37 (-1.69,-1.04) | 83 (55,115) | 17.94 (11.92,25) | 121 (57,231) | 12.22 (5.72,23.23) | -1.37 (-1.7,-1.04) |
| Georgia | 7 (4,10) | 0.51 (0.3,0.75) | 2 (1,3) | 0.27 (0.19,0.36) | -0.9 (-1.41,-0.38) | 2 (1,3) | 0.17 (0.1,0.24) | 1 (0,1) | 0.08 (0.06,0.12) | -0.77 (-1.34,-0.2) | 198 (124,284) | 14.45 (9.03,20.71) | 54 (39,75) | 7.36 (5.26,10.18) | -0.75 (-1.33,-0.18) |
| Germany | 105 (90,125) | 0.81 (0.69,0.96) | 77 (64,96) | 0.65 (0.53,0.8) | -0.46 (-0.66,-0.26) | 22 (20,24) | 0.17 (0.15,0.19) | 10 (9,12) | 0.08 (0.07,0.1) | -1.82 (-2.02,-1.63) | 1903 (1716,2107) | 14.7 (13.26,16.28) | 887 (764,1037) | 7.42 (6.39,8.67) | -1.78 (-1.97,-1.59) |
| Ghana | 22 (13,35) | 0.33 (0.2,0.52) | 73 (38,119) | 0.57 (0.3,0.93) | 2.43 (2.18,2.68) | 13 (8,20) | 0.19 (0.12,0.3) | 33 (17,53) | 0.26 (0.13,0.41) | 1.69 (1.43,1.95) | 1131 (687,1760) | 16.83 (10.23,26.2) | 2921 (1506,4673) | 22.67 (11.69,36.27) | 1.68 (1.42,1.94) |
| Greece | 8 (7,9) | 0.39 (0.34,0.45) | 5 (4,6) | 0.36 (0.3,0.43) | 0.44 (-0.01,0.88) | 2 (2,2) | 0.09 (0.08,0.1) | 1 (1,1) | 0.06 (0.05,0.06) | -0.84 (-1.2,-0.48) | 154 (142,166) | 7.63 (7.03,8.19) | 69 (59,79) | 4.92 (4.23,5.64) | -0.77 (-1.13,-0.41) |
| Greenland | 0 (0,0) | 1.08 (0.83,1.4) | 0 (0,0) | 0.73 (0.5,1.03) | -0.86 (-1.16,-0.56) | 0 (0,0) | 0.44 (0.34,0.57) | 0 (0,0) | 0.18 (0.13,0.25) | -2.67 (-2.97,-2.38) | 6 (4,7) | 38.7 (29.92,49.66) | 2 (1,3) | 15.68 (10.9,22.03) | -2.66 (-2.96,-2.35) |
| Grenada | 0 (0,0) | 0.38 (0.31,0.48) | 0 (0,0) | 0.48 (0.4,0.59) | 1.45 (1.13,1.76) | 0 (0,0) | 0.19 (0.15,0.23) | 0 (0,0) | 0.17 (0.14,0.2) | 0.49 (0.2,0.79) | 5 (4,7) | 15.98 (12.84,19.48) | 3 (3,4) | 14.51 (11.93,17.07) | 0.47 (0.18,0.75) |

| | | | | | | | | | | | | | | | |
|---|---|---|---|---|---|---|---|---|---|---|---|---|---|---|---|
| Guam | 0 (0,0) | 0.32 (0.23,0.42) | 0 (0,0) | 0.43 (0.29,0.63) | 2.3 (1.71,2.91) | 0 (0,0) | 0.08 (0.06,0.1) | 0 (0,0) | 0.1 (0.07,0.15) | 2.16 (1.72,2.61) | 3 (2,4) | 6.96 (5.08,9.3) | 3 (2,5) | 9.39 (6.39,13.49) | 2.17 (1.73,2.61) |
| Guatemala | 24 (21,28) | 0.6 (0.52,0.68) | 17 (13,22) | 0.34 (0.27,0.44) | -1.16 (-1.43,-0.89) | 14 (13,16) | 0.35 (0.31,0.39) | 6 (5,8) | 0.13 (0.11,0.16) | -2.46 (-2.8,-2.12) | 1229 (1087,1373) | 30.27 (26.75,33.82) | 557 (447,697) | 11.28 (9.07,14.12) | -2.47 (-2.81,-2.13) |
| Guinea | 22 (15,30) | 0.8 (0.53,1.1) | 36 (12,67) | 0.59 (0.21,1.11) | -0.4 (-0.6,-0.2) | 14 (9,20) | 0.51 (0.34,0.72) | 19 (7,37) | 0.32 (0.11,0.61) | -0.88 (-1.09,-0.67) | 1235 (819,1740) | 44.89 (29.75,63.23) | 1708 (585,3239) | 28.24 (9.67,53.57) | -0.88 (-1.08,-0.67) |
| Guinea-Bissau | 2 (1,3) | 0.44 (0.27,0.65) | 4 (2,6) | 0.4 (0.22,0.67) | 0.45 (-0.03,0.92) | 1 (1,2) | 0.3 (0.18,0.43) | 2 (1,3) | 0.22 (0.12,0.38) | -0.12 (-0.6,0.36) | 125 (76,183) | 25.87 (15.8,37.99) | 176 (96,299) | 19.59 (10.65,33.27) | -0.13 (-0.61,0.36) |
| Guyana | 1 (1,2) | 0.46 (0.37,0.56) | 1 (1,1) | 0.48 (0.37,0.63) | 1.29 (0.77,1.82) | 1 (1,1) | 0.26 (0.21,0.31) | 0 (0,1) | 0.21 (0.17,0.27) | 0.58 (0.07,1.09) | 66 (53,79) | 22.56 (18.04,27.02) | 39 (30,50) | 18.17 (14.06,23.53) | 0.55 (0.05,1.05) |
| Haiti | 20 (7,39) | 0.75 (0.25,1.44) | 26 (13,51) | 0.6 (0.29,1.17) | -0.25 (-0.44,-0.06) | 14 (4,26) | 0.5 (0.16,0.94) | 14 (7,28) | 0.33 (0.16,0.63) | -0.9 (-1.11,-0.69) | 1170 (381,2204) | 43.11 (14.03,81.22) | 1226 (607,2378) | 28.16 (13.95,54.63) | -0.91 (-1.12,-0.7) |
| Honduras | 5 (4,7) | 0.24 (0.19,0.31) | 5 (2,8) | 0.14 (0.06,0.24) | -1.85 (-1.98,-1.71) | 3 (2,4) | 0.14 (0.11,0.17) | 2 (1,3) | 0.06 (0.03,0.1) | -2.63 (-2.72,-2.53) | 257 (205,324) | 11.63 (9.28,14.64) | 171 (72,290) | 5.22 (2.19,8.86) | -2.62 (-2.71,-2.53) |
| Hungary | 9 (8,11) | 0.44 (0.38,0.51) | 6 (4,8) | 0.42 (0.31,0.54) | 0.35 (-0.41,1.11) | 3 (3,3) | 0.14 (0.12,0.16) | 1 (1,1) | 0.09 (0.07,0.11) | -1.02 (-1.66,-0.37) | 258 (229,290) | 12.1 (10.75,13.59) | 103 (82,128) | 7.45 (5.93,9.24) | -0.98 (-1.63,-0.34) |
| Iceland | 1 (1,1) | 1.06 (0.84,1.31) | 1 (0,1) | 0.9 (0.64,1.23) | -0.49 (-1.12,0.16) | 0 (0,0) | 0.22 (0.18,0.26) | 0 (0,0) | 0.1 (0.08,0.13) | -2.32 (-2.84,-1.79) | 12 (10,15) | 19.3 (16.22,22.91) | 6 (5,8) | 9.05 (6.67,11.48) | -2.26 (-2.79,-1.74) |

| | | | | | | | | | | | | | | | |
|---|---|---|---|---|---|---|---|---|---|---|---|---|---|---|---|
| India | 512 (310,715) | 0.16 (0.1,0.22) | 484 (326,657) | 0.13 (0.09,0.18) | -0.49 (-0.6,-0.39) | 301 (181,424) | 0.09 (0.06,0.13) | 197 (134,267) | 0.05 (0.04,0.07) | -1.72 (-1.85,-1.6) | 26273 (15761,37063) | 8.05 (4.83,11.35) | 17272 (11716,23440) | 4.71 (3.2,6.4) | -1.71 (-1.83,-1.59) |
| Indonesia | 240 (122,375) | 0.35 (0.18,0.55) | 250 (176,344) | 0.37 (0.26,0.51) | 0.17 (0.1,0.24) | 98 (50,154) | 0.14 (0.07,0.23) | 83 (58,115) | 0.12 (0.09,0.17) | -0.44 (-0.59,-0.3) | 8477 (4294,13454) | 12.51 (6.34,19.86) | 7167 (4996,10041) | 10.65 (7.43,14.92) | -0.46 (-0.6,-0.31) |
| Iran | 317 (197,440) | 1.25 (0.78,1.74) | 167 (122,237) | 0.83 (0.6,1.18) | 0.2 (-0.39,0.79) | 72 (51,93) | 0.28 (0.2,0.37) | 23 (18,29) | 0.12 (0.09,0.14) | -1.38 (-1.93,-0.83) | 6314 (4458,8258) | 24.87 (17.56,32.53) | 2032 (1520,2529) | 10.07 (7.53,12.53) | -1.35 (-1.92,-0.79) |
| Iraq | 58 (34,87) | 0.71 (0.41,1.06) | 105 (68,161) | 0.78 (0.5,1.2) | 1.19 (0.89,1.5) | 16 (10,25) | 0.2 (0.12,0.3) | 19 (12,28) | 0.14 (0.09,0.21) | -0.63 (-0.88,-0.39) | 1419 (864,2180) | 17.23 (10.49,26.46) | 1661 (1029,2480) | 12.34 (7.64,18.43) | -0.61 (-0.85,-0.36) |
| Ireland | 4 (4,5) | 0.44 (0.36,0.53) | 3 (3,4) | 0.32 (0.26,0.39) | 0.13 (-0.48,0.74) | 1 (1,1) | 0.12 (0.1,0.13) | 0 (0,0) | 0.04 (0.04,0.05) | -2.38 (-2.85,-1.91) | 98 (85,112) | 9.95 (8.7,11.39) | 37 (31,44) | 3.74 (3.13,4.39) | -2.3 (-2.79,-1.82) |
| Israel | 7 (6,9) | 0.47 (0.39,0.57) | 9 (7,11) | 0.33 (0.25,0.42) | -0.46 (-1.08,0.16) | 2 (2,2) | 0.13 (0.12,0.15) | 1 (1,2) | 0.05 (0.04,0.06) | -2.57 (-3.06,-2.09) | 179 (157,205) | 11.7 (10.25,13.38) | 116 (94,144) | 4.42 (3.57,5.46) | -2.53 (-3.02,-2.03) |
| Italy | 93 (81,108) | 1.01 (0.88,1.17) | 64 (53,77) | 0.84 (0.69,1.01) | -0.69 (-1.03,-0.36) | 18 (17,19) | 0.19 (0.18,0.2) | 7 (6,8) | 0.09 (0.08,0.1) | -2.32 (-2.57,-2.07) | 1544 (1471,1630) | 16.73 (15.94,17.66) | 628 (551,705) | 8.26 (7.25,9.28) | -2.27 (-2.52,-2.02) |
| Ivory Coast | 13 (9,20) | 0.24 (0.15,0.35) | 31 (13,55) | 0.27 (0.12,0.48) | 0.9 (0.6,1.2) | 8 (5,12) | 0.14 (0.09,0.21) | 15 (6,26) | 0.13 (0.06,0.22) | 0.15 (-0.13,0.44) | 702 (448,1069) | 12.3 (7.85,18.75) | 1298 (559,2286) | 11.22 (4.83,19.75) | 0.15 (-0.13,0.44) |
| Jamaica | 6 (4,7) | 0.66 (0.53,0.82) | 3 (2,4) | 0.48 (0.36,0.61) | -0.88 (-1.07,-0.69) | 2 (2,3) | 0.27 (0.22,0.33) | 1 (1,1) | 0.15 (0.12,0.18) | -1.81 (-2.05,-1.58) | 196 (161,240) | 23.51 (19.28,28.69) | 73 (57,92) | 12.53 (9.75,15.74) | -1.83 (-2.07,-1.59) |

| | | | | | | | | | | | | | | | |
|---|---|---|---|---|---|---|---|---|---|---|---|---|---|---|---|
| Japan | 69 (61,78) | 0.3 (0.27,0.34) | 51 (44,60) | 0.33 (0.28,0.39) | 0.44 (0.05,0.84) | 16 (16,17) | 0.07 (0.07,0.07) | 7 (6,7) | 0.04 (0.04,0.05) | -1.4 (-1.65,-1.15) | 1402 (1361,1445) | 6.07 (5.89,6.26) | 600 (553,647) | 3.89 (3.58,4.19) | -1.34 (-1.61,-1.08) |
| Jordan | 7 (6,9) | 0.44 (0.34,0.56) | 23 (16,33) | 0.64 (0.45,0.91) | 1.89 (1.61,2.17) | 2 (1,2) | 0.11 (0.09,0.14) | 3 (3,5) | 0.1 (0.07,0.13) | -0.2 (-0.42,0.01) | 161 (127,203) | 9.83 (7.76,12.4) | 308 (228,420) | 8.49 (6.28,11.56) | -0.17 (-0.39,0.05) |
| Kazakhstan | 36 (30,44) | 0.7 (0.58,0.86) | 26 (20,33) | 0.47 (0.37,0.6) | -0.49 (-0.87,-0.11) | 14 (12,17) | 0.28 (0.24,0.33) | 7 (6,9) | 0.14 (0.11,0.17) | -1.75 (-2.15,-1.36) | 1256 (1063,1509) | 24.17 (20.45,29.05) | 643 (511,797) | 11.85 (9.42,14.68) | -1.72 (-2.12,-1.33) |
| Kenya | 25 (15,35) | 0.22 (0.13,0.31) | 29 (19,40) | 0.15 (0.1,0.21) | 0.06 (-0.45,0.58) | 13 (8,19) | 0.12 (0.07,0.17) | 13 (9,18) | 0.07 (0.05,0.1) | -0.35 (-0.87,0.17) | 1166 (689,1695) | 10.44 (6.17,15.17) | 1162 (773,1619) | 6.23 (4.14,8.67) | -0.38 (-0.89,0.14) |
| Kiribati | 0 (0,0) | 0.26 (0.16,0.39) | 0 (0,0) | 0.29 (0.12,0.55) | 0.33 (0.06,0.6) | 0 (0,0) | 0.13 (0.08,0.21) | 0 (0,0) | 0.15 (0.06,0.29) | 0.2 (-0.08,0.48) | 4 (2,5) | 11.93 (7.34,18.55) | 5 (2,11) | 12.95 (5.04,26.08) | 0.2 (-0.08,0.49) |
| Kuwait | 3 (2,4) | 0.54 (0.43,0.68) | 10 (8,13) | 1.18 (0.91,1.57) | 0.99 (0.14,1.84) | 1 (0,1) | 0.09 (0.08,0.11) | 1 (1,1) | 0.11 (0.09,0.14) | -0.31 (-0.95,0.34) | 46 (39,54) | 8.25 (6.98,9.8) | 84 (69,104) | 9.98 (8.11,12.31) | -0.27 (-0.92,0.38) |
| Kyrgyzstan | 7 (5,9) | 0.39 (0.3,0.51) | 10 (8,12) | 0.44 (0.35,0.54) | 0.19 (-0.1,0.49) | 3 (2,4) | 0.17 (0.13,0.22) | 3 (3,4) | 0.14 (0.12,0.17) | -0.63 (-0.87,-0.4) | 253 (193,322) | 15.06 (11.53,19.19) | 282 (229,339) | 12.38 (10.08,14.89) | -0.66 (-0.88,-0.43) |
| Laos | 7 (2,12) | 0.38 (0.09,0.68) | 9 (5,14) | 0.37 (0.23,0.59) | 0.08 (-0.05,0.22) | 4 (1,6) | 0.19 (0.05,0.35) | 3 (2,5) | 0.14 (0.09,0.23) | -0.86 (-0.94,-0.77) | 305 (78,564) | 16.57 (4.23,30.59) | 285 (177,461) | 12.43 (7.72,20.06) | -0.88 (-0.96,-0.79) |
| Latvia | 4 (3,5) | 0.66 (0.56,0.79) | 1 (1,2) | 0.46 (0.35,0.62) | 0.15 (-0.46,0.77) | 1 (1,1) | 0.15 (0.13,0.17) | 0 (0,0) | 0.07 (0.06,0.09) | -1.07 (-1.71,-0.42) | 75 (65,86) | 13.15 (11.44,15.13) | 19 (15,23) | 6.26 (4.98,7.86) | -1.06 (-1.7,-0.42) |

| | | | | | | | | | | | | | | | |
|---|---|---|---|---|---|---|---|---|---|---|---|---|---|---|---|
| Lebanon | 5 (3,7) | 0.47 (0.32,0.67) | 8 (4,13) | 0.59 (0.28,1.02) | 1.21 (0.9,1.52) | 1 (1,2) | 0.12 (0.08,0.17) | 1 (1,2) | 0.09 (0.04,0.16) | -0.56 (-0.77,-0.35) | 110 (77,156) | 10.54 (7.34,14.87) | 105 (47,180) | 8.21 (3.7,14.06) | -0.51 (-0.73,-0.3) |
| Lesotho | 1 (1,2) | 0.17 (0.11,0.26) | 2 (1,3) | 0.28 (0.18,0.43) | 2.42 (1.98,2.86) | 1 (0,1) | 0.1 (0.07,0.15) | 1 (1,1) | 0.15 (0.1,0.21) | 2.16 (1.76,2.55) | 58 (40,88) | 8.5 (5.85,12.9) | 81 (53,116) | 12.8 (8.38,18.42) | 2.12 (1.72,2.52) |
| Liberia | 6 (3,11) | 0.52 (0.23,1) | 11 (7,17) | 0.5 (0.31,0.77) | 0.95 (0.18,1.72) | 4 (2,7) | 0.32 (0.14,0.63) | 5 (3,8) | 0.24 (0.15,0.37) | 0 (-0.76,0.76) | 322 (143,623) | 28.46 (12.62,55.13) | 453 (289,708) | 20.74 (13.22,32.37) | 0 (-0.76,0.76) |
| Libya | 17 (11,23) | 0.93 (0.6,1.29) | 17 (10,26) | 1.13 (0.69,1.74) | 1.47 (1.08,1.87) | 4 (3,6) | 0.24 (0.17,0.31) | 3 (2,5) | 0.2 (0.13,0.33) | -0.01 (-0.22,0.2) | 383 (266,502) | 21.14 (14.69,27.69) | 270 (165,431) | 18.13 (11.08,28.86) | 0 (-0.21,0.22) |
| Lithuania | 5 (5,6) | 0.65 (0.55,0.78) | 2 (1,2) | 0.44 (0.34,0.56) | -1.34 (-1.77,-0.91) | 1 (1,2) | 0.17 (0.15,0.2) | 0 (0,0) | 0.09 (0.07,0.1) | -2.15 (-2.6,-1.69) | 124 (108,145) | 14.96 (13.05,17.4) | 30 (25,37) | 7.44 (6.12,9.03) | -2.17 (-2.63,-1.72) |
| Luxembourg | 0 (0,0) | 0.35 (0.29,0.42) | 0 (0,0) | 0.21 (0.17,0.27) | -1.7 (-2.04,-1.36) | 0 (0,0) | 0.09 (0.08,0.1) | 0 (0,0) | 0.03 (0.02,0.04) | -3.9 (-4.35,-3.45) | 5 (5,6) | 7.91 (6.96,8.84) | 3 (2,3) | 2.68 (2.2,3.32) | -3.8 (-4.25,-3.35) |
| Madagascar | 38 (27,52) | 0.7 (0.5,0.96) | 53 (23,92) | 0.45 (0.2,0.78) | -1.04 (-1.23,-0.84) | 23 (17,32) | 0.43 (0.31,0.59) | 27 (11,48) | 0.23 (0.1,0.41) | -1.58 (-1.78,-1.38) | 2063 (1498,2810) | 37.81 (27.46,51.51) | 2400 (999,4222) | 20.45 (8.51,35.98) | -1.59 (-1.8,-1.39) |
| Malawi | 97 (60,135) | 2.13 (1.31,2.97) | 105 (36,198) | 1.29 (0.44,2.44) | -1.48 (-1.65,-1.3) | 60 (38,85) | 1.32 (0.83,1.87) | 53 (18,99) | 0.65 (0.22,1.22) | -2.14 (-2.32,-1.97) | 5301 (3339,7505) | 116.52 (73.39,164.96) | 4643 (1585,8689) | 57.15 (19.51,106.96) | -2.15 (-2.33,-1.98) |
| Malaysia | 12 (8,17) | 0.19 (0.12,0.26) | 13 (10,17) | 0.17 (0.13,0.22) | -0.2 (-0.57,0.18) | 4 (3,6) | 0.06 (0.04,0.09) | 3 (2,4) | 0.04 (0.03,0.05) | -1.31 (-1.67,-0.95) | 355 (230,495) | 5.39 (3.5,7.53) | 266 (203,345) | 3.5 (2.67,4.53) | -1.3 (-1.68,-0.93) |

| | | | | | | | | | | | | | | | |
|---|---|---|---|---|---|---|---|---|---|---|---|---|---|---|---|
| Maldives | 0 (0,0) | 0.23 (0.09,0.46) | 0 (0,0) | 0.31 (0.2,0.46) | 1.61 (1.3,1.92) | 0 (0,0) | 0.09 (0.04,0.18) | 0 (0,0) | 0.07 (0.04,0.11) | -0.42 (-0.68,-0.15) | 8 (3,16) | 7.95 (3.26,15.63) | 6 (4,9) | 6.05 (3.87,9.24) | -0.38 (-0.65,-0.11) |
| Mali | 40 (25,59) | 0.97 (0.61,1.42) | 80 (33,151) | 0.69 (0.29,1.3) | -0.72 (-0.9,-0.55) | 25 (16,36) | 0.6 (0.38,0.88) | 40 (17,77) | 0.35 (0.15,0.66) | -1.37 (-1.53,-1.2) | 2180 (1387,3192) | 52.79 (33.58,77.29) | 3557 (1478,6750) | 30.73 (12.77,58.31) | -1.37 (-1.53,-1.2) |
| Malta | 0 (0,0) | 0.45 (0.34,0.56) | 1 (0,1) | 0.79 (0.59,1.01) | 1.72 (1.37,2.06) | 0 (0,0) | 0.12 (0.09,0.14) | 0 (0,0) | 0.12 (0.09,0.15) | -0.34 (-0.78,0.1) | 9 (7,11) | 10.31 (8,12.51) | 7 (5,8) | 10.29 (7.89,12.73) | -0.29 (-0.71,0.14) |
| Marshall Islands | 0 (0,0) | 0.07 (0.04,0.12) | 0 (0,0) | 0.11 (0.05,0.21) | 1.38 (1,1.76) | 0 (0,0) | 0.03 (0.02,0.05) | 0 (0,0) | 0.04 (0.02,0.09) | 1.14 (0.68,1.6) | 1 (0,1) | 2.55 (1.39,4.33) | 1 (0,1) | 3.73 (1.76,7.66) | 1.14 (0.68,1.6) |
| Mauritania | 3 (2,5) | 0.37 (0.23,0.57) | 11 (6,17) | 0.59 (0.35,0.9) | 1.53 (1.25,1.82) | 2 (1,3) | 0.23 (0.15,0.34) | 5 (3,7) | 0.25 (0.15,0.39) | 0.37 (0.06,0.67) | 184 (118,279) | 19.89 (12.78,30.21) | 403 (240,630) | 21.76 (12.94,34) | 0.37 (0.06,0.67) |
| Mauritius | 1 (1,1) | 0.22 (0.19,0.26) | 0 (0,1) | 0.21 (0.18,0.25) | -2.94 (-4.72,-1.12) | 0 (0,0) | 0.06 (0.06,0.07) | 0 (0,0) | 0.05 (0.04,0.06) | -3.36 (-5.06,-1.63) | 18 (17,20) | 5.53 (5.02,6.13) | 9 (8,10) | 4.39 (3.71,5.04) | -3.36 (-5.07,-1.63) |
| Mexico | 242 (219,270) | 0.72 (0.65,0.81) | 139 (112,173) | 0.43 (0.35,0.54) | -1.14 (-1.41,-0.86) | 118 (107,131) | 0.35 (0.32,0.39) | 44 (35,54) | 0.14 (0.11,0.17) | -2.46 (-2.69,-2.23) | 10214 (9240,11345) | 30.57 (27.65,33.95) | 3772 (3024,4697) | 11.76 (9.43,14.65) | -2.48 (-2.71,-2.25) |
| Micronesia | 0 (0,0) | 0.11 (0.06,0.16) | 0 (0,0) | 0.09 (0.04,0.16) | -0.35 (-0.51,-0.2) | 0 (0,0) | 0.05 (0.03,0.07) | 0 (0,0) | 0.03 (0.02,0.06) | -0.9 (-1.07,-0.73) | 2 (1,3) | 4.18 (2.56,6.33) | 1 (0,2) | 3.04 (1.53,4.99) | -0.9 (-1.07,-0.73) |
| Moldova | 10 (9,12) | 0.84 (0.71,0.99) | 2 (2,3) | 0.4 (0.31,0.52) | -1.24 (-1.93,-0.54) | 3 (3,4) | 0.26 (0.22,0.3) | 0 (0,1) | 0.08 (0.06,0.1) | -2.51 (-3.08,-1.94) | 279 (243,324) | 22.55 (19.63,26.18) | 37 (30,48) | 7.13 (5.67,9.17) | -2.49 (-3.07,-1.91) |

| | | | | | | | | | | | | | | | |
|---|---|---|---|---|---|---|---|---|---|---|---|---|---|---|---|
| Monaco | 0 (0,0) | 0.99 (0.66,1.45) | 0 (0,0) | 1.46 (1.04,1.98) | -0.13 (-0.67,0.41) | 0 (0,0) | 0.2 (0.14,0.28) | 0 (0,0) | 0.19 (0.15,0.25) | -1.61 (-2.22,-1.01) | 1 (0,1) | 17.56 (12.27,25.05) | 1 (1,1) | 17.14 (12.91,22.18) | -1.6 (-2.2,-0.98) |
| Mongolia | 1 (1,2) | 0.13 (0.07,0.19) | 5 (3,7) | 0.44 (0.31,0.6) | 7.1 (5.63,8.58) | 1 (0,1) | 0.07 (0.04,0.09) | 2 (1,2) | 0.17 (0.12,0.22) | 5.82 (4.41,7.24) | 52 (31,73) | 5.83 (3.43,8.16) | 155 (115,210) | 14.27 (10.57,19.34) | 5.81 (4.4,7.23) |
| Montenegro | 1 (1,2) | 0.71 (0.48,0.99) | 0 (0,0) | 0.29 (0.18,0.42) | -1.88 (-2.21,-1.54) | 0 (0,0) | 0.17 (0.12,0.24) | 0 (0,0) | 0.06 (0.04,0.09) | -2.63 (-2.99,-2.27) | 24 (17,34) | 15.12 (10.47,21.06) | 6 (4,8) | 5.05 (3.18,7.4) | -2.64 (-3,-2.28) |
| Morocco | 13 (7,20) | 0.13 (0.07,0.2) | 10 (6,16) | 0.1 (0.06,0.17) | -0.22 (-0.47,0.02) | 4 (2,6) | 0.04 (0.02,0.06) | 2 (1,4) | 0.02 (0.01,0.04) | -1.27 (-1.55,-1) | 343 (202,522) | 3.5 (2.06,5.34) | 192 (107,323) | 1.96 (1.1,3.3) | -1.26 (-1.54,-0.98) |
| Mozambique | 24 (16,31) | 0.38 (0.26,0.51) | 40 (15,75) | 0.28 (0.1,0.53) | -0.79 (-0.91,-0.67) | 15 (10,20) | 0.24 (0.17,0.32) | 22 (8,39) | 0.15 (0.06,0.28) | -1.22 (-1.35,-1.09) | 1325 (920,1755) | 21.36 (14.82,28.29) | 1942 (727,3504) | 13.61 (5.1,24.56) | -1.22 (-1.35,-1.09) |
| Myanmar | 69 (22,122) | 0.47 (0.15,0.83) | 80 (48,121) | 0.51 (0.31,0.78) | 0.34 (0.25,0.44) | 32 (10,56) | 0.22 (0.07,0.38) | 28 (18,42) | 0.18 (0.11,0.27) | -0.67 (-0.81,-0.54) | 2787 (859,4866) | 18.86 (5.82,32.93) | 2412 (1548,3644) | 15.45 (9.92,23.34) | -0.67 (-0.81,-0.54) |
| Namibia | 2 (1,3) | 0.32 (0.19,0.47) | 5 (3,7) | 0.57 (0.37,0.83) | 2.92 (2.19,3.65) | 1 (1,2) | 0.18 (0.11,0.27) | 2 (1,3) | 0.24 (0.16,0.35) | 1.87 (1.22,2.52) | 94 (57,140) | 15.67 (9.5,23.27) | 170 (113,251) | 20.58 (13.69,30.37) | 1.88 (1.23,2.54) |
| Nauru | 0 (0,0) | 0.23 (0.14,0.36) | 0 (0,0) | 0.24 (0.16,0.37) | -0.05 (-0.2,0.09) | 0 (0,0) | 0.09 (0.06,0.14) | 0 (0,0) | 0.09 (0.05,0.13) | -0.33 (-0.6,-0.05) | 0 (0,1) | 8.03 (4.97,12.68) | 0 (0,0) | 7.77 (4.85,11.81) | -0.33 (-0.61,-0.06) |
| Nepal | 18 (7,31) | 0.21 (0.08,0.37) | 14 (10,21) | 0.15 (0.1,0.22) | -0.96 (-1.12,-0.8) | 11 (4,19) | 0.13 (0.05,0.23) | 6 (4,9) | 0.06 (0.04,0.09) | -2.12 (-2.31,-1.94) | 967 (380,1703) | 11.48 (4.51,20.22) | 515 (354,751) | 5.58 (3.84,8.14) | -2.14 (-2.32,-1.96) |

| | | | | | | | | | | | | | | | |
|---|---|---|---|---|---|---|---|---|---|---|---|---|---|---|---|
| Netherlands | 18 (16,22) | 0.68 (0.59,0.79) | 17 (14,21) | 0.64 (0.52,0.8) | -0.05 (-0.36,0.26) | 5 (4,5) | 0.17 (0.15,0.19) | 2 (2,3) | 0.09 (0.08,0.1) | -2.14 (-2.37,-1.91) | 400 (364,442) | 14.68 (13.37,16.23) | 211 (180,245) | 7.87 (6.7,9.14) | -2.09 (-2.32,-1.86) |
| New Zealand | 3 (3,4) | 0.43 (0.37,0.5) | 3 (2,4) | 0.3 (0.25,0.37) | -0.82 (-1.4,-0.24) | 1 (1,1) | 0.1 (0.09,0.11) | 0 (0,1) | 0.05 (0.04,0.05) | -2.1 (-2.59,-1.59) | 68 (60,76) | 8.47 (7.52,9.47) | 40 (34,47) | 4.09 (3.49,4.75) | -2.06 (-2.58,-1.55) |
| Nicaragua | 10 (8,13) | 0.56 (0.42,0.71) | 5 (3,7) | 0.25 (0.17,0.36) | -1.68 (-1.94,-1.42) | 5 (4,6) | 0.27 (0.21,0.34) | 2 (1,2) | 0.08 (0.06,0.12) | -3.07 (-3.3,-2.84) | 434 (330,541) | 23.83 (18.14,29.69) | 142 (96,205) | 7.17 (4.84,10.34) | -3.09 (-3.32,-2.85) |
| Niger | 21 (11,33) | 0.52 (0.27,0.81) | 57 (33,93) | 0.45 (0.26,0.73) | -0.3 (-0.51,-0.1) | 13 (7,21) | 0.33 (0.17,0.52) | 31 (18,50) | 0.24 (0.14,0.39) | -0.95 (-1.15,-0.74) | 1185 (624,1850) | 29.15 (15.35,45.55) | 2677 (1596,4345) | 20.98 (12.5,34.04) | -0.95 (-1.16,-0.74) |
| Nigeria | 339 (223,463) | 0.87 (0.57,1.18) | 1032 (592,1517) | 1.02 (0.58,1.49) | 1.04 (0.85,1.24) | 214 (137,303) | 0.55 (0.35,0.77) | 534 (318,772) | 0.53 (0.31,0.76) | 0.3 (0.15,0.46) | 18836 (12105,26612) | 48.14 (30.94,68.02) | 46916 (27875,67729) | 46.18 (27.44,66.67) | 0.3 (0.15,0.46) |
| Niue | 0 (0,0) | 0.18 (0.12,0.29) | 0 (0,0) | 0.98 (0.59,1.39) | 2.7 (1.73,3.68) | 0 (0,0) | 0.06 (0.04,0.09) | 0 (0,0) | 0.27 (0.16,0.38) | 2.05 (1.12,2.99) | 0 (0,0) | 5.48 (3.6,8.28) | 0 (0,0) | 23.37 (14.07,33.07) | 2.05 (1.13,2.98) |
| North Korea | 51 (32,75) | 0.86 (0.55,1.26) | 24 (10,40) | 0.5 (0.21,0.84) | -1.39 (-1.61,-1.16) | 16 (10,23) | 0.26 (0.17,0.38) | 6 (3,11) | 0.13 (0.05,0.22) | -2.11 (-2.28,-1.94) | 1390 (881,2000) | 23.36 (14.81,33.63) | 536 (228,919) | 11.22 (4.78,19.26) | -2.14 (-2.31,-1.97) |
| North Macedonia | 2 (1,2) | 0.29 (0.21,0.39) | 1 (0,1) | 0.19 (0.12,0.27) | 0.17 (-0.26,0.6) | 1 (0,1) | 0.1 (0.08,0.14) | 0 (0,0) | 0.05 (0.03,0.07) | -1.25 (-1.67,-0.83) | 48 (36,63) | 9.09 (6.77,12.03) | 13 (9,19) | 4.1 (2.65,5.94) | -1.26 (-1.68,-0.83) |
| Northern Mariana Islands | 0 (0,0) | 0.1 (0.06,0.16) | 0 (0,0) | 0.09 (0.06,0.13) | -0.75 (-1.19,-0.32) | 0 (0,0) | 0.03 (0.02,0.04) | 0 (0,0) | 0.02 (0.02,0.03) | -0.87 (-1.28,-0.46) | 0 (0,0) | 2.43 (1.44,3.82) | 0 (0,0) | 1.99 (1.39,2.8) | -0.91 (-1.32,-0.49) |

| | | | | | | | | | | | | | | | | |
|---|---|---|---|---|---|---|---|---|---|---|---|---|---|---|---|---|
| Norway | 5 (5,6) | 0.67 (0.57,0.8) | 3 (2,4) | 0.33 (0.26,0.42) | -1.59 (-2.17,-1.01) | 1 (1,1) | 0.15 (0.14,0.16) | 0 (0,0) | 0.04 (0.03,0.04) | -3.77 (-4.21,-3.32) | 106 (98,114) | 13.26 (12.32,14.24) | 31 (28,36) | 3.4 (3.02,3.89) | -3.75 (-4.2,-3.29) |
| Oman | 2 (1,3) | 0.21 (0.13,0.3) | 3 (2,3) | 0.21 (0.16,0.28) | 1.52 (0.99,2.05) | 0 (0,1) | 0.05 (0.03,0.06) | 0 (0,0) | 0.03 (0.02,0.04) | -0.15 (-0.67,0.37) | 35 (25,48) | 4.2 (3,5.66) | 32 (25,41) | 2.64 (2.05,3.35) | -0.09 (-0.62,0.44) |
| Pakistan | 146 (106,193) | 0.3 (0.21,0.39) | 416 (252,611) | 0.49 (0.3,0.71) | 2.44 (2.15,2.73) | 88 (63,115) | 0.18 (0.13,0.23) | 200 (122,299) | 0.23 (0.14,0.35) | 1.67 (1.41,1.93) | 7730 (5540,10090) | 15.7 (11.25,20.49) | 17487 (10585,26276) | 20.47 (12.39,30.75) | 1.67 (1.41,1.93) |
| Palau | 0 (0,0) | 0.07 (0.04,0.1) | 0 (0,0) | 0.06 (0.04,0.09) | -0.2 (-0.38,-0.03) | 0 (0,0) | 0.02 (0.01,0.03) | 0 (0,0) | 0.02 (0.01,0.03) | -0.5 (-0.71,-0.29) | 0 (0,0) | 1.92 (1.22,2.91) | 0 (0,0) | 1.61 (1.1,2.31) | -0.5 (-0.71,-0.29) |
| Palestine | 8 (4,13) | 0.82 (0.43,1.36) | 12 (8,18) | 0.66 (0.44,0.97) | -0.14 (-0.43,0.14) | 2 (1,3) | 0.18 (0.11,0.28) | 2 (1,3) | 0.11 (0.08,0.15) | -1.18 (-1.43,-0.92) | 157 (92,240) | 16.23 (9.51,24.74) | 176 (124,240) | 9.45 (6.63,12.87) | -1.18 (-1.44,-0.93) |
| Panama | 5 (4,6) | 0.63 (0.52,0.77) | 9 (7,11) | 0.8 (0.63,0.99) | 0.87 (0.76,0.97) | 2 (2,3) | 0.26 (0.22,0.3) | 2 (2,3) | 0.21 (0.17,0.26) | -0.43 (-0.55,-0.3) | 186 (159,218) | 22.32 (19.02,26.12) | 212 (171,259) | 18.39 (14.84,22.44) | -0.41 (-0.53,-0.29) |
| Papua New Guinea | 2 (1,4) | 0.14 (0.08,0.23) | 6 (4,11) | 0.16 (0.09,0.27) | 0.33 (0.13,0.53) | 1 (1,2) | 0.06 (0.03,0.1) | 3 (2,4) | 0.06 (0.04,0.11) | 0.36 (0.11,0.61) | 85 (50,145) | 5.01 (2.96,8.54) | 222 (136,366) | 5.68 (3.46,9.33) | 0.36 (0.11,0.61) |
| Paraguay | 10 (8,13) | 0.63 (0.47,0.8) | 15 (10,22) | 0.75 (0.51,1.07) | 1.3 (0.97,1.62) | 5 (4,6) | 0.28 (0.22,0.36) | 5 (3,7) | 0.24 (0.16,0.35) | 0.22 (-0.11,0.54) | 410 (319,519) | 24.56 (19.09,31.06) | 419 (283,602) | 20.88 (14.11,29.99) | 0.21 (-0.11,0.53) |
| Peru | 64 (50,79) | 0.77 (0.61,0.95) | 50 (34,69) | 0.52 (0.36,0.72) | -0.78 (-1.08,-0.48) | 32 (26,39) | 0.39 (0.31,0.47) | 14 (10,19) | 0.14 (0.1,0.2) | -2.85 (-3.12,-2.58) | 2784 (2233,3401) | 33.54 (26.9,40.97) | 1169 (821,1609) | 12.26 (8.6,16.87) | -2.87 (-3.14,-2.6) |

| | | | | | | | | | | | | | | | |
|---|---|---|---|---|---|---|---|---|---|---|---|---|---|---|---|
| Philippines | 192 (109,281) | 0.76 (0.43,1.12) | 200 (158,251) | 0.59 (0.46,0.74) | -0.32 (-0.53,-0.12) | 68 (40,99) | 0.27 (0.16,0.39) | 66 (53,81) | 0.19 (0.15,0.24) | -0.46 (-0.65,-0.27) | 5902 (3443,8633) | 23.41 (13.66,34.24) | 5677 (4525,7074) | 16.7 (13.31,20.81) | -0.48 (-0.67,-0.28) |
| Poland | 58 (46,68) | 0.6 (0.48,0.71) | 19 (16,24) | 0.33 (0.27,0.4) | -1.25 (-1.5,-1) | 23 (19,26) | 0.24 (0.2,0.27) | 5 (4,5) | 0.08 (0.07,0.09) | -3.05 (-3.25,-2.85) | 1987 (1659,2213) | 20.75 (17.32,23.11) | 395 (342,451) | 6.72 (5.81,7.65) | -3.02 (-3.23,-2.82) |
| Portugal | 11 (9,13) | 0.51 (0.43,0.6) | 6 (5,7) | 0.45 (0.37,0.55) | -1.08 (-1.37,-0.79) | 3 (2,3) | 0.13 (0.12,0.15) | 1 (1,1) | 0.05 (0.05,0.06) | -3.6 (-3.95,-3.24) | 242 (215,272) | 11.41 (10.14,12.85) | 65 (54,77) | 4.76 (3.99,5.65) | -3.52 (-3.87,-3.17) |
| Puerto Rico | 4 (3,5) | 0.41 (0.34,0.49) | 1 (1,2) | 0.29 (0.23,0.35) | -0.45 (-0.77,-0.13) | 1 (1,2) | 0.15 (0.13,0.17) | 0 (0,0) | 0.06 (0.05,0.07) | -2.25 (-2.54,-1.95) | 124 (107,142) | 12.49 (10.76,14.27) | 23 (19,28) | 5.18 (4.24,6.24) | -2.21 (-2.5,-1.92) |
| Qatar | 1 (1,1) | 0.64 (0.43,0.93) | 3 (2,4) | 0.58 (0.36,0.87) | 0.31 (0.01,0.62) | 0 (0,0) | 0.11 (0.08,0.16) | 0 (0,0) | 0.06 (0.04,0.09) | -1.77 (-1.95,-1.59) | 13 (9,17) | 10.07 (7.25,13.69) | 26 (17,39) | 5.34 (3.52,7.85) | -1.69 (-1.88,-1.5) |
| Republic of Congo | 2 (1,3) | 0.19 (0.1,0.32) | 3 (2,5) | 0.17 (0.12,0.24) | -0.16 (-0.4,0.08) | 1 (1,2) | 0.12 (0.06,0.2) | 2 (1,2) | 0.08 (0.06,0.12) | -1.05 (-1.29,-0.81) | 110 (58,187) | 10.42 (5.51,17.77) | 133 (92,193) | 6.92 (4.79,10.01) | -1.08 (-1.32,-0.84) |
| Romania | 31 (26,36) | 0.55 (0.46,0.65) | 13 (10,16) | 0.42 (0.33,0.53) | -0.68 (-1.34,-0.02) | 12 (10,13) | 0.21 (0.18,0.24) | 3 (2,4) | 0.1 (0.08,0.12) | -2.3 (-2.91,-1.69) | 1001 (858,1146) | 17.98 (15.41,20.59) | 256 (207,316) | 8.5 (6.87,10.49) | -2.28 (-2.87,-1.67) |
| Russia | 402 (386,419) | 1.16 (1.11,1.21) | 121 (110,131) | 0.46 (0.42,0.5) | -1.75 (-2.3,-1.2) | 106 (102,109) | 0.3 (0.29,0.31) | 21 (20,23) | 0.08 (0.07,0.09) | -3.23 (-3.73,-2.72) | 9206 (8866,9546) | 26.53 (25.55,27.51) | 1884 (1711,2042) | 7.22 (6.56,7.83) | -3.16 (-3.68,-2.63) |
| Rwanda | 30 (20,41) | 0.87 (0.59,1.21) | 33 (14,58) | 0.66 (0.29,1.17) | -0.66 (-0.97,-0.35) | 19 (13,27) | 0.57 (0.39,0.8) | 16 (7,28) | 0.32 (0.14,0.56) | -1.79 (-2.09,-1.49) | 1710 (1153,2375) | 50.41 (33.97,70) | 1386 (607,2419) | 27.89 (12.21,48.68) | -1.79 (-2.08,-1.49) |

| | | | | | | | | | | | | | | | |
|---|---|---|---|---|---|---|---|---|---|---|---|---|---|---|---|
| Saint Kitts and Nevis | 0 (0,0) | 0.57 (0.48,0.69) | 0 (0,0) | 0.71 (0.56,0.89) | 1.24 (0.94,1.53) | 0 (0,0) | 0.3 (0.25,0.34) | 0 (0,0) | 0.24 (0.19,0.29) | -0.21 (-0.43,0.01) | 4 (3,4) | 25.25 (21.67,29.36) | 2 (2,2) | 20.25 (16.28,24.99) | -0.21 (-0.43,0.01) |
| Saint Lucia | 0 (0,0) | 0.41 (0.33,0.49) | 0 (0,0) | 0.51 (0.4,0.66) | 0.6 (0.36,0.84) | 0 (0,0) | 0.2 (0.16,0.24) | 0 (0,0) | 0.17 (0.13,0.22) | -0.58 (-0.8,-0.35) | 9 (7,11) | 16.94 (14,20.39) | 4 (3,5) | 14.31 (11.21,18.31) | -0.61 (-0.83,-0.38) |
| Saint Vincent and the Grenadines | 0 (0,0) | 0.55 (0.44,0.67) | 0 (0,0) | 0.68 (0.53,0.86) | 0.71 (0.25,1.18) | 0 (0,0) | 0.25 (0.21,0.3) | 0 (0,0) | 0.23 (0.18,0.27) | -0.29 (-0.77,0.2) | 9 (7,11) | 21.5 (17.52,25.7) | 5 (4,6) | 19.03 (15.2,22.81) | -0.32 (-0.81,0.16) |
| Samoa | 0 (0,0) | 0.17 (0.1,0.26) | 0 (0,0) | 0.16 (0.07,0.29) | -0.21 (-0.38,-0.05) | 0 (0,0) | 0.06 (0.04,0.09) | 0 (0,0) | 0.05 (0.02,0.09) | -0.59 (-0.74,-0.45) | 4 (2,6) | 5.2 (3.24,7.79) | 4 (2,6) | 4.4 (2.04,7.74) | -0.59 (-0.73,-0.44) |
| San Marino | 0 (0,0) | 0.56 (0.37,0.8) | 0 (0,0) | 0.34 (0.22,0.51) | -1.29 (-1.57,-1.01) | 0 (0,0) | 0.12 (0.08,0.16) | 0 (0,0) | 0.05 (0.03,0.07) | -2.71 (-2.84,-2.58) | 0 (0,1) | 10.13 (6.92,13.96) | 0 (0,0) | 4.05 (2.7,5.88) | -2.69 (-2.84,-2.55) |
| Sao Tome and Principe | 1 (0,1) | 1.01 (0.63,1.56) | 0 (0,1) | 0.62 (0.13,1.4) | -0.97 (-1.76,-0.18) | 0 (0,1) | 0.59 (0.37,0.93) | 0 (0,0) | 0.27 (0.06,0.61) | -1.95 (-2.71,-1.18) | 30 (18,46) | 52.11 (32.49,81.95) | 18 (4,42) | 23.71 (4.75,53.84) | -1.97 (-2.74,-1.19) |
| Saudi Arabia | 19 (13,27) | 0.29 (0.2,0.42) | 13 (8,21) | 0.18 (0.11,0.28) | -1.49 (-1.62,-1.35) | 6 (4,8) | 0.09 (0.06,0.12) | 2 (1,4) | 0.03 (0.02,0.05) | -3.41 (-3.52,-3.3) | 513 (358,722) | 7.83 (5.46,11.02) | 212 (123,336) | 2.8 (1.63,4.44) | -3.41 (-3.52,-3.29) |
| Senegal | 20 (14,29) | 0.55 (0.39,0.79) | 36 (17,62) | 0.56 (0.26,0.97) | 0.57 (0.22,0.92) | 12 (8,17) | 0.33 (0.23,0.47) | 17 (8,29) | 0.27 (0.13,0.46) | -0.09 (-0.43,0.26) | 1069 (743,1497) | 29.27 (20.34,41) | 1491 (703,2569) | 23.44 (11.05,40.39) | -0.09 (-0.44,0.26) |
| Serbia | 13 (7,20) | 0.58 (0.32,0.91) | 3 (2,4) | 0.22 (0.14,0.33) | -3.4 (-3.87,-2.93) | 5 (3,7) | 0.21 (0.12,0.33) | 1 (0,1) | 0.06 (0.04,0.08) | -4.81 (-5.33,-4.3) | 395 (224,612) | 18.22 (10.31,28.24) | 63 (41,95) | 4.78 (3.11,7.19) | -4.82 (-5.33,-4.3) |

| | | | | | | | | | | | | | | | | |
|---|---|---|---|---|---|---|---|---|---|---|---|---|---|---|---|---|
| Seychelles | 0 (0,0) | 0.23 (0.16,0.31) | 0 (0,0) | 0.23 (0.16,0.3) | -0.38 (-0.96,0.19) | 0 (0,0) | 0.07 (0.05,0.1) | 0 (0,0) | 0.05 (0.04,0.07) | -1.14 (-1.72,-0.55) | 2 (1,2) | 6.32 (4.59,8.28) | 1 (1,1) | 4.87 (3.56,6.33) | -1.09 (-1.67,-0.51) |
| Sierra Leone | 10 (6,15) | 0.53 (0.31,0.83) | 22 (14,34) | 0.61 (0.39,0.95) | 0.81 (0.58,1.04) | 6 (3,9) | 0.33 (0.19,0.51) | 11 (7,17) | 0.31 (0.2,0.49) | 0.22 (-0.01,0.45) | 521 (305,808) | 28.74 (16.85,44.57) | 972 (627,1524) | 27.19 (17.52,42.62) | 0.21 (-0.02,0.44) |
| Singapore | 2 (2,2) | 0.29 (0.24,0.33) | 2 (2,3) | 0.31 (0.24,0.39) | 1.05 (0.68,1.42) | 1 (1,1) | 0.09 (0.08,0.1) | 0 (0,0) | 0.04 (0.03,0.05) | -1.76 (-2.09,-1.43) | 52 (46,59) | 7.95 (7.02,9.02) | 31 (25,37) | 3.79 (3.07,4.61) | -1.7 (-2.03,-1.37) |
| Slovakia | 9 (7,12) | 0.68 (0.5,0.89) | 8 (5,11) | 0.9 (0.63,1.3) | 1.47 (1.25,1.7) | 2 (2,3) | 0.16 (0.13,0.2) | 1 (1,2) | 0.15 (0.11,0.21) | 0.16 (-0.04,0.36) | 189 (147,234) | 14.24 (11.07,17.68) | 113 (81,158) | 13.18 (9.5,18.5) | 0.23 (0.03,0.43) |
| Slovenia | 2 (1,2) | 0.42 (0.35,0.5) | 1 (1,2) | 0.43 (0.3,0.58) | 0.95 (0.5,1.41) | 0 (0,0) | 0.1 (0.08,0.11) | 0 (0,0) | 0.06 (0.04,0.08) | -1.05 (-1.44,-0.66) | 36 (30,41) | 8.6 (7.36,9.9) | 16 (12,21) | 5.06 (3.76,6.61) | -1 (-1.39,-0.6) |
| Solomon Islands | 0 (0,0) | 0.08 (0.04,0.14) | 0 (0,0) | 0.1 (0.04,0.16) | 0.5 (0.07,0.94) | 0 (0,0) | 0.04 (0.02,0.06) | 0 (0,0) | 0.04 (0.02,0.07) | 0.31 (-0.12,0.75) | 5 (3,8) | 3.11 (1.77,5.37) | 9 (4,15) | 3.48 (1.61,5.86) | 0.3 (-0.13,0.74) |
| Somalia | 19 (10,31) | 0.49 (0.25,0.79) | 26 (10,46) | 0.25 (0.1,0.45) | -1.47 (-1.87,-1.07) | 13 (7,20) | 0.32 (0.17,0.52) | 16 (7,28) | 0.15 (0.06,0.27) | -1.77 (-2.17,-1.38) | 1110 (575,1794) | 28.5 (14.77,46.06) | 1362 (577,2412) | 13.18 (5.59,23.35) | -1.78 (-2.17,-1.38) |
| South Africa | 56 (35,76) | 0.41 (0.26,0.56) | 66 (53,81) | 0.44 (0.35,0.53) | 0.65 (0.14,1.16) | 27 (17,36) | 0.2 (0.13,0.27) | 25 (20,31) | 0.17 (0.13,0.2) | -0.19 (-0.59,0.2) | 2318 (1480,3153) | 17.03 (10.87,23.16) | 2163 (1705,2664) | 14.22 (11.21,17.52) | -0.18 (-0.59,0.24) |
| South Korea | 30 (22,39) | 0.26 (0.2,0.34) | 15 (10,21) | 0.24 (0.16,0.35) | 0.04 (-0.26,0.35) | 12 (9,15) | 0.1 (0.08,0.13) | 2 (1,3) | 0.03 (0.02,0.05) | -3.44 (-3.59,-3.29) | 985 (748,1260) | 8.66 (6.58,11.08) | 185 (121,260) | 3.04 (2,4.28) | -3.32 (-3.47,-3.17) |

| | | | | | | | | | | | | | | | |
|---|---|---|---|---|---|---|---|---|---|---|---|---|---|---|---|
| South Sudan | 26 (12,47) | 0.99 (0.47,1.8) | 38 (19,60) | 0.89 (0.45,1.39) | 0.42 (-0.02,0.86) | 16 (8,28) | 0.6 (0.29,1.07) | 19 (10,30) | 0.44 (0.23,0.69) | -0.23 (-0.65,0.2) | 1384 (674,2470) | 52.74 (25.7,94.14) | 1662 (872,2610) | 38.69 (20.3,60.76) | -0.23 (-0.66,0.19) |
| Spain | 46 (39,55) | 0.59 (0.5,0.7) | 41 (33,52) | 0.63 (0.51,0.8) | 0.04 (-0.28,0.37) | 8 (7,10) | 0.11 (0.09,0.12) | 4 (3,5) | 0.06 (0.05,0.07) | -1.9 (-2.05,-1.75) | 732 (649,829) | 9.34 (8.28,10.58) | 362 (309,429) | 5.58 (4.77,6.62) | -1.81 (-1.98,-1.65) |
| Sri Lanka | 49 (37,63) | 0.88 (0.67,1.13) | 9 (6,13) | 0.18 (0.12,0.26) | -6.91 (-7.9,-5.9) | 15 (12,19) | 0.28 (0.22,0.35) | 2 (1,3) | 0.04 (0.03,0.05) | -8.27 (-9.28,-7.24) | 1314 (1030,1666) | 23.74 (18.61,30.11) | 168 (112,239) | 3.29 (2.2,4.68) | -8.21 (-9.22,-7.19) |
| Sudan | 85 (25,184) | 0.95 (0.29,2.07) | 149 (97,232) | 0.9 (0.59,1.4) | 0.34 (0.1,0.59) | 26 (9,51) | 0.29 (0.1,0.58) | 33 (23,48) | 0.2 (0.14,0.29) | -0.71 (-0.93,-0.49) | 2288 (757,4530) | 25.73 (8.51,50.95) | 2933 (2008,4236) | 17.68 (12.11,25.53) | -0.72 (-0.94,-0.5) |
| Suriname | 1 (0,1) | 0.52 (0.35,0.69) | 1 (1,1) | 0.54 (0.39,0.75) | 0.43 (0.14,0.71) | 0 (0,0) | 0.27 (0.19,0.36) | 0 (0,0) | 0.22 (0.16,0.3) | -0.48 (-0.75,-0.2) | 31 (21,40) | 23.41 (16.48,30.91) | 27 (19,37) | 18.59 (13.36,25.92) | -0.49 (-0.76,-0.22) |
| Swaziland | 1 (1,2) | 0.34 (0.18,0.53) | 2 (1,3) | 0.45 (0.29,0.69) | 1.28 (0.77,1.79) | 1 (0,1) | 0.19 (0.1,0.3) | 1 (1,1) | 0.21 (0.14,0.33) | 0.76 (0.36,1.17) | 63 (32,100) | 16.24 (8.35,25.83) | 74 (48,117) | 18.04 (11.75,28.26) | 0.75 (0.34,1.17) |
| Sweden | 9 (8,11) | 0.61 (0.51,0.7) | 6 (5,7) | 0.32 (0.26,0.4) | -0.97 (-1.46,-0.47) | 3 (3,3) | 0.19 (0.16,0.21) | 1 (1,1) | 0.06 (0.05,0.07) | -2.72 (-3.11,-2.32) | 248 (219,279) | 16.06 (14.17,18.1) | 92 (76,111) | 5.03 (4.15,6.11) | -2.67 (-3.08,-2.27) |
| Switzerland | 8 (7,10) | 0.69 (0.58,0.83) | 8 (6,10) | 0.58 (0.44,0.75) | -0.77 (-0.95,-0.59) | 1 (1,1) | 0.09 (0.08,0.1) | 1 (1,1) | 0.05 (0.04,0.06) | -2.19 (-2.35,-2.02) | 93 (83,106) | 8.09 (7.16,9.18) | 59 (48,72) | 4.42 (3.57,5.41) | -2.14 (-2.3,-1.99) |
| Syria | 48 (29,76) | 0.81 (0.5,1.29) | 24 (14,40) | 0.65 (0.37,1.1) | -0.08 (-0.48,0.32) | 12 (8,19) | 0.2 (0.13,0.31) | 4 (2,5) | 0.1 (0.07,0.14) | -1.82 (-2.13,-1.5) | 1065 (679,1635) | 17.99 (11.46,27.6) | 312 (211,446) | 8.52 (5.75,12.18) | -1.83 (-2.15,-1.51) |

| | | | | | | | | | | | | | | | |
|---|---|---|---|---|---|---|---|---|---|---|---|---|---|---|---|
| Taiwan (Province of China) | 34 (29,40) | 0.61 (0.52,0.72) | 31 (25,39) | 1.06 (0.84,1.31) | 1.54 (1.04,2.04) | 6 (6,7) | 0.11 (0.1,0.13) | 4 (3,4) | 0.13 (0.11,0.15) | 0.11 (-0.38,0.6) | 545 (496,607) | 9.9 (9.01,11.03) | 333 (279,389) | 11.3 (9.45,13.19) | 0.14 (-0.35,0.64) |
| Tajikistan | 16 (7,28) | 0.68 (0.32,1.22) | 18 (12,27) | 0.51 (0.34,0.74) | -0.8 (-1.28,-0.31) | 7 (4,13) | 0.32 (0.15,0.57) | 8 (5,11) | 0.22 (0.14,0.31) | -1.06 (-1.46,-0.66) | 647 (312,1166) | 27.87 (13.46,50.23) | 681 (450,972) | 19.01 (12.57,27.13) | -1.05 (-1.46,-0.65) |
| Tanzania | 123 (88,161) | 1.02 (0.73,1.33) | 238 (113,410) | 0.97 (0.46,1.68) | 0.56 (0.29,0.83) | 72 (52,93) | 0.59 (0.43,0.77) | 112 (52,195) | 0.46 (0.21,0.8) | -0.12 (-0.39,0.16) | 6309 (4567,8216) | 52.24 (37.82,68.04) | 9828 (4583,17169) | 40.28 (18.78,70.36) | -0.12 (-0.39,0.15) |
| Thailand | 55 (37,75) | 0.32 (0.22,0.45) | 45 (34,59) | 0.46 (0.35,0.6) | 1.1 (0.92,1.28) | 16 (11,21) | 0.1 (0.07,0.13) | 9 (7,12) | 0.09 (0.07,0.12) | -0.2 (-0.42,0.01) | 1393 (964,1869) | 8.27 (5.72,11.08) | 779 (604,1025) | 7.98 (6.19,10.5) | -0.21 (-0.43,0.01) |
| Timor-Leste | 2 (0,3) | 0.47 (0.14,0.87) | 2 (1,3) | 0.33 (0.23,0.48) | -1.37 (-1.7,-1.03) | 1 (0,1) | 0.21 (0.06,0.37) | 1 (0,1) | 0.12 (0.09,0.18) | -2.05 (-2.37,-1.72) | 61 (18,110) | 18.36 (5.55,32.98) | 56 (39,83) | 10.72 (7.53,15.85) | -2.08 (-2.4,-1.75) |
| Togo | 7 (4,10) | 0.38 (0.24,0.58) | 16 (7,28) | 0.47 (0.22,0.85) | 0.97 (0.82,1.12) | 4 (3,6) | 0.23 (0.14,0.35) | 8 (4,14) | 0.23 (0.11,0.42) | 0.34 (0.19,0.48) | 350 (220,543) | 19.88 (12.5,30.82) | 662 (312,1207) | 20 (9.42,36.49) | 0.33 (0.18,0.48) |
| Tokelau | 0 (0,0) | 0.15 (0.09,0.24) | 0 (0,0) | 1.29 (0.43,2.27) | 2.27 (0.36,4.22) | 0 (0,0) | 0.05 (0.03,0.09) | 0 (0,0) | 0.39 (0.13,0.7) | 1.49 (-0.44,3.47) | 0 (0,0) | 4.83 (2.78,7.67) | 0 (0,0) | 34.6 (11.33,61.53) | 1.49 (-0.44,3.46) |
| Tonga | 0 (0,0) | 0.13 (0.08,0.19) | 0 (0,0) | 0.17 (0.11,0.26) | 0.79 (0.43,1.14) | 0 (0,0) | 0.04 (0.03,0.06) | 0 (0,0) | 0.05 (0.03,0.08) | 0.54 (0.14,0.95) | 2 (1,2) | 3.68 (2.31,5.72) | 2 (1,3) | 4.56 (2.92,6.8) | 0.54 (0.14,0.95) |
| Trinidad and Tobago | 3 (2,3) | 0.62 (0.52,0.72) | 1 (1,2) | 0.51 (0.4,0.66) | 0.14 (-0.15,0.43) | 1 (1,1) | 0.29 (0.25,0.33) | 0 (0,1) | 0.17 (0.13,0.21) | -1.15 (-1.42,-0.87) | 100 (86,114) | 24.56 (21.24,28.1) | 39 (31,49) | 14.18 (11.34,18.13) | -1.13 (-1.4,-0.85) |

| | | | | | | | | | | | | | | | |
|---|---|---|---|---|---|---|---|---|---|---|---|---|---|---|---|
| Tunisia | 26 (15,38) | 0.85 (0.49,1.23) | 14 (9,22) | 0.52 (0.34,0.79) | -0.97 (-1.19,-0.74) | 6 (4,8) | 0.18 (0.12,0.25) | 2 (2,3) | 0.08 (0.06,0.12) | -1.97 (-2.17,-1.78) | 509 (337,699) | 16.38 (10.86,22.52) | 206 (141,304) | 7.44 (5.09,10.99) | -1.96 (-2.16,-1.76) |
| Turkey | 328 (167,521) | 1.6 (0.82,2.54) | 208 (147,293) | 1.12 (0.79,1.58) | -0.8 (-1.07,-0.53) | 78 (46,116) | 0.38 (0.22,0.57) | 26 (19,35) | 0.14 (0.11,0.19) | -3.12 (-3.39,-2.86) | 6938 (4013,10277) | 33.86 (19.59,50.16) | 2321 (1732,3064) | 12.53 (9.35,16.55) | -3.1 (-3.36,-2.83) |
| Turkmenistan | 26 (20,31) | 1.71 (1.36,2.09) | 11 (9,14) | 0.71 (0.56,0.9) | -1 (-1.85,-0.15) | 12 (10,14) | 0.79 (0.64,0.93) | 4 (3,5) | 0.26 (0.21,0.33) | -1.54 (-2.42,-0.66) | 1040 (842,1229) | 69.27 (56.13,81.86) | 347 (277,429) | 22.78 (18.19,28.18) | -1.57 (-2.46,-0.68) |
| Tuvalu | 0 (0,0) | 0.15 (0.08,0.23) | 0 (0,0) | 0.11 (0.06,0.18) | -0.81 (-0.95,-0.66) | 0 (0,0) | 0.07 (0.03,0.1) | 0 (0,0) | 0.04 (0.02,0.06) | -1.45 (-1.6,-1.31) | 0 (0,0) | 5.79 (3.06,9.22) | 0 (0,0) | 3.58 (2.04,5.65) | -1.46 (-1.61,-1.32) |
| Uganda | 78 (52,117) | 0.92 (0.62,1.39) | 214 (89,391) | 1.08 (0.45,1.97) | 1.11 (0.83,1.39) | 46 (31,69) | 0.55 (0.37,0.82) | 103 (42,190) | 0.52 (0.21,0.96) | 0.35 (0.1,0.61) | 4081 (2758,6095) | 48.46 (32.76,72.39) | 9023 (3680,16686) | 45.49 (18.55,84.12) | 0.35 (0.09,0.6) |
| UK | 54 (48,58) | 0.49 (0.44,0.53) | 55 (50,60) | 0.47 (0.42,0.51) | 0.56 (0.05,1.07) | 14 (13,15) | 0.13 (0.12,0.14) | 9 (8,10) | 0.08 (0.07,0.08) | -1.01 (-1.42,-0.6) | 1221 (1112,1286) | 11.18 (10.19,11.77) | 802 (739,858) | 6.81 (6.27,7.29) | -0.96 (-1.38,-0.55) |
| Ukraine | 121 (90,160) | 1.06 (0.79,1.41) | 42 (33,54) | 0.67 (0.53,0.85) | -0.57 (-1.07,-0.06) | 32 (25,41) | 0.28 (0.22,0.36) | 9 (8,11) | 0.15 (0.12,0.18) | -1.24 (-1.63,-0.84) | 2779 (2186,3535) | 24.43 (19.22,31.07) | 817 (679,982) | 12.87 (10.7,15.47) | -1.19 (-1.6,-0.78) |
| United Arab Emirates | 4 (2,7) | 0.66 (0.33,1.25) | 7 (5,10) | 0.55 (0.38,0.78) | 0.67 (0.17,1.17) | 1 (0,1) | 0.14 (0.08,0.23) | 1 (1,1) | 0.07 (0.06,0.09) | -0.76 (-1.2,-0.33) | 69 (39,121) | 11.77 (6.57,20.53) | 83 (65,105) | 6.23 (4.84,7.88) | -0.69 (-1.14,-0.25) |
| Uruguay | 4 (3,5) | 0.49 (0.42,0.59) | 4 (3,5) | 0.56 (0.44,0.69) | 0.16 (-0.45,0.77) | 1 (1,1) | 0.16 (0.14,0.18) | 1 (1,1) | 0.11 (0.09,0.13) | -1.38 (-1.86,-0.9) | 112 (99,128) | 13.71 (12.06,15.59) | 62 (50,74) | 9.42 (7.63,11.24) | -1.39 (-1.88,-0.9) |

| | | | | | | | | | | | | | | | |
|---|---|---|---|---|---|---|---|---|---|---|---|---|---|---|---|
| USA | 480 (463,498) | 0.86 (0.83,0.89) | 397 (369,426) | 0.67 (0.62,0.72) | -0.62 (-0.81,-0.43) | 66 (65,67) | 0.12 (0.12,0.12) | 40 (37,43) | 0.07 (0.06,0.07) | -1.61 (-1.72,-1.5) | 5814 (5681,5954) | 10.4 (10.16,10.65) | 3574 (3316,3837) | 6.01 (5.58,6.46) | -1.57 (-1.68,-1.45) |
| Uzbekistan | 38 (28,51) | 0.45 (0.33,0.6) | 44 (33,58) | 0.44 (0.32,0.58) | 0.42 (0.04,0.79) | 16 (12,21) | 0.19 (0.14,0.25) | 16 (12,21) | 0.16 (0.12,0.21) | -0.2 (-0.48,0.08) | 1417 (1053,1880) | 16.57 (12.31,21.98) | 1391 (1035,1812) | 13.78 (10.25,17.95) | -0.21 (-0.5,0.08) |
| Vanuatu | 0 (0,0) | 0.07 (0.04,0.12) | 0 (0,0) | 0.08 (0.04,0.13) | 0.47 (0.02,0.91) | 0 (0,0) | 0.03 (0.02,0.05) | 0 (0,0) | 0.03 (0.02,0.06) | 0.45 (-0.03,0.93) | 2 (1,3) | 2.66 (1.49,4.49) | 3 (2,6) | 2.74 (1.37,4.91) | 0.44 (-0.04,0.92) |
| Venezuela | 41 (36,46) | 0.57 (0.5,0.65) | 44 (33,59) | 0.66 (0.49,0.89) | 1.16 (0.83,1.5) | 19 (17,20) | 0.26 (0.24,0.29) | 14 (11,18) | 0.21 (0.16,0.28) | -0.12 (-0.49,0.25) | 1614 (1479,1758) | 22.75 (20.85,24.78) | 1191 (903,1581) | 17.98 (13.64,23.87) | -0.11 (-0.48,0.27) |
| Vietnam | 31 (21,42) | 0.12 (0.08,0.16) | 34 (22,50) | 0.14 (0.09,0.2) | 1.29 (0.94,1.64) | 11 (7,15) | 0.04 (0.03,0.05) | 8 (5,11) | 0.03 (0.02,0.05) | -0.12 (-0.42,0.19) | 945 (657,1279) | 3.56 (2.48,4.82) | 690 (454,1011) | 2.78 (1.83,4.08) | -0.11 (-0.42,0.2) |
| Virgin Islands | 0 (0,0) | 0.62 (0.45,0.83) | 0 (0,0) | 0.39 (0.21,0.65) | -0.39 (-0.87,0.08) | 0 (0,0) | 0.26 (0.19,0.34) | 0 (0,0) | 0.12 (0.06,0.2) | -1.35 (-1.81,-0.89) | 7 (5,9) | 21.95 (16.02,28.89) | 1 (1,2) | 10.21 (5.33,17.07) | -1.3 (-1.76,-0.84) |
| Yemen | 44 (16,94) | 0.62 (0.22,1.32) | 70 (37,124) | 0.51 (0.27,0.9) | 0.07 (-0.33,0.47) | 13 (5,25) | 0.19 (0.08,0.35) | 16 (10,26) | 0.12 (0.07,0.19) | -0.92 (-1.22,-0.62) | 1161 (470,2194) | 16.36 (6.63,30.92) | 1441 (847,2253) | 10.45 (6.14,16.34) | -0.92 (-1.23,-0.61) |
| Zambia | 41 (24,60) | 1.1 (0.65,1.6) | 63 (33,102) | 0.76 (0.4,1.23) | -0.7 (-0.94,-0.45) | 25 (16,37) | 0.68 (0.42,0.97) | 31 (16,50) | 0.37 (0.19,0.61) | -1.52 (-1.72,-1.31) | 2248 (1381,3219) | 59.87 (36.78,85.73) | 2679 (1387,4402) | 32.38 (16.77,53.22) | -1.53 (-1.73,-1.32) |
| Zimbabwe | 8 (6,10) | 0.16 (0.12,0.22) | 22 (15,30) | 0.34 (0.23,0.48) | 4.17 (3.31,5.04) | 4 (3,5) | 0.09 (0.06,0.11) | 11 (7,15) | 0.17 (0.12,0.24) | 4.23 (3.34,5.13) | 363 (266,465) | 7.54 (5.52,9.65) | 935 (643,1301) | 14.86 (10.22,20.67) | 4.21 (3.32,5.1) |

UI, uncertainty interval; ASIR, age-standardized incidence rate; EAPC, estimated annual percentage change; CI, confidence interval; ASMR, age-standardized mortality rate; DALYs, disability-adjusted life years; ASDR, age-standardized disability-adjusted life years rate

**Table S4: The predicted age-standardized incidence and mortality burden of kidney cancer in children from 2022 to 2036 globally.**

| Sex | Year | Incidence cases (Age-standardized), N(95% Crl) | ASIR, Per 100,000 (95% Crl) | Mortality cases (Age-standardized), N(95% Crl) | ASMR, Per 100,000 (95% Crl) |
|---|---|---|---|---|---|
| Male | 2022 | 5122(4794,5449) | 0.49(0.47,0.52) | 1941(1777,2104) | 0.19(0.17,0.2) |
| | 2023 | 5042(4627,5456) | 0.49(0.45,0.52) | 1916(1718,2113) | 0.18(0.17,0.2) |
| | 2024 | 4958(4434,5481) | 0.48(0.43,0.53) | 1890(1649,2131) | 0.18(0.16,0.2) |
| | 2025 | 4869(4220,5518) | 0.47(0.41,0.53) | 1862(1571,2154) | 0.18(0.15,0.21) |
| | 2026 | 4779(3992,5565) | 0.46(0.39,0.54) | 1834(1487,2181) | 0.18(0.14,0.21) |
| | 2027 | 4687(3754,5620) | 0.45(0.36,0.54) | 1806(1399,2213) | 0.17(0.14,0.21) |
| | 2028 | 4594(3508,5680) | 0.45(0.34,0.55) | 1776(1306,2246) | 0.17(0.13,0.22) |
| | 2029 | 4499(3256,5741) | 0.44(0.32,0.56) | 1746(1211,2282) | 0.17(0.12,0.22) |
| | 2030 | 4402(3000,5804) | 0.43(0.29,0.57) | 1716(1113,2318) | 0.17(0.11,0.23) |
| | 2031 | 4306(2742,5869) | 0.42(0.27,0.58) | 1685(1014,2356) | 0.17(0.1,0.23) |
| | 2032 | 4209(2483,5935) | 0.42(0.25,0.59) | 1654(914,2394) | 0.16(0.09,0.24) |
| | 2033 | 4119(2228,6009) | 0.41(0.22,0.6) | 1625(815,2435) | 0.16(0.08,0.24) |
| | 2034 | 4029(1974,6084) | 0.4(0.2,0.6) | 1596(715,2477) | 0.16(0.07,0.25) |
| | 2035 | 3941(1722,6159) | 0.39(0.17,0.62) | 1568(616,2519) | 0.16(0.06,0.25) |
| | 2036 | 3854(1473,6234) | 0.39(0.15,0.63) | 1539(517,2562) | 0.15(0.05,0.26) |
| Female | 2022 | 4626(4321,4931) | 0.48(0.45,0.51) | 1234(1117,1351) | 0.13(0.12,0.14) |
| | 2023 | 4409(4023,4794) | 0.45(0.42,0.49) | 1187(1051,1323) | 0.12(0.11,0.13) |
| | 2024 | 4192(3711,4673) | 0.43(0.38,0.48) | 1140(980,1299) | 0.12(0.1,0.13) |
| | 2025 | 3977(3395,4560) | 0.41(0.35,0.47) | 1093(908,1279) | 0.11(0.09,0.13) |
| | 2026 | 3769(3081,4456) | 0.39(0.32,0.46) | 1048(834,1262) | 0.11(0.09,0.13) |

| | | | | |
|---|---|---|---|---|
| 2027 | 3568(2776,4361) | 0.37(0.29,0.45) | 1004(761,1246) | 0.1(0.08,0.13) |
| 2028 | 3374(2479,4268) | 0.35(0.26,0.44) | 960(689,1232) | 0.1(0.07,0.13) |
| 2029 | 3186(2195,4178) | 0.33(0.23,0.43) | 918(619,1218) | 0.1(0.06,0.13) |
| 2030 | 3006(1923,4089) | 0.31(0.2,0.43) | 878(551,1205) | 0.09(0.06,0.13) |
| 2031 | 2834(1666,4003) | 0.3(0.18,0.42) | 838(485,1192) | 0.09(0.05,0.12) |
| 2032 | 2672(1425,3919) | 0.28(0.15,0.41) | 801(422,1179) | 0.08(0.04,0.12) |
| 2033 | 2520(1200,3841) | 0.27(0.13,0.41) | 765(362,1168) | 0.08(0.04,0.12) |
| 2034 | 2377(989,3764) | 0.25(0.11,0.4) | 731(305,1157) | 0.08(0.03,0.12) |
| 2035 | 2241(794,3688) | 0.24(0.08,0.39) | 698(251,1145) | 0.07(0.03,0.12) |
| 2036 | 2113(613,3613) | 0.23(0.07,0.39) | 667(200,1134) | 0.07(0.02,0.12) |

Crl, credible interval; ASIR, age-standardized incidence rate; ASMR, age-standardized mortality rate
